# Decoding the Geography of Natural TBEV Microfoci in Germany: A Geostatistical Approach Based on Land-Use Patterns and Climatological Conditions

**DOI:** 10.3390/ijerph191811830

**Published:** 2022-09-19

**Authors:** Johannes P. Borde, Rüdiger Glaser, Klaus Braun, Nils Riach, Rafael Hologa, Klaus Kaier, Lidia Chitimia-Dobler, Gerhard Dobler

**Affiliations:** 1 Division of Infectious Diseases, Department of Medicine II, Faculty of Medicine, University of Freiburg Medical Center, D-79106 Freiburg im Breisgau, Germany; 2 Praxis Prof. Dr. J. Borde & Kollegen, Gesundheitszentrum Oberkirch, Am Marktplatz 8, D-77704 Oberkirch, Germany; 3 Institute of Environmental Social Sciences and Geography, University of Freiburg, Schreiberstr. 20, D-79098 Freiburg im Breisgau, Germany; 4 Medical Center, Faculty of Medicine, Institute of Medical Biometry and Statistics, University of Freiburg, Stefan-Meier-Straße 26, D-79104 Freiburg im Breisgau, Germany; 5 German National Reference Laboratory for TBEV, Bundeswehr Institute of Microbiology, Neuherbergstraße 11, D-80937 München, Germany; 6 Parasitology Unit, University of Hohenheim, Emil-Wolff-Straße 34, D-70599 Stuttgart, Germany

**Keywords:** MaxEnt, prediction model, TBE, tick-borne encephalitis, TBEV, microfocus, *Ixodes ricinus*, geostatistical approach, environmental variables, climatological data, land-use patterns

## Abstract

**Background:** Tickborne-encephalitis (TBE) is a potentially life-threating neurological disease that is mainly transmitted by ticks. The goal of the present study is to analyze the potential uniform environmental patterns of the identified TBEV microfoci in Germany. The results are used to calculate probabilities for the present distribution of TBEV microfoci in Germany based on a geostatistical model. **Methods:** We aim to consider the specification of environmental characteristics of locations of TBEV microfoci detected in Germany using open access epidemiological, geographical and climatological data sources. We use a two-step geostatistical approach, where in a first step, the characteristics of a broad set of environmental variables between the 56 TBEV microfoci and a control or comparator set of 3575 sampling points covering Germany are compared using Fisher’s Exact Test. In the second step, we select the most important variables, which are then used in a MaxEnt distribution model to calculate a high resolution (400 × 400 m) probability map for the presence of TBEV covering the entire area of Germany. **Results:** The findings from the MaxEnt prediction model indicate that multi annual actual evapotranspiration (27.0%) and multi annual hot days (22.5%) have the highest contribution to our model. These two variables are followed by four additional variables with a lower, but still important, explanatory influence: Land cover classes (19.6%), multi annual minimum air temperature (14.9%), multi annual sunshine duration (9.0%), and distance to coniferous and mixed forest border (7.0%). **Conclusions**: Our findings are based on defined TBEV microfoci with known histories of infection and the repeated confirmation of the virus in the last years, resulting in an in-depth high-resolution model/map of TBEV microfoci in Germany. Multi annual actual evapotranspiration (27%) and multi annual hot days (22.5%) have the most explanatory power in our model. The results may be used to tailor specific regional preventive measures and investigations.

## 1. Introduction

Tickborne-encephalitis (TBE) is a potentially life-threating neurological disease that is mainly transmitted by ticks [1,2]. The causative viral agent is tickborne-encephalitis virus (TBEV), which is a member of the mammalian tick-borne group in the family *Flaviviridae* [3]. In Europe, TBEV is endemic in Central Europe, in Eastern Europe, in parts of the Scandinavian countries, in the Baltic region and is genetically divided into several subtypes. Each year, in Europe [4] and Asia, there are 10,000–12,000 notified cases of TBE. In Germany [5], there are between 400–700 cases reported to the national health authorities annually, with an increasing trend. The main vector for TBEV is the hard tick *Ixodes ricinus*. Small rodents, such as *Apodemus* spp. and *Myodes* spp., are believed to be the reservoir hosts of TBEV [3], harboring the virus with a high and prolonged viraemia [6,7]. The population dynamics of these host species and its environmental influencing factors are crucial for maintaining stable zoonotic cycles of TBEV between rodents and *Ixodes* ticks [8]. To date, there is no robust ecological evidence that the concept of co-feeding is important for the stability of established zoonotic TBEV cycles in nature—as studied so far in Germany [8]. The transmission cycle occurs in the so-called microfoci, small areas with an average size of about 0.5 to 1 ha, which are stable for decades and usually do not expand or shift. The small sized areas of these transmission cycles and their stability are still not understood and, so far, environmental models do not provide plausible explanations for this spatio-temporal stability.

There are several published models regarding the prediction of human TBE cases and tick numbers based on rodent population dynamics, weather conditions, and other environmental influences such as beech mast [9,10,11]. In addition, larger game animals are associated with the appearance of TBEV as they support the tick population as hosts of adult ticks [1]. An important issue in understanding zoonotic infections is to elucidate environmental factors, which are the contributors of maintaining zoonotic cycles and transmission to humans. Various scientific approaches have been applied to investigate the role of landscapes, land use, and landcover regarding different zoonoses, including tickborne disease such as TBEV infections [12,13], often in combination with meteorological data [14,15,16]. There are only few data for the spatial prediction of future distribution areas of TBEV [17]. The majority of these studies are based on aggregated data using NUTS (Nomenclature of Territorial Units for Statistics) level 3 [18]. A recently published work from Germany generated a sub-district risk-map for TBE, exclusively for southern Germany, based on 567 probable self-reported places of infection (POI) and 41 confirmed places/foci of infection [19]. The POI were associated with specific ecological and anthropogenic aspects—the derived information resulted in a 69% sensitivity and 63% specificity [19]. It is known, that TBEV is not homogeneously distributed throughout questing ticks across all locations. TBEV is rather detected in defined geographic areas of approximately 1ha. The terms “TBEV microfocus” and “TBEV hot spot” are inconsistently used in the literature to describe these surface areas—even high incidence districts NUTS level 3 are referred to as microfocus. We prefer the term TBEV microfocus in the definition of a small (~1 ha) geographic area with a repeatedly proven presence of TBEV in ticks or in small mammals—a very similar description/definition was introduced by Nosek et al., more than 50 years ago [20,21].

In line with the findings and ideas from Nosek et al. [21], to date, there is speculation over an evolving concept of various regional TBEV microfoci embedded in a larger defined natural focus. There are only scarce data or anecdotal reports [22] on the size and composition of such TBEV microfoci, notably the determinants of their size [23,24]. It can be assumed that the focus size of approximately 1ha is defined by the presence of the reservoir host, e.g., small rodents, and its biological operating range in such a habitat. Hence, it is hypothesized that certain genetic features and transcriptome profiles of the candidate rodent reservoir host predispose for asymptomatic carriage of TBEV and for consequent transmission to *Ixodes ricinus*.

The goal of the present study is to analyze potential uniform geographical patterns of the identified TBEV microfoci in Germany. We aim to consider the specification of geographical characteristics of these locations using open access epidemiological, meteorological, and geographical data sources. The results are used to calculate probabilities for the present distribution of TBEV microfoci in Germany based on a geostatistical model.

## 2. Materials and Methods

### 2.1. Study Area

**Figure 1:**
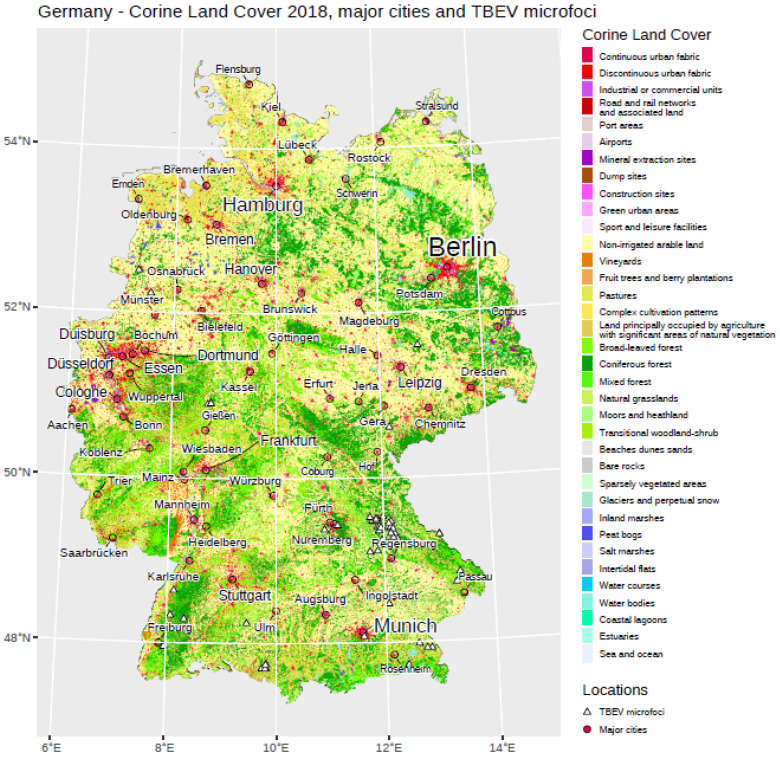
General overview regarding different land cover classes in Germany. Major cities are displayed, as well as the location of TBEV microfoci used in our analysis.

It has been shown that TBEV is mainly present in the southern parts of Germany. The federal states of Bavaria and Baden-Wuerttemberg account for more than 90% of all notified TBE cases in Germany. Therefore, most of the potential tick-collection sites are located in southern Germany. However, on the basis of newly reported TBE patient cases in the northern or eastern federal states, candidate sites have also been flagged in these parts of Germany, e.g., Lower Saxony. In total, we included in our study 56 confirmed TBEV microfoci in Germany. Figure 1 provides an overview of these microfoci and land cover obtained from Corine Land Cover products.

### 2.2. Definition of a TBEV Microfocus

TBEV microfocus—a defined geographical area in which TBEV was detected in questing ticks at least one time. In most of the locations, the presence of TBEV in ticks was repeatedly confirmed over the last 10 years. These TBEV microfoci are traced in nature based on detailed medical histories of TBE patients regarding the environmental sites where tick bites were acquired—only one site was discovered through a random search. The exact dimensions of the TBEV microfoci are unknown and there is only scarce information published in the context of this issue. However, it is suggested that such natural sites are sized at around 1 ha [22]. Overall, 56 TBEV microfoci were identified (see Table 1).

### 2.3. Epidemiological Dataset and Case Definition

Infections due to TBEV became a notifiable disease in Germany in 2001. Case definitions are issued by the Robert Koch Institute (RKI). The German case definition differs from the definition issued by the European Centre for Disease Prevention and Control (ECDC). It includes febrile forms of TBEV infections without CNS symptoms. The reported number of TBEV infections has been open to access, available in different formats, as well as spatial and temporal resolutions, since 2001 at https://www.rki.de/DE/Content/Infekt/SurvStat/survstat_node.html, accessed on 1 June 2022. The data used span the years 2001–2020 and are aggregated on county/NUTS 3 level.

### 2.4. Tick Collection, TBEV Detection, and TBEV Isolation

Ticks were collected by flagging, as described before [25]. Ticks were kept alive and morphologically identified at the TBEV national reference laboratory to species level—*Ixodes ricinus* (as previously published [25]). Ticks were pooled according to developmental stage and sex (10 nymphs and 5 adult female or male ticks) and processed for TBEV RNA and virus isolation according to Kupca et al. [26], except that, for virus isolation, A549 cells were used instead of Vero cells. The details of TBEV detection (primer details have been published before [27]) and TBEV isolation are included in the supplement material (see Supplementary S1 material and methods).

### 2.5. Environmental Raw Data Sets

To analyze the impact of environmental characteristics on the presence or absence of TBEV, free available gridded raster data have been collected. In general, these are data sets describing the geographical, climatological, and ecological situation within the study area. Primary data sets are Digital Elevation Model (DEM), Corine Land Cover (CLC), and a set of core climatological data (CLI). They were obtained from the open access Copernicus portal (https://land.copernicus.eu/, accessed on 1 June 2022) and the open data section of the Climate Data Center hosted by the German Meteorological Service DWD (Deutscher Wetterdienst). Epidemiological information regarding notified TBE cases in Germany 2001–2020 are freely available from the Robert Koch Institute (RKI).

### 2.6. Data Preparation and Processing

All primary data sets were resampled to a uniform resolution of 400 × 400 m. The reason for the specification is the observation that, not only the environmental characteristics within the TBEV microfoci (100 × 100 m) themselves have an impact on the presence or absence of TBEV, but also the condition of the surrounding landscape. Furthermore, it was given by the native spatial resolutions of the data used in this investigation. These resolutions varied between 25 × 25 m in the case of the Digital Elevation Model and 1000 × 1000 m for most of the climatological data. Together with the need for a unified spatial resolution of all raster data, a grid cell size of 400 × 400 m acted as a reasonable compromise.

**Figure 2:**
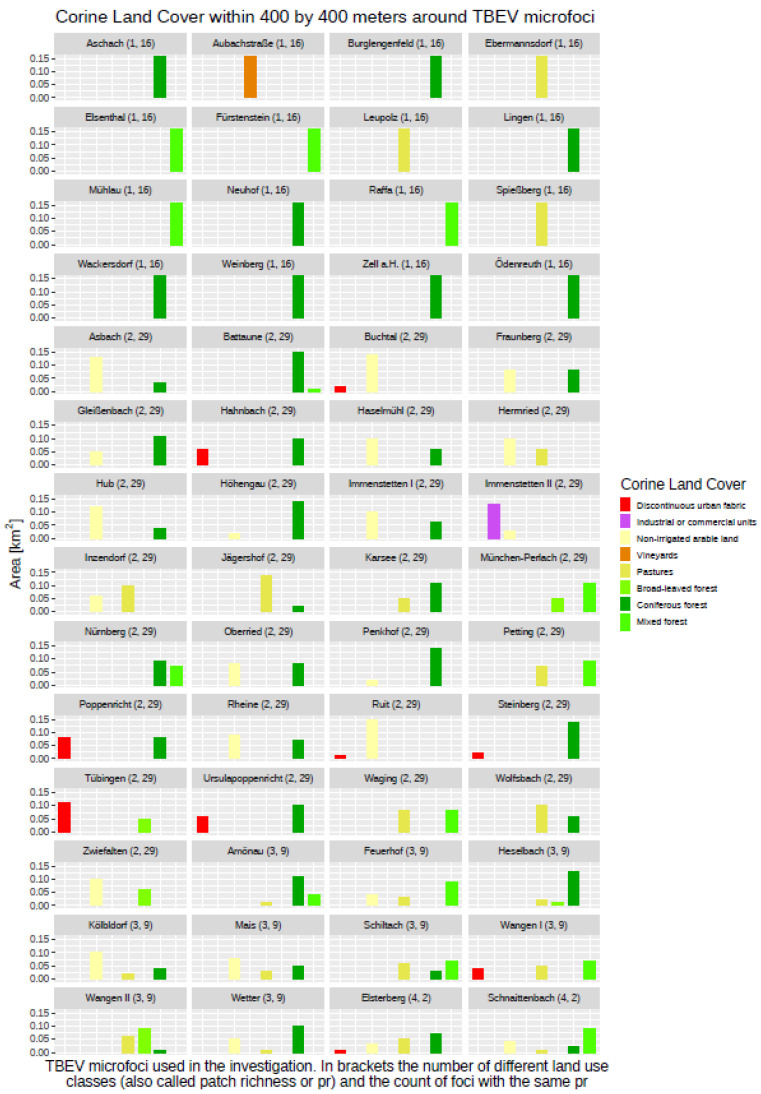
Overview regarding TBEV microfoci and the surrounding land cover classes within 400 × 400 m.

Subsequently, these gridded raster data were used to derive a couple of secondary data sets describing each specific aspect of the environment in Germany. Based on the DEM, these are the slope (SLP), aspect (ASP), and topographical position index (TPI), while the Corine Land Cover (CLC) data were used to calculate a parameter for the distance of a 400 × 400 m grid cell to the nearest forest border, either when the cell was located within a forest area or outside it. The distance to nearest forest border variable (DIST) was introduced due to the exploration of the land-use data within a 400 × 400 m “landscape area” around the 56 TBEV microfoci, where only nine of them did not have any proportion of forest within their 400 × 400 m buffers (see Figure 2). The remaining 47 TBEV microfoci showed different proportions of all three CLC forest land-use classes, indicating that the presence of a forest at or nearby a TBEV microfocus might have an impact on the presence or absence of TBEV. The DIST parameter itself was derived based on the CLC data set of 2018 with an original resolution of 100 × 100 m.

Furthermore, CLC data were used to derive several landscape metrics, again using the original 100 × 100 m CLC data. It has been published before [28] that these metrics allow for the description and quantification of spatial patterns affecting ecological processes over time and space. Because of the huge number of landscape metrics that evolved during the last 25 years, a subset of commonly used metrics has been calculated and included in the investigation. These are, in detail, the number of patches (NP), the patch richness (PR), the largest patch index (LPI), the edge density (ED), the splitting index (SPLIT), and the effective mesh size (MESH). Further metrics have been calculated for the proportions of the different forest types, as well as the proportions of pastures, arable land, vineyards, and discontinuous urban fabric types. While landscape metrics are usually used to characterize a landscape based on land cover patterns within this landscape, in our investigation, the metrics were calculated for every of the 400 × 400 m grid cells used in the raster data before. The effect of landscape metrics could then be explored in the same manner as the other data set.

The data set concerning the number of notified TBEV infections was aggregated to a grand total spanning all years included in this analysis to reflect the average situation during the last 20 years, rather than in a single year. Regarding the spatial resolution, the data sets were aggregated data on county level/NUTS 3, with exceptions in regions with a huge population where smaller boroughs were used as spatial reference.

### 2.7. Analyzing the Influence of Environmental Characteristics on the Presence or Absence of TBEV and Selecting the Main Environmental Drivers for the Distribution of TBEV

We compared the characteristics of the environmental variables between 56 TBEV microfoci detected in Germany and a control or comparator set of 3575 sampling points covering Germany within a regular grid of 10 × 10 km. In order to determine which variables mattered the most for the modelling of TBEV, we applied the maximum entropy modelling algorithms from the MaxEnt software (http://biodiversityinformatics.amnh.org/open_source/maxent/ accessed on 1 April 2022). We identified a set of land cover, elevation, and meteorological variables through the literature research that were likely relevant to the distribution of TBEV in the study area. We identified those variables influencing the presence or absence of TBEV by applying Fisher’s exact test to the TBEV microfoci and the comparator points. Comparing the two distributions a Fisher’s Exact Test resulted in a *p*-value of <0.001, stating a significant difference and indicating a relationship between variables and the presence or absence of TBEV microfoci. This kind of analysis was carried out using all primary and derived variables listed above (see Table 2). Significant differences were measured using Fisher’s Exact Test. Those with *p*-values of <0.001 *** were exclusively used for the further selection of items influencing the presence or absence of TBEV microfoci in our model.

### 2.8. Selecting Main Environmental Drivers for the Distribution of TBEV by Using the MaxEnt Model

**Figure 3:**
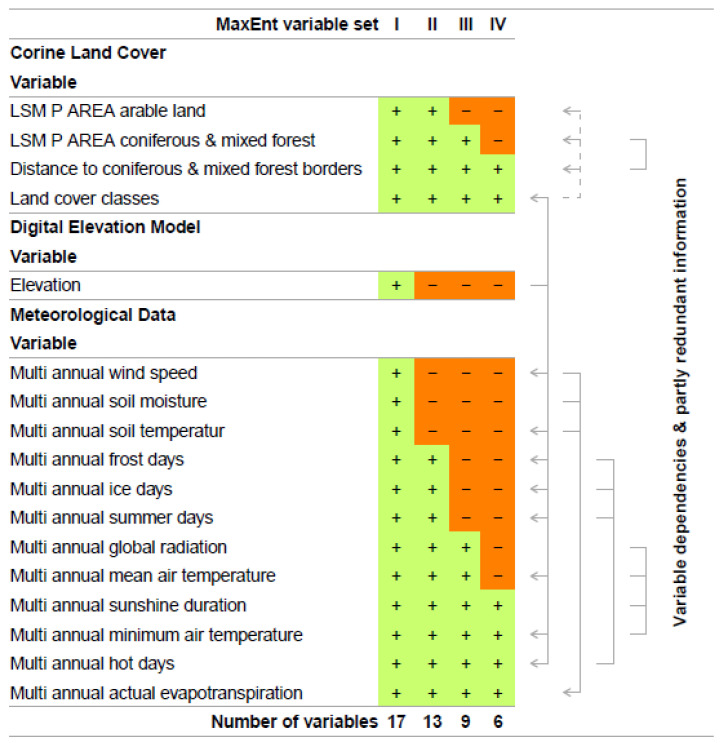
**Environmental variables that were included in the MaxEnt model**. The number of variables is step-by-step reduced to the final and most important six variables, which are used in the final run of the MaxEnt model.

Figure 3 lists those variables that passed Fisher’s Exact Test (MaxEnt variable set I). In an iterative process of reducing variable dependency and partly redundant information (MaxEnt variable set II-IV), we identified the most relevant variables for the prediction of TBEV presence. This includes the MaxEnt analysis of variable contributions, Jackknife test for variable importance, and a literature driven review of redundancy. Variable contributions were estimated as variable contributions to the model in percent and as permutation importance, where the model was reevaluated based on randomly permuted variables. Additionally, we estimated variable importance by performing a Jackknife test. Here, each variable was individually excluded from the overall model and additionally, a model with only the excluded variable was created. Therefore, the Jackknife test estimated the explanatory power of the overall model if variables were not included, as well as which variables held the highest information by themselves. This, in turn, means that variables could be dropped from the model if their contribution to the overall model was subordinate. As some of the environmental data were highly correlated (e.g., frost days and ice days), this information also influenced the decision regarding which variables were excluded from the model.

Through this iterative process and based on their individual contributions and predictive performance, we finally identified six variables that were the most effective at predicting the occurrence of TBEV (Figure 3). Those were “distance to coniferous and mixed forest borders”, “land cover classes”, “multi annual sunshine duration”, “multi annual minimum air temperature”, “multi annual hot days”, and “multi annual actual evapotranspiration”. Based on these variables, MaxEnt was used to calculate a probability distribution grid for the occurrence of TBEV with a resolution of 400 × 400 m for the whole of Germany. The results of the variable contributions and Jackknife tests for variable importance for each analytical step can be found in the Supplementary Figure S4.

### 2.9. Comparing the MaxEnt Probability Distribution and the Number of TBEV Infections in Germany

The notified incidence of TBEV infection between 2001–2020 aggregated on NUTS 3 level on the one hand, and predicted probabilities for the occurrence of TBEV in a 400 × 400 m grid by using MaxEnt, on the other hand, allowed for an additional evaluation of the MaxEnt results. This was done by aggregating the MaxEnt probabilities on the NUTS 3 level geometry and calculating the Pearson’s product–moment correlation coefficient between notified incidence and predicted probabilities based on the 401 NUTS 3 level objects for Germany.

### 2.10. Tools

Calculations and graphics were made using R version 4.0.5 (R Core Team, 2021 https://www.R-project.org/, accessed on 1 June 2022), supplemented by the packages raster (Hijmans, 2021. https://CRAN.R-project.org/package=raster, accessed on 1 June 2022), sp (Pebesma and Bivand, 2005. https://cran.r-project.org/doc/Rnews/, accessed on 1 June 2022), sf (Pebesma, 2018. https://doi.org/10.32614/RJ-2018-009, accessed on 1 June 2022), landscape metrics [29], dismo (Hijmans et al., 2021. https://CRAN.R-project.org/package=dismo, accessed on 1 June 2022), ggplot2, and others. For viewing and analysis of the gridded raster data, the open-source GIS QGIS 3.16 LTR was used.

## 3. Results

### 3.1. The Impact of Environmental Variables on the Presence or Absence of TBEV

**Figure 4:**
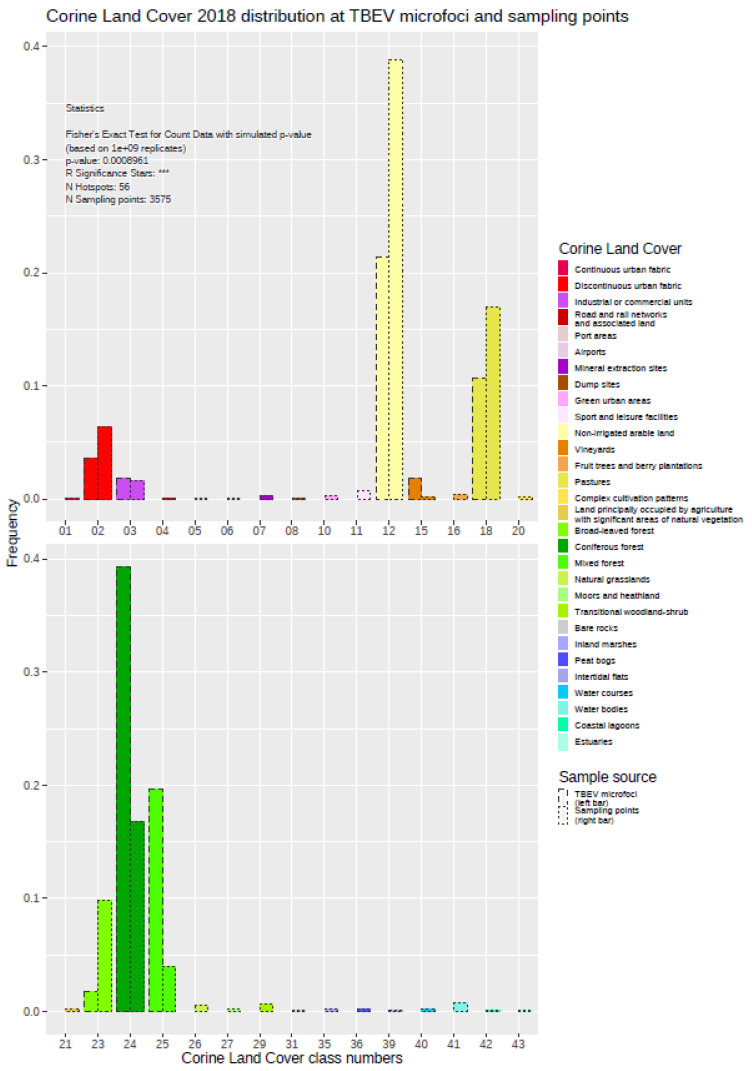
Distribution of different land cover types around the TBEV microfoci compared with the control sampling points. The results of Fisher’s Exact Test are displayed in the inset.

The exploration of the histogram plot for the Corine Land Cover data set (CLC) showed several differences between the distributions within the “landscape areas” around the TBEV microfoci compared with the control sampling points (see Figure 2 and Figure 4). In total, there were 30 different land cover types occurring at the 3575 control sampling points, while only eight different classes could be detected at the TBEV microfoci. Land-use types “vineyards” (15), “coniferous forest” (24), and “mixed forest” (25) were obviously overrepresented in the TBEV microfoci, whereas the types “discontinuous urban fabric” (02), “non-irrigated arable land” (12), “pastures” (18), and “broad-leaved forest” (23) were overrepresented at the control sampling points. The frequency of the land-use type “industrial or commercial units” (03) seemed to be more balanced.

Comparing the two distributions a Fisher’s Exact Test resulted in a *p*-value of <0.001 *** state a significant difference and indicate a relationship between land cover types and the presence or absence of TBEV microfoci. This kind of analysis was carried out using all of the primary and derived variables listed above (see Table 2a–c, Supplementary Figures S1–S3).

Variables with *p*-values of <0.001 *** were then exclusively used for the further analysis concerning the influence of environmental factors on the presence or absence of TBEV microfoci in particular. In this context, the most important characteristics were ground elevation (see Table 2b); most of the climatological variables (see Table 2c); and, regarding landscape metrics, the proportion of coniferous and mixed forest, the proportion of arable land, and the distance to the nearest coniferous and mixed forest border (see Table 2a). Slope and aspect derived from the Digital Elevation Model or most of the other landscape metrics did not show a significant difference in their distributions. The topographical position index had weak significance at *p* = 0.03 *.

With regard to landscape fragmentation in/around TBEV microfoci, there were several characteristics with a weaker statistical significance, such as edge density (ED), number of patches (NP), and splitting index (SPLIT). These items indicated that in/around TBEV microfoci, the landscape was more fragmented (see Table 1). However, these characteristics were not included in the planned MaxEnt model, because other variables seemed to have a geo-statistically greater impact on the presence or absence of TBEV microfoci in nature.

This was particularly true for 12 of our climatological variables showing a significance of *p* < 0.001 ***. Because of their cross correlations and mutual influences, some of them tended to explain similar characteristics in the presence or absence of TBEV microfoci, which had to be considered when interpreting which of the climatological variables mattered the most. Multi annual actual evapotranspiration, for example, highly depends on multi-annual wind speed, multi annual soil moisture, and multi annual soil temperature, and thus acts as one of the most important variables where the wind and soil variables can be omitted. A similar statistical coincidence is given by the variables multi-annual frost days, multi-annual ice days, multi-annual summer days, and multi-annual hot days on the one side, and multi-annual global radiation, multi-annual mean temperature, multi-annual sunshine duration, and multi-annual minimum temperature on the other side. At best, we found the four climatological variables of multi-annual sunshine duration, multi-annual minimum temperature, multi-annual hot days, and multi-annual actual evapotranspiration, which mainly control the presence or absence of TBEV microfoci concerning climatological conditions (Figure 3).

### 3.2. MaxEnt Prediction Model

**Figure 5:**
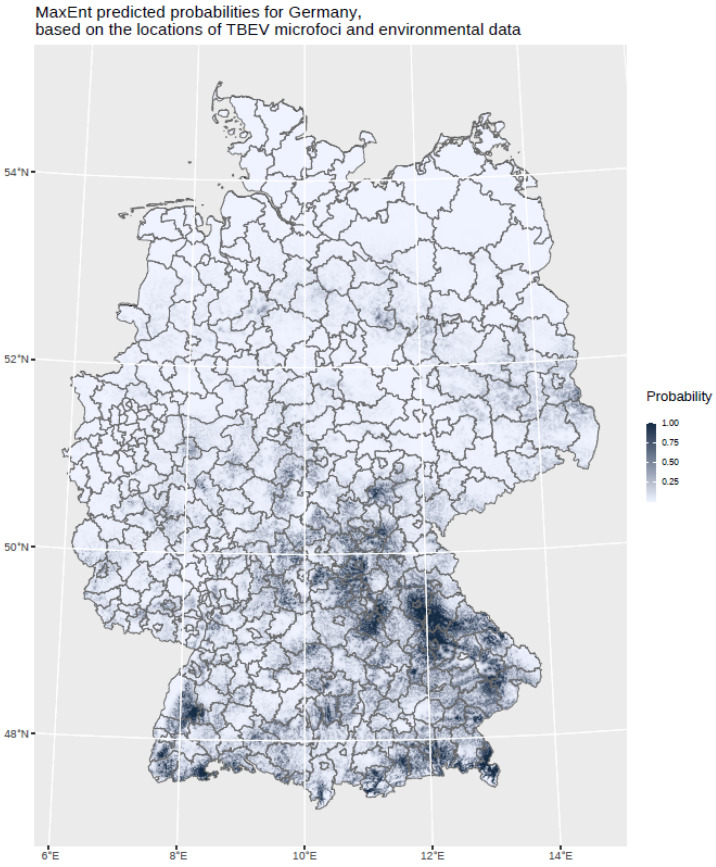
MaxEnt predicted probabilities for Germany based on the locations of the TBEV microfoci and environmental data. The probability is displayed in different grey shades.

**Figure 6:**
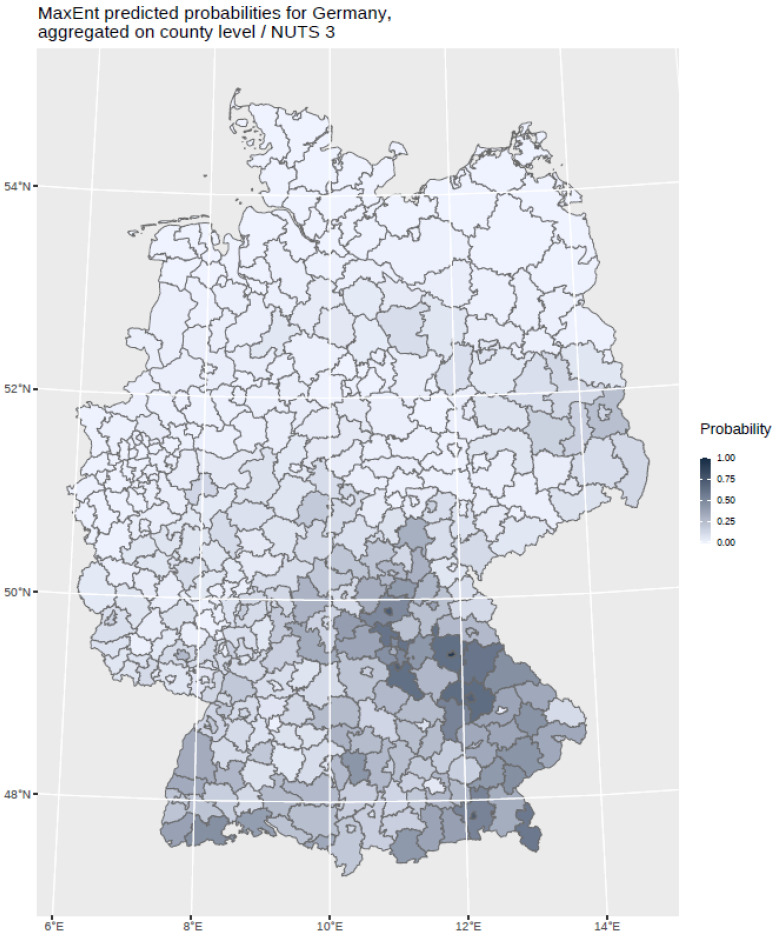
MaxEnt predicted probabilities for Germany aggregated on NUTS 3 level. The probability is displayed in different grey shades.

**Figure 7:**
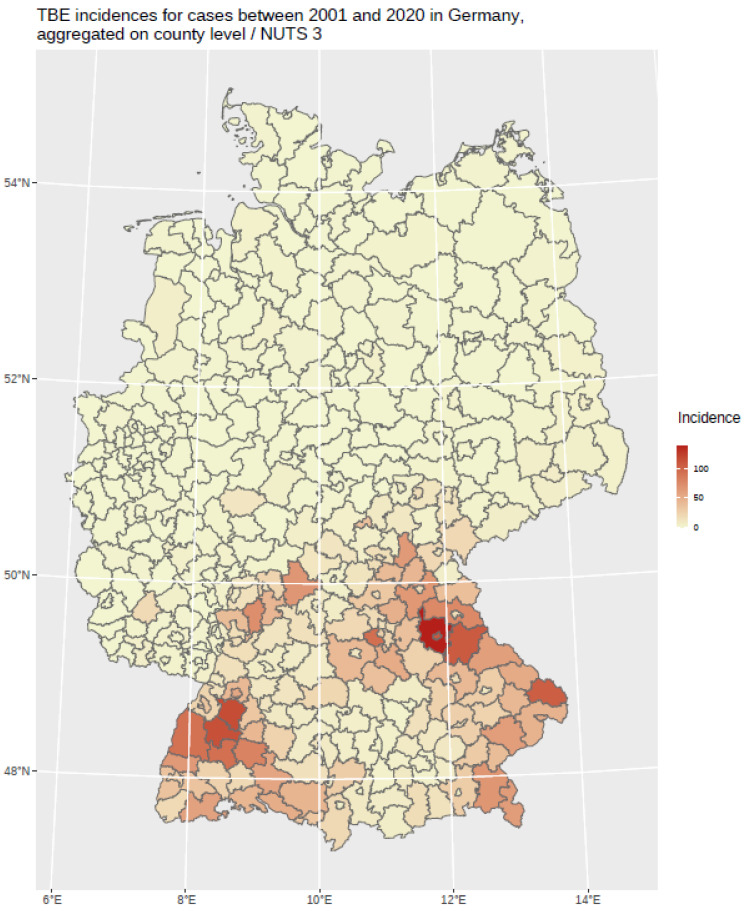
TBE incidence between 2001 and 2020 in Germany based on notified TBEV infections, aggregated on NUTS 3 level. The incidence is displayed in different red shades.

**Figure 8:**
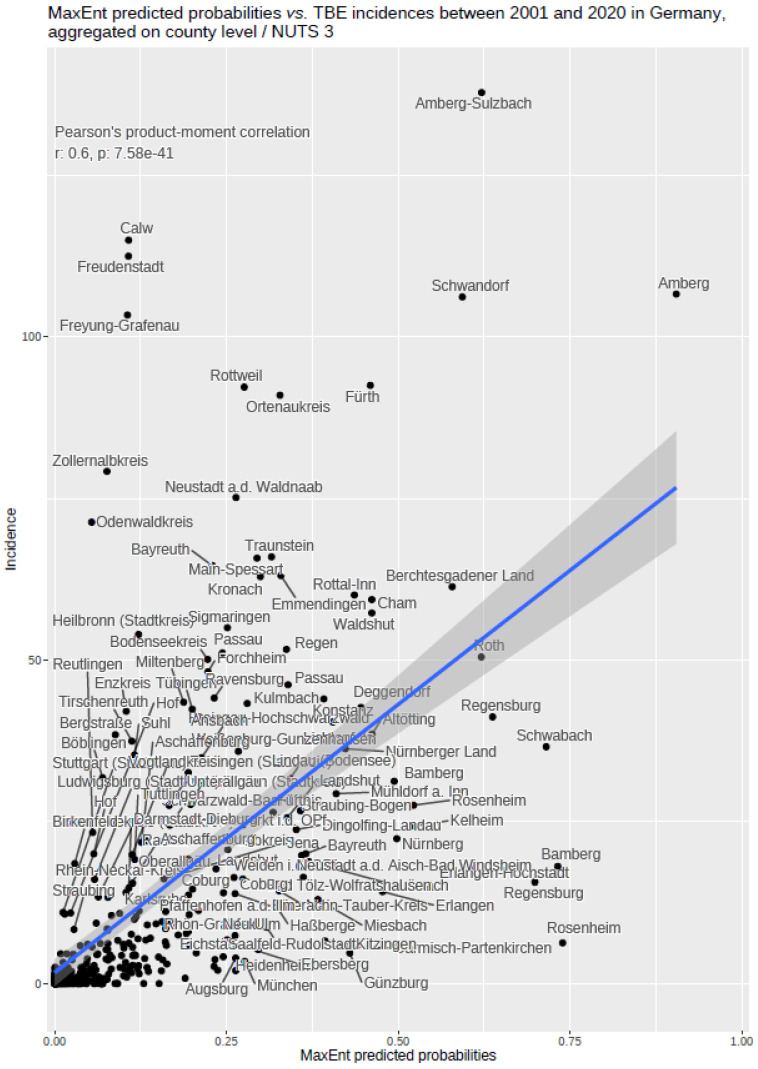
MaxEnt predicted probabilities correlated with TBE incidences between 2001 and 2020 in Germany, data are aggregated on NUTS 3 level. The inset shows the results of Pearson’s product–moment correlation.

The findings indicate that multi-annual actual evapotranspiration (27.0%) and multi-annual hot days (22.5%) had the most explanatory power in our model. These two variables were followed by four others with a lower, but still important, explanatory power: land cover classes (19.6%), multi-annual minimum air temperature (14.9%), multi-annual sunshine duration (9.0%), and distance to coniferous and mixed forest border (7.0%). Based on this selected set of the statistically most powerful six environmental parameters (see Table 2), a MaxEnt model was used to calculate a probability distribution grid for the occurrence of TBEV with a resolution of 400 × 400 m, as illustrated in Figure 5. The predicted probability was visualized by different shades. It was demonstrated that the highest prediction probability for the presence of TBEV was calculated in the federal states of Bavaria and Baden-Wuerttemberg, with a particular focus on regions of the Black Forest, the Bavarian Forest, and Upper Bavaria with its submontane districts. To a lower extent, areas of southern Hesse, Thuringia, Saxony, and Rhineland-Palatinate, as well as Saarland, were affected. There was a clear North-South divide. Interestingly, the MaxEnt model also displayed the northern German regions, with considerable probability for the presence of TBEV in the environment. This particular geographic area spans from Saxony to the most western parts of the federal state of Lower Saxony. In a further step, the results were aggregated on NUTS level 3, see Figure 6, for correlation with national notification data on TBEV, which resembled a statistically independent set of data. MaxEnt predicted probabilities and the notified incidence of TBEV infection between 2001–2020 (see Figure 7) were correlated using Pearson’s product–moment correlation coefficient (see Figure 8). The correlation with the national notification dataset showed a strong significance with *p* < 0.001 *** and r at 0.60.

## 4. Discussion

It is of great scientific importance to understand zoonotic infections and to identify the core environmental factors maintaining the zoonotic cycles of the pathogens in the biocenosis and in the transmission to humans. We introduce a MaxEnt model for the detection of TBEV microfoci in Germany. The machine-learning-based approach, called maximum entropy modeling, allowed for the calculation of a probability distribution resulting in a fine-grained prediction map (400 × 400 m). This spatial resolution approximates the biological concept of the microfoci embedded in larger natural foci very closely [21,24]. It is speculated that the microfocus size of approximately 1ha is defined by the presence of the vector, mainly *Ixodes ricinus*, the reservoir host, e.g., small rodents, and its biological operating range in such a habitat.

From a vector biological point of view, the review of variables influencing the presence or absence of TBEV is straightforward. Multi annual actual evapotranspiration, as well as multi annual hot days, multi annual minimum air temperatures, and multi annual sunshine duration or very similar temperatures indices, are linked in the literature to nymph activity [8]. There is a well characterized complex interplay of TBEV vector dynamics and TBEV infection rates in ticks [8,30]—notably, evapotranspiration is of vital importance for *I. ricinus*, because of its known high sensitivity to aridity. The presence of coniferous or mixed forests and the distance to coniferous and mixed forest borders were identified as further important factors. These findings call attention to the ecological niche of the ecotone, which has been of particular interest for investigating TBEV microfoci ever since. Most microfoci are located at such forest thresholds, displaying a unique dynamic of *I. ricinus* and small mammals between the ecotone and areas just behind this threshold in the forests (pers. observation G. Dobler). The balance of this ecotone system is highly sensitive to changes in the meteorological framework [31].

Although not included into our MaxEnt model because of technical reasons, a closer look at the landscape metrics characterizing the fragmentation of the landscape is worthwhile. With a weaker significance than the six selected variables for the MaxEnt model, these items seem to have some impact on the probability for the presence of a TBEV microfocus in nature. The degree of fragmentation and its influence remains a matter of debate, without definitive answers. There might be links between fragmentation and TBEV microfocus presence. A repeatedly stated controversial hypothesis for the reemergence of TBEV in the eastern federal states of Germany—former GDR—might be the changes in landscape fragmentation. The GDR propagated and state driven agricultural system was organized in agricultural collectives, which cultivated huge patches of arable land. After the breakdown of the collective agricultural system in the 1990s, the arable land became more fragmented due to privatization. Simultaneously, there was a reemergence of TBEV in Saxony and Thuringia. However, at this stage, our data do not support this thesis—on the contrary, our findings point at an increased mesh size and less fragmentation around the TBEV microfoci. This could be due to reafforestation measures after 1990. In brief, the issue of TBEV reemergence in the eastern federal states of Germany should be subject to further geostatistical research.

Our current model has practical implications because it enables public health care authorities to anticipate where new TBEV microfoci and potential consecutive infections will appear (or disappear). The discovery of new TBEV microfoci in Saxony (*unpublished observation M. Pfeffer*) is in line with our prediction model, displaying an east-to-west probability for the presence of TBEV microfoci in north/middle Germany [32]. Other newly identified TBEV microfoci in Lower Saxony, around Hannover, which were not included in our MaxEnt model, are located in the predicted east-to-west band as well [32]. In this context, it should be a matter of debate whether the current risk classification of the RKI, based on notification data, should be revised or complemented by our results, which are derived from vector biological factors/findings. Overall, our data are consistent with findings from a recently published work, which used the self-reported points (*n* = 567) of potential infection (POI) and a smaller amount of known microfoci (*n* = 41) for analyses and an ecological niche model [19], exclusively regarding the high-endemic federal states of Bavaria and Baden-Wuerttemberg. To determine the characteristics of the POIs, comparator points/polygons were generated. The self-reported polygons of POI were 2.23 km^2^, which are quite large area patches given the hypothesis that TBEV microfoci were sized at approximal 1ha. A strength of our study was the fine-grained resolution of 400 × 400 m, which enabled detailed spatial statements. Previous analyses used 10 × 10 km resolutions at a subdistrict level for the illustration of potential POIs [19]. Furthermore, our findings are based on defined TBEV microfoci with known histories of infection and the repeated confirmation of the virus during the last years, resulting in an in-depth forecasting model of TBEV microfoci. Biotic factors such as local tick abundance or anthropogenic factors such as vaccination coverage have not been directly included in our investigation. This might be a limitation of our study. However, we primarily identified the natural transmission foci of TBEV, where humans do not play a biological role. Nevertheless, comparing the MaxEnt prediction data (aggregated on NUTS 3 level) with the notification dataset spanning the years 2001–2021, we can see a good correlation. This correlation with a nearly independent set of data underscores the quality of the established MaxEnt model.

## 5. Conclusions

Our findings are based on defined TBEV microfoci with known histories of infection and the repeated confirmation of the virus in the last years, resulting in an in-depth high-resolution model/map of TBEV microfoci in Germany. Multi annual actual evapotranspiration (27%) and multi annual hot days (22.5%) have the most explanatory power in our model. The results may be used to tailor specific regional preventive measures and investigations.

## Figures and Tables

**Table 1:** **TBEV microfoci included in our analysis**. For each TBEV microfocus, geodata are referenced as well as the corresponding federal states.

NAME	FEDERAL STATE	GEODATA N	GEODATA E
**SCHILTACH**	BW	48.293264	8.320032
**AUBACHSTRASSE**	BW	48.638261	8.124403
**ZELL A.H.**	BW	48.337588	8.069234
**OBERRIED**	BW	47.955061	7.961001
**OEDENREUTH**	BAY	49.382107	10.89899
**HOEHENGAU**	BAY	49.500923	11.859877
**BURGLENGENFELD**	BAY	49.187749	12.037393
**NEUHOF**	BAY	49.114536	11.878744
**ASCHACH**	BAY	49.468414	11.884132
**POPPENRICHT**	BAY	49.478247	11.789021
**JAEGERSHOF**	BAY	49.299229	13.026569
**BUCHTAL**	BAY	49.404872	12.081410
**ELSENTHAL**	BAY	48.840594	13.384545
**RUIT**	BAY	49.399371	12.127877
**FUERSTENSTEIN**	BAY	48.716547	13.316138
**HASELMUEHL**	BAY	49.408911	11.882931
**HESELBACH**	BAY	49.297312	12.200458
**IMMENSTETTEN I**	BAY	49.499808	11.889831
**IMMENSTETTEN II**	BAY	49.482915	11.885282
**KOELBLDORF**	BAY	49.260300	12.247922
**WACKERSDORF**	BAY	49.320899	12.204749
**WOLFSBACH**	BAY	49.361010	11.911637
**PENKHOF**	BAY	49.411895	11.921286
**MAIS**	BAY	47.982107	12.592499
**LEUPOLZ**	BW	47.752795	9.817677
**AAAASTEINBERG**	BAY	49.275271	12.176248
**MUEHLAU**	BAY	47.724978	12.392581
**PETTING**	BAY	47.926210	12.819270
**BATTAUNE**	SAC	51.599236	12.751076
**HUB**	BAY	49.244607	11.961373
**ELSTERBERG**	THUE	50.611252	12.155112
**EBERMANNSDORF**	BAY	49.395530	11.945491
**SPIESSBERG**	BW	47.699637	9.737666
**FEUERHOF**	BAY	49.522681	11.746832
**RHEINE**	NRW	32.233590	7.527555
**LINGEN**	NS	52.505504	7.275217
**HERRNRIED**	BAY	49.103862	11.735617
**AMOENAU**	HES	50.898691	8.689737
**FRAUNBERG**	BAY	49.468676	12.138802
**WANGEN I**	BW	47.699354	9.816652
**WANGEN II**	BW	47.697346	9.798715
**SCHNAITTENBACH**	BAY	49.529464	11.993255
**GLEISSENBACH**	BAY	48.469833	12.062751
**MUENCHEN-PERLACH**	BAY	48.079474	11.595674
**TUEBINGEN**	BW	48.548510	9.060509
**WAGING**	BAY	47.926538	12.736940
**KARSEE**	BW	47.746614	9.806434
**NUERNBERG**	BAY	49.429985	11.142228
**WETTER**	HES	50.907811	8.750076
**RAFFA**	BAY	49.198897	12.077333
**INZENDORF**	BAY	49.449152	12.094763
**URSULAPOPPENRICHT**	BAY	49.498136	11.859962
**ASBACH**	BAY	49.381200	12.169548
**WEINBERG**	BAY	49.326259	12.129508
**ZWIEFALTEN**	BW	48.247522	9.460531
**HAHNBACH**	BAY	49.498350	11.859444

Abbreviations Federal States: BAY: Bavaria; BW: Baden-Wuerttemberg; HES: Hesse; NRS: Northrhine-Westfalia; NS: Lower Saxony; SAC: Saxonia; THUE: Thuringia.

**Table 2:** (a–d) All initially included and tested environmental variables (landscape metrics, digital elevation model, and meteorological variables). *P* values are displayed. For further details regarding the environmental variables and a description for an analysis using R, we refer to https://r-spatialecology.github.io/landscapemetrics/, accessed on 1 April 2022.

**(a)**	
**LANDSCAPE METRIC (LSM)**	*p* value
**L ED**	0.009 **
**L SHAPE MN**	0.153
**L LSI**	0.009 **
**L AREA MN**	0.023 *
**L NP**	0.015 *
**L CORE SD**	0.721
**L SHEI**	0.204
**L SHDI**	0.024 *
**L SIDI**	0.023 *
**L SIEI**	0.173
**L FRAC MN** **L PARA MN**	0.124 0.013 *
**L PR**	0.063
**L PD**	0.015 *
**L LPI**	0.007 **
**L CONTIG MN** **L CONTAG**	0.064 0.341
**L IJI**	0.313
**L COHESION**	0.021 *
**L MESH**	0.017 *
**L SPLIT**	0.006 **
**L AI**	0.023 *
**L ENT**	0.012 *
**L DCAD**	0.015 *
**L TCA**	0.041 *
**L JOINENT**	0.003 **
**P AREA DISCONTINUOUS URBAN FABRIC**	0.58
**P AREA INDUSTRIAL OR COMMERCIAL UNITS**	0.500
**P AREA ARABLE LAND**	<0.001 ***
**P AREA VINEYARDS**	0.031 *
**P AREA PASTURES**	0.393
**P AREA BROAD LEAVED FOREST**	0.203
**P AREA CONIFEROUS FOREST**	<0.001 ***
**P AREA MIXED FOREST** **P AREA CONIFEROUS AND MIXED FOREST**	<0.001 *** <0.001 ***
**DISTANCE TO FOREST BORDER**	0.444
**DISTANCE TO BROAD LEAFED FOREST BORDER**	0.036 *
**DISTANCE TO CONIFEROUS FOREST BORDER**	0.043 *
**DISTANCE TO MIXED FOREST BORDER**	0.002 **
**DISTANCE TO CONIFEROUS AND MIXED FOREST BORDER**	<0.001 ***
**DISTANCE TO ARABLE LAND BORDER**	0.431
**(** **b)**	
**DIGITAL ELEVATION MODEL**	*p* value
**ELEVATION**	<0.001 ***
**TPI**	0.032 *
**ASPECT**	0.323
**SLOPE**	0.547
**(** **c)**	
**METEOROLOGICAL VARIABLES**	*p* value
**MATEMP** **MASUMMERTEMP**	<0.001 *** 0.108
**MATEMPMIN**	<0.001 ***
**MATEMPMAX**	0.199
**MAPRECIP** **MASUMMERPRECIP**	0.002 ** 0.006 **
**MAVEGBEG**	0.005 **
**MAWDAT**	<0.001 ***
**MASUN**	<0.001 ***
**MAFROSTD**	<0.001 ***
**MAICED** **MASUMMERD**	<0.001 *** <0.001 ***
**MAHOTD**	<0.001 ***
**MAEVAR**	<0.001 ***
**MASOILM**	<0.001 ***
**MASOILTEMP** **MAGRAD**	<0.001 *** <0.001 ***
**(** **d)**	
**POPULATION**	*p* value
**POP**	0.363

See for further details: https://r-spatialecology.github.io/landscapemetrics/ accessed on 1 June 2022. * *p* < 0.05; ** *p* < 0.01; *** *p* < 0.001.

## Data Availability

The datasets used and/or analyzed during the current study are available from the corresponding author on reasonable request and/or are open access available.

## Supplementary Materials

The following supporting information can be downloaded at: https://www.mdpi.com/article/10.3390/ijerph191811830/s1.
