# Supplementary material for: Decoding the Geography of Natural TBEV Microfoci in Germany: A Geostatistical Approach Based on Land-Use Patterns and Climatological Conditions
Source: Int J Environ Res Public Health. 2022 Sep 19;19(18):11830. doi: 10.3390/ijerph191811830 (PMC9517139; doi:10.3390/ijerph191811830)
Supplement: Supplementary file 1 [file ijerph-19-11830-s001.zip › Suppl 1. Material and methods.pdf]

Suppl. 1 Material and methods

Tick collection

Ticks were collected by flagging, as described before. Ticks were kept alive and morphologically identified at the TBEV national reference laboratory to species level (as previously published [20]). Ticks were pooled according to developmental stage and sex (10 nymphs and 5 adult female or male ticks) and processed for TBEV RNA and virus isolation according to Kupca et al. [21], except that for virus isolation A549 cells were used instead of Vero cells. There were no specific permissions required for field research. No endangered or protected species were sampled or disturbed during the sampling activities. All work was done in cooperation with the local public health authorities and in-line with federal legislation.

TBEV detection and TBEV isolation

The ticks were pooled in Lysing Matrix A tubes (MP Biomedicals, Eschwege, Germany) with 1 ml media (Minimal Essential Medium, 3% Fetal Calf Serum, 10fold antibiotics and antimycotics, Invitrogen, Karlsruhe, Germany) and subsequently homogenized using the MP Tissue Lyser (MP, Eschwege, Germany). Total nucleic acid extraction was performed using MagNA Pure LC Total Nucleic Acid Isolation Kit (Roche, Mannheim, Germany) and the automated isolation and purification instrument MagNa Pure LC (Roche, Mannheim, Germany) according to the manufacturer's instructions. Isolated nucleic acid was eluted in 50 µl volume and stored at -80°C until further analysis. The samples were screened for TBEV RNA using RT-PCR [22]. Furthermore, PCR-positive results were confirmed by amplification of the viral E gene. The E gene amplicons were purified and isolated using the QIAquick Gel Extraction and QIAquick PCR Purification Kits (Qiagen, Hamburg, Germany); E gene products and specific primers (primer details have been published [23] open

access) were sent out for external sequencing provided by GATC Biotech (Konstanz, Germany).

Supernatants of positive tick pools were used for virus isolation. 0.5 ml of the supernatant was diluted 1:5 and 1:25 in the lysing medium and A549 cells were inoculated for one hour at 37°C. The inoculum was removed and the cells washed with medium 3 times. Then 5 ml of cell culture medium (identical to medium as described plus 3% fetal calf serum) were added to each tube and the cells were incubated at 37°C, 5% CO_2 for 7 days. Cell culture supernatants were tested for TBEV by RT-qPCR and from 2009 to 2016 were used for amplification of E gene for sequencing. From 2016 on a technique was used to sequence the E gene directly from the lysed tick supernatant without isolation of the TBEV strain. Comparative studies showed that there were no changes of the nucleotide or amino acid sequences of the E genes directly sequenced from ticks or from TBEV isolates (personal communication Gerhard Dobler). Ticks were only classified as positive if PCR was confirmed either by virus isolation or by generation of a positive E gene gene sequence. DNA sequence analysis, generation of phylogenetic data and trees were analyzed as published previously [23].
