# Supplementary figures and images for: Decoding the Geography of Natural TBEV Microfoci in Germany: A Geostatistical Approach Based on Land-Use Patterns and Climatological Conditions
Source: Int J Environ Res Public Health. 2022 Sep 19;19(18):11830. doi: 10.3390/ijerph191811830 (PMC9517139; doi:10.3390/ijerph191811830)

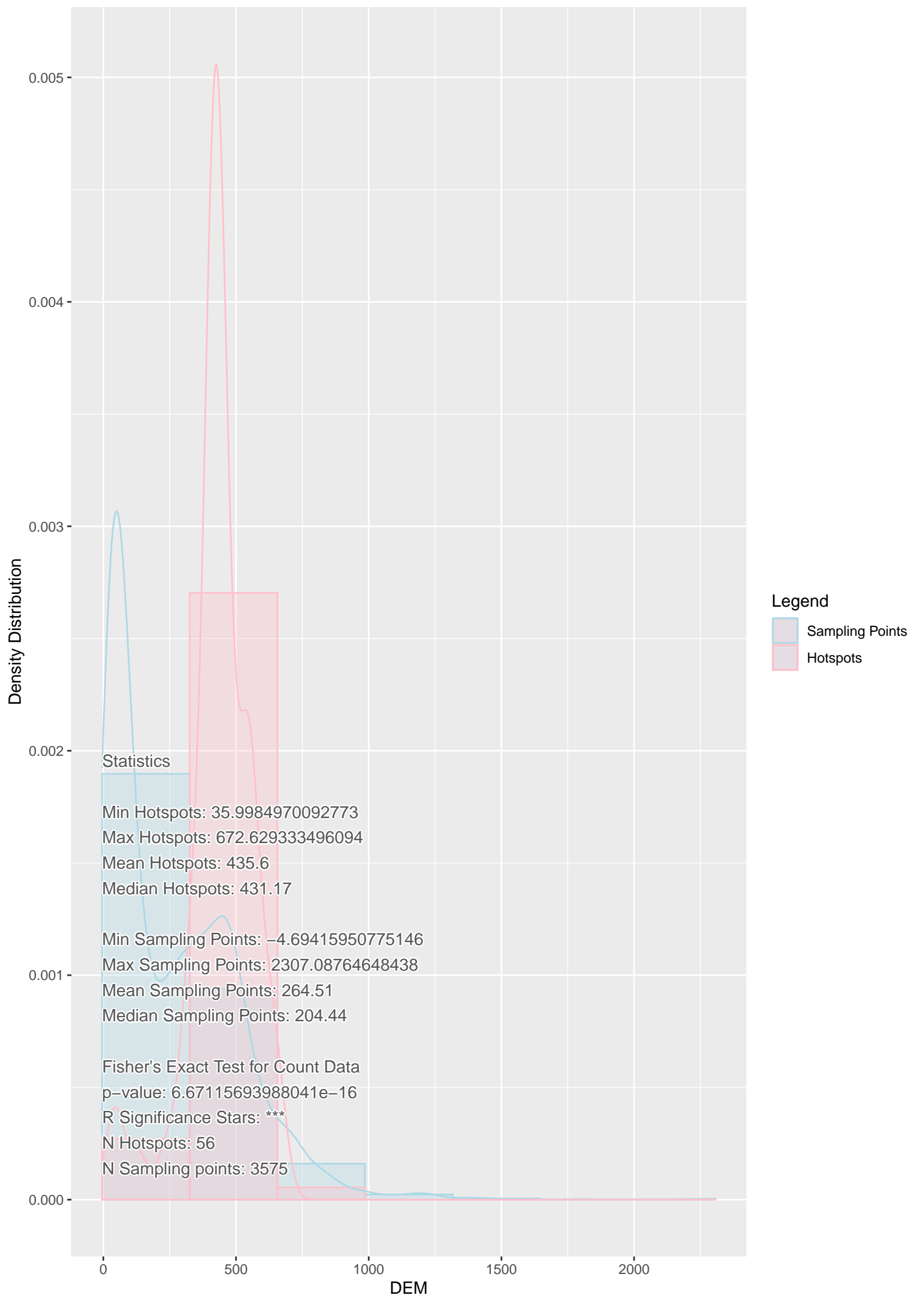

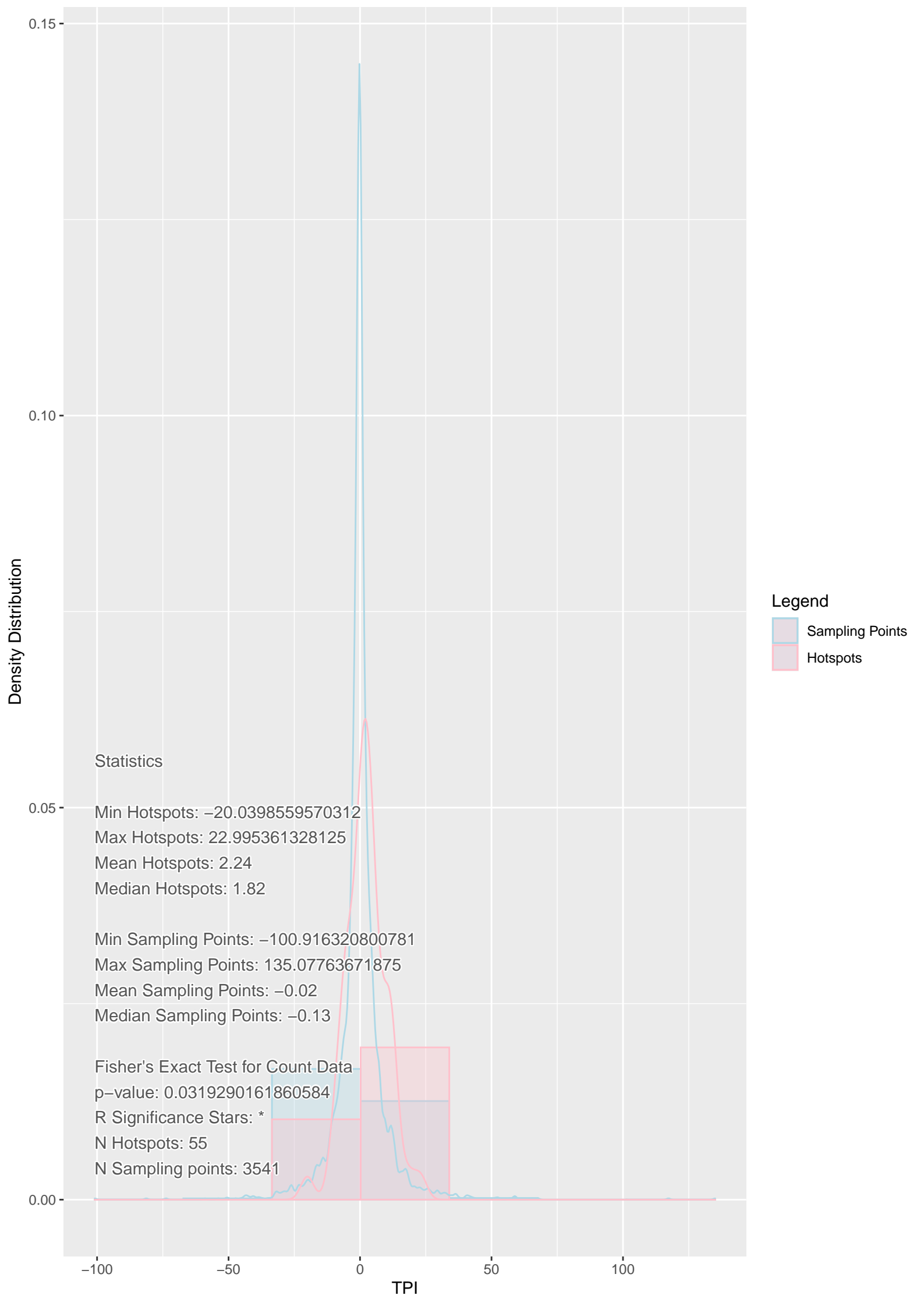

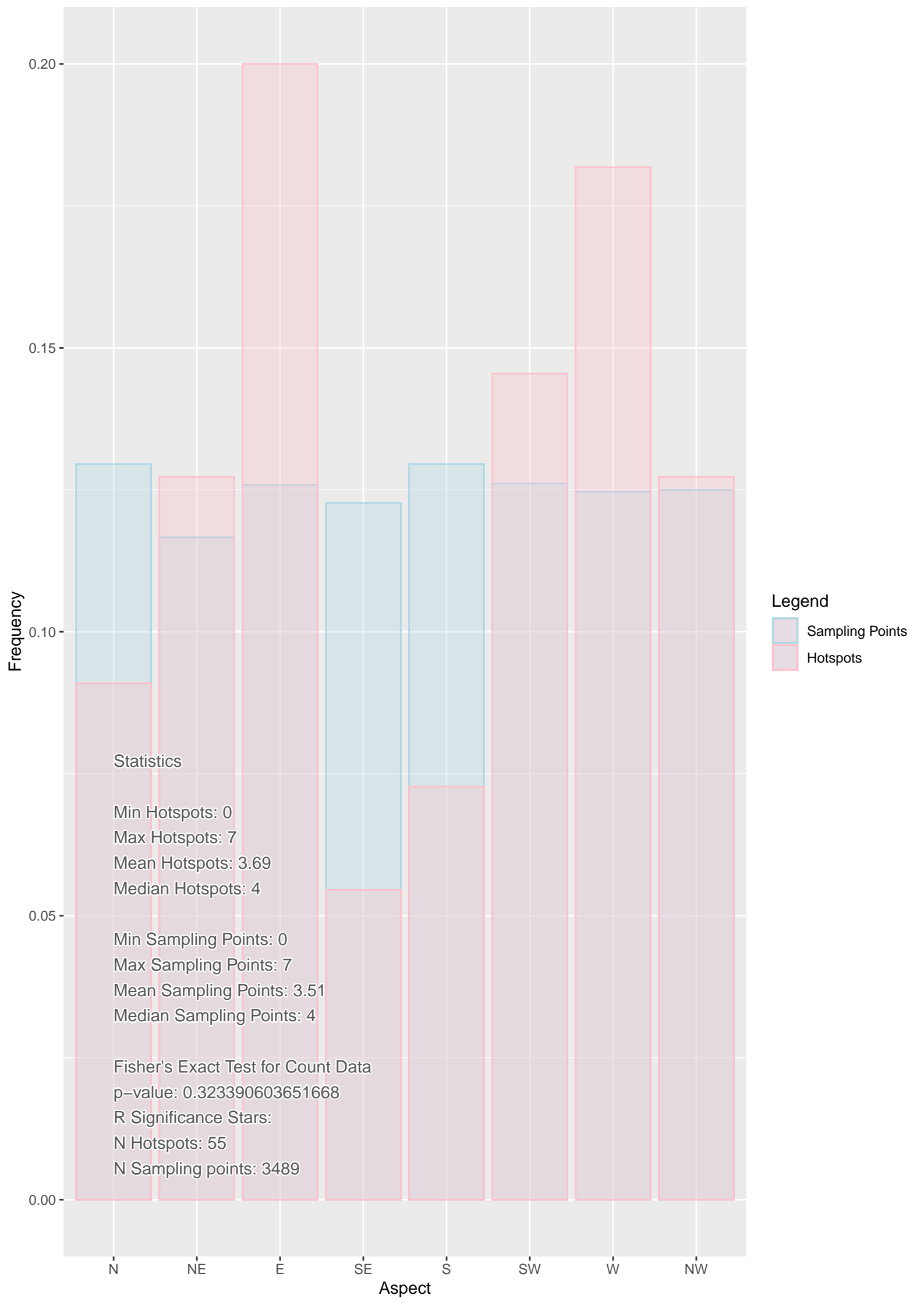

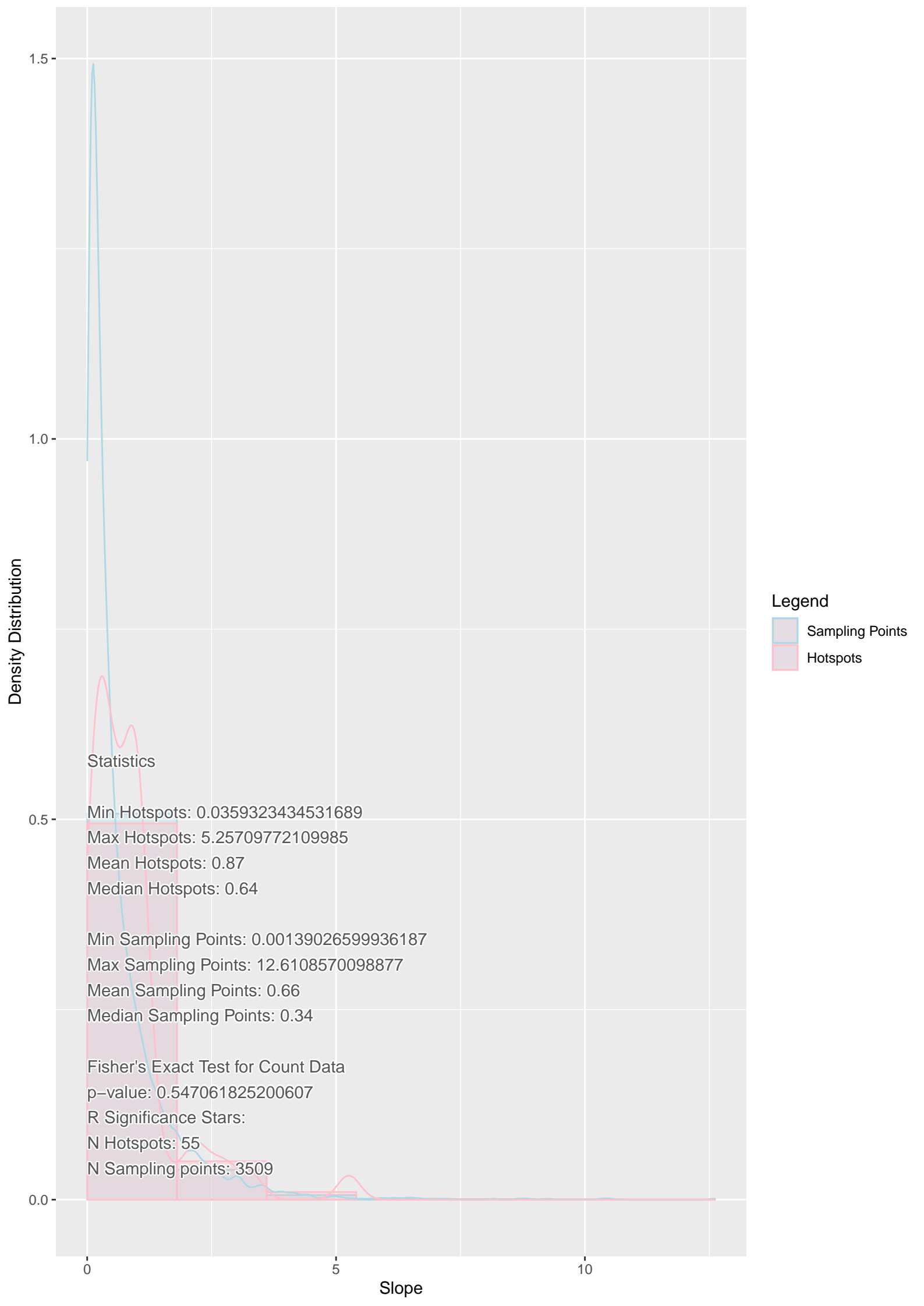

Supplement: Supplementary file 1 [file ijerph-19-11830-s001.zip › suppl. fig 1.pdf]

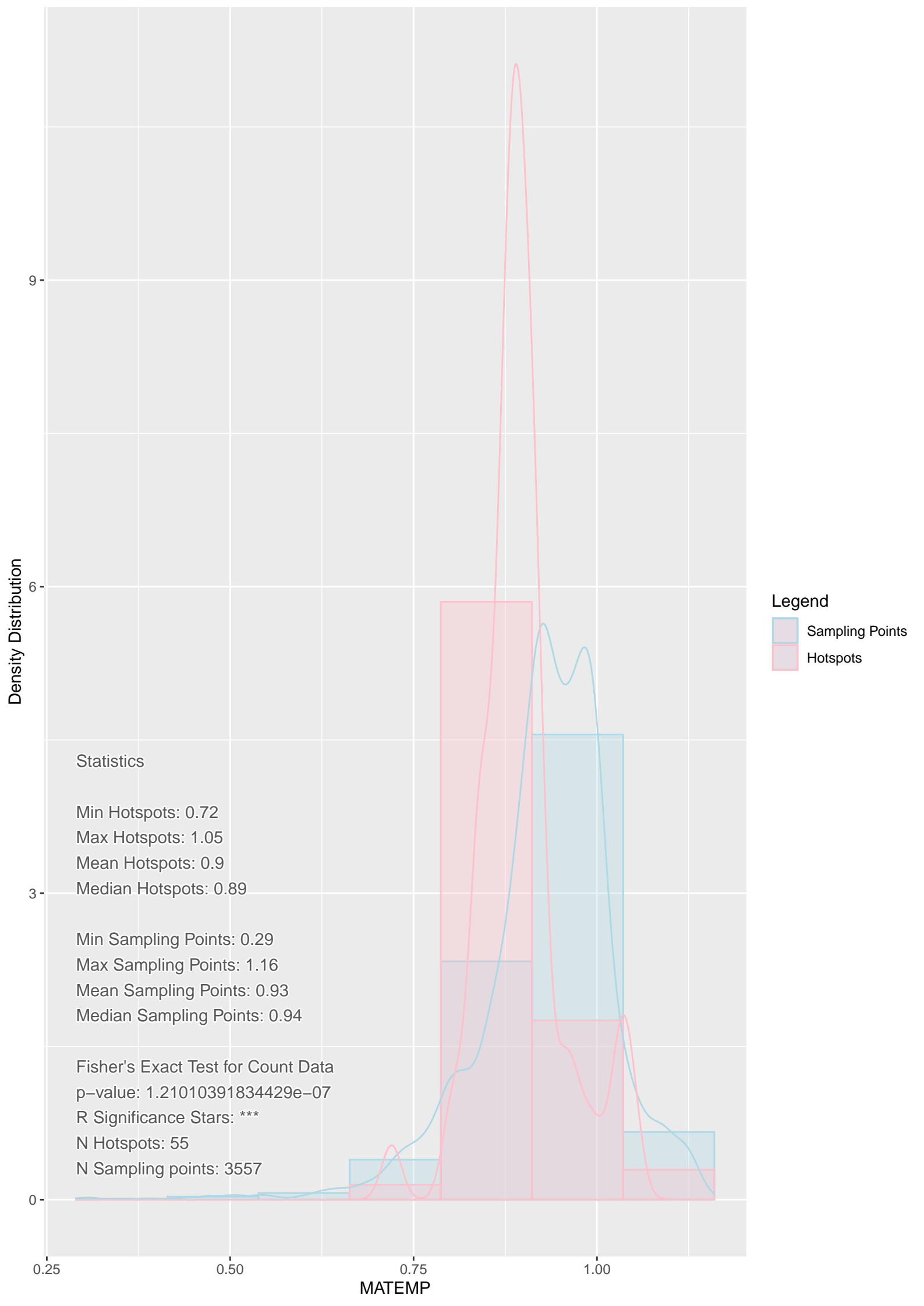

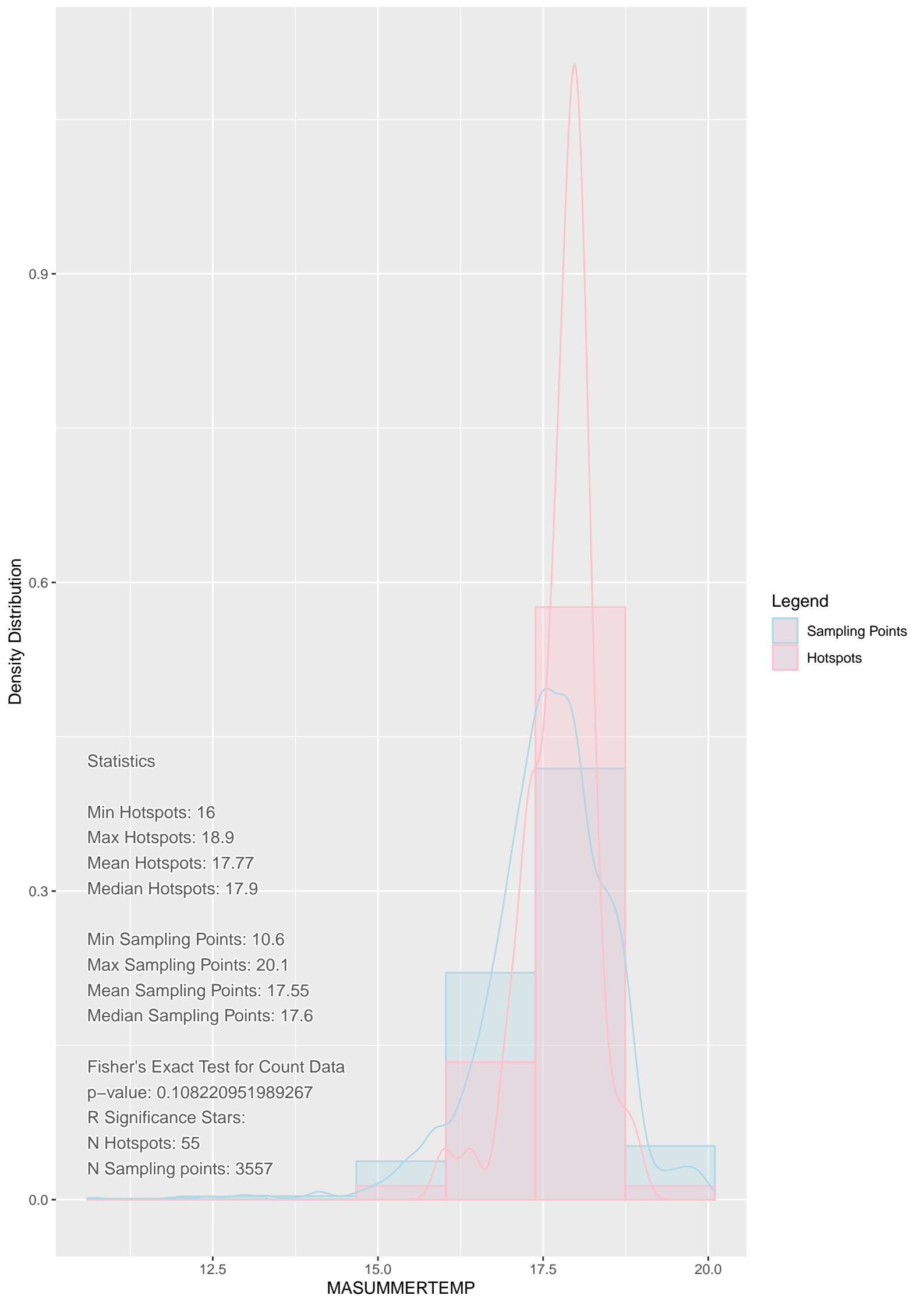

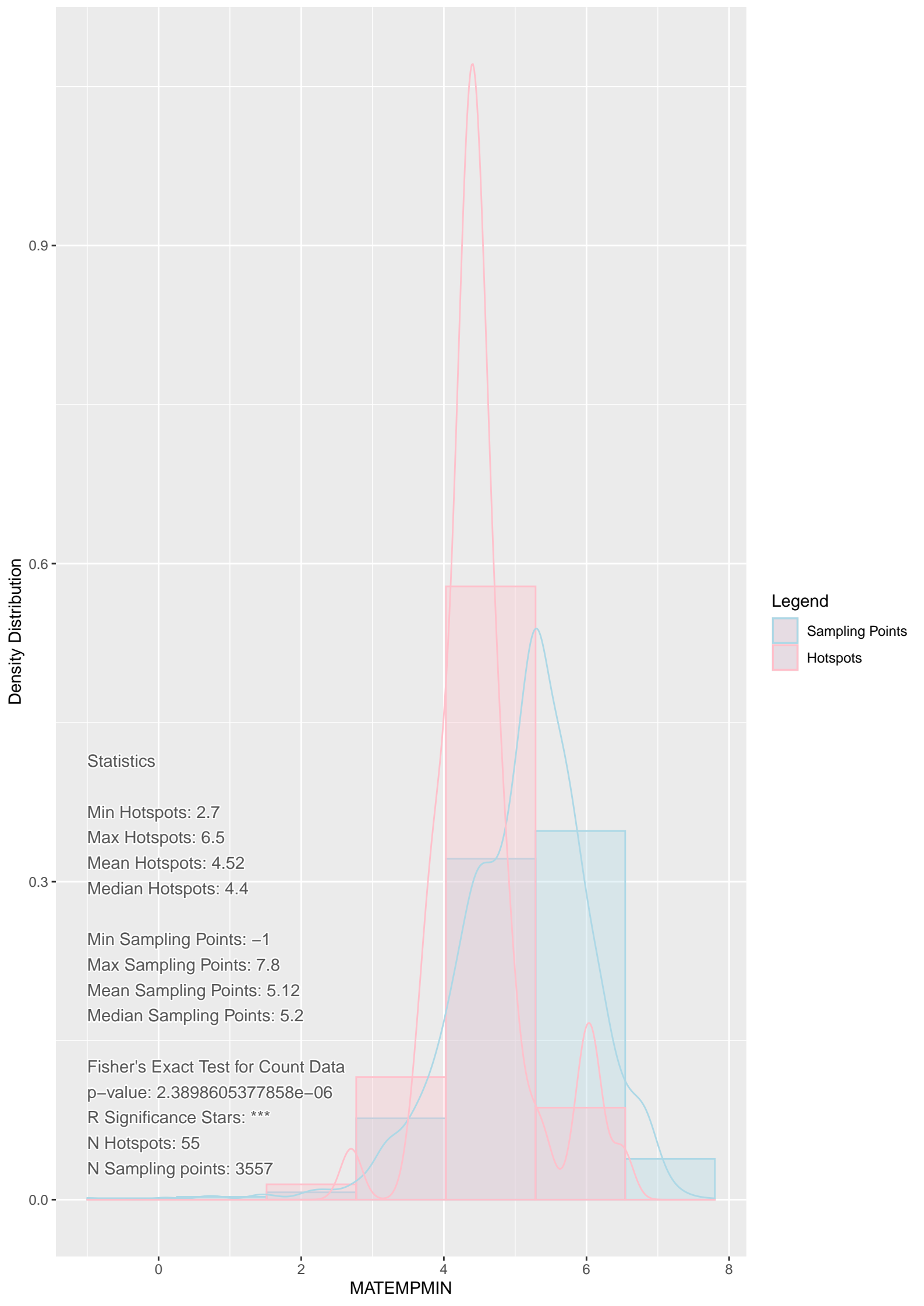

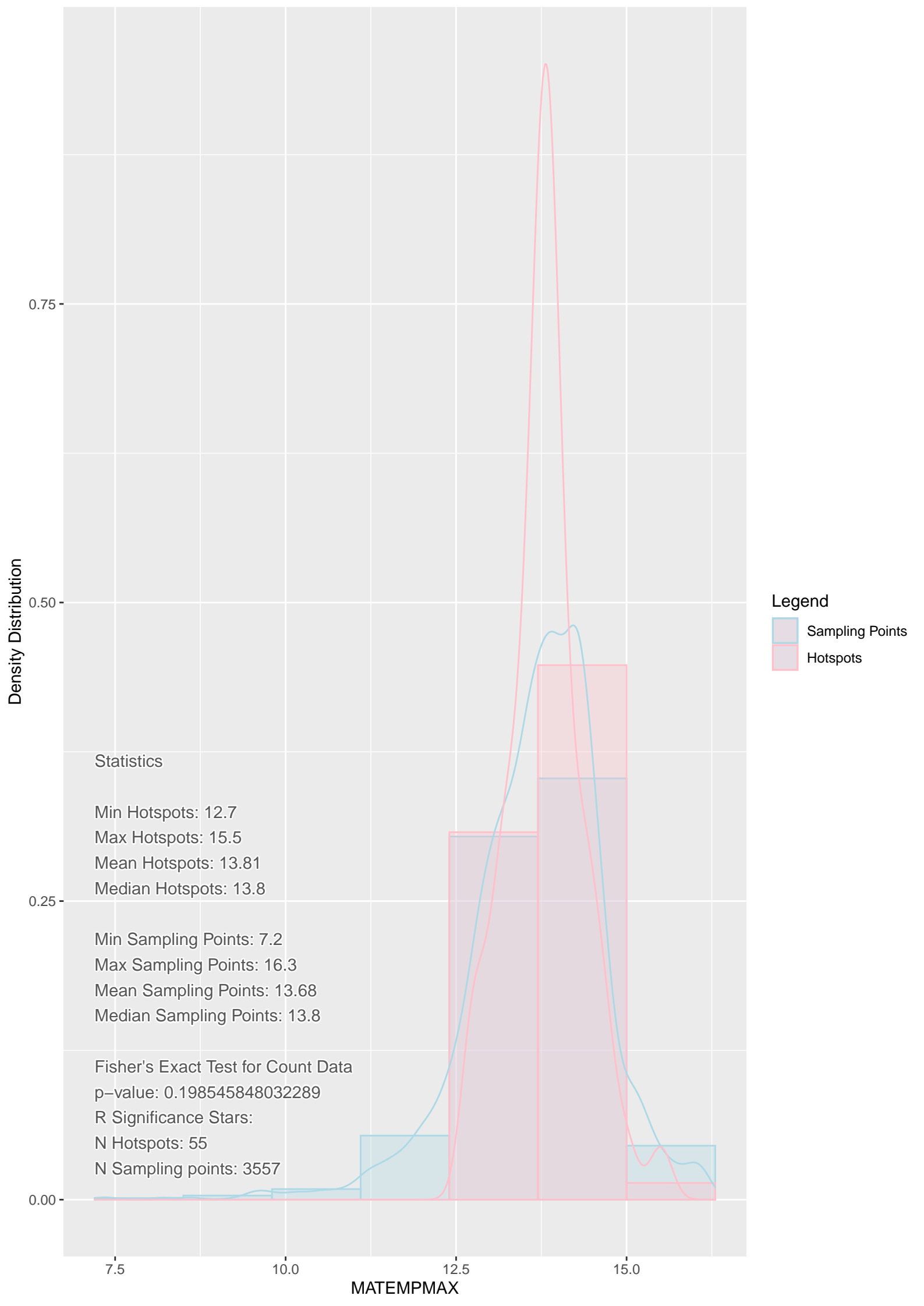

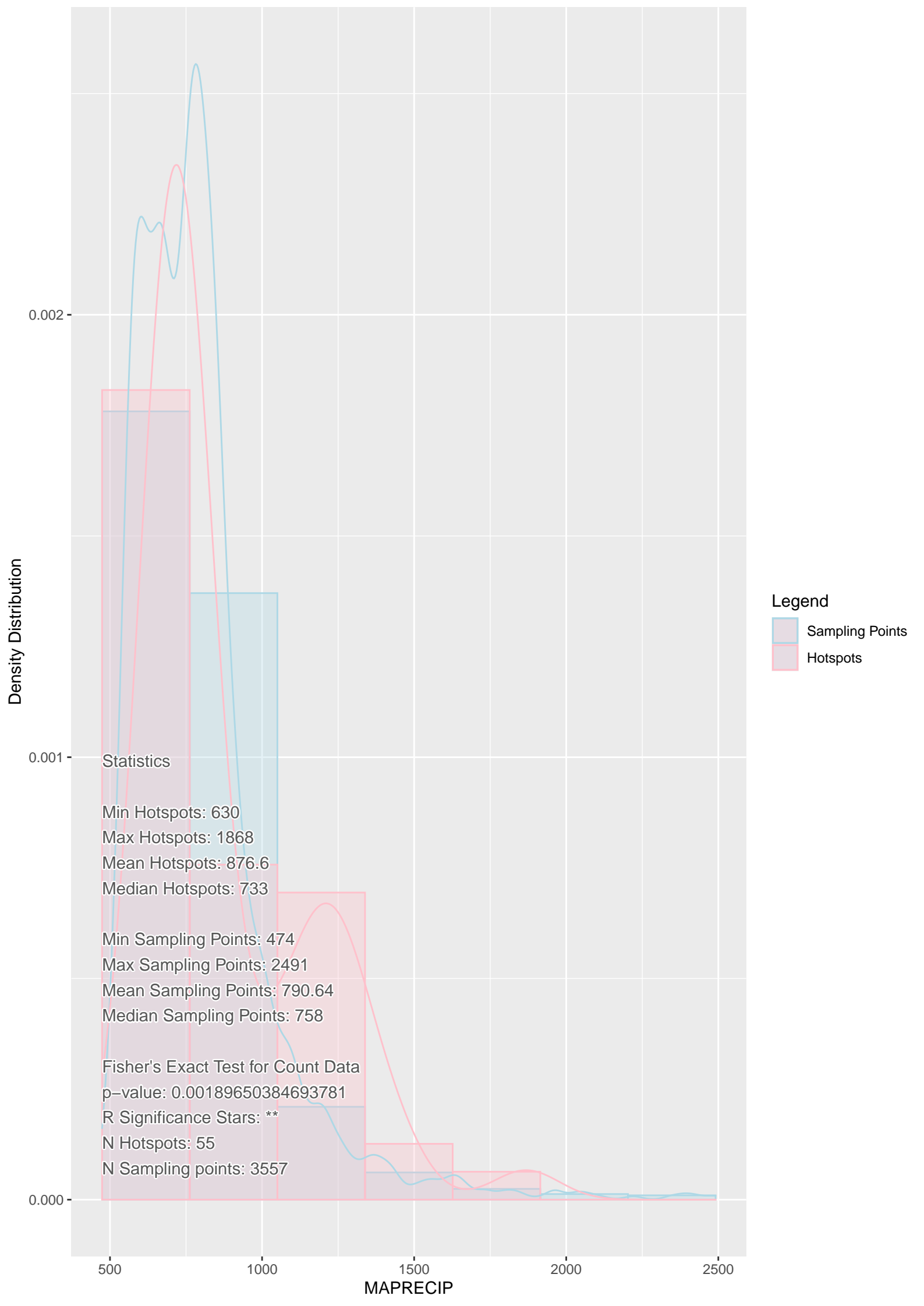

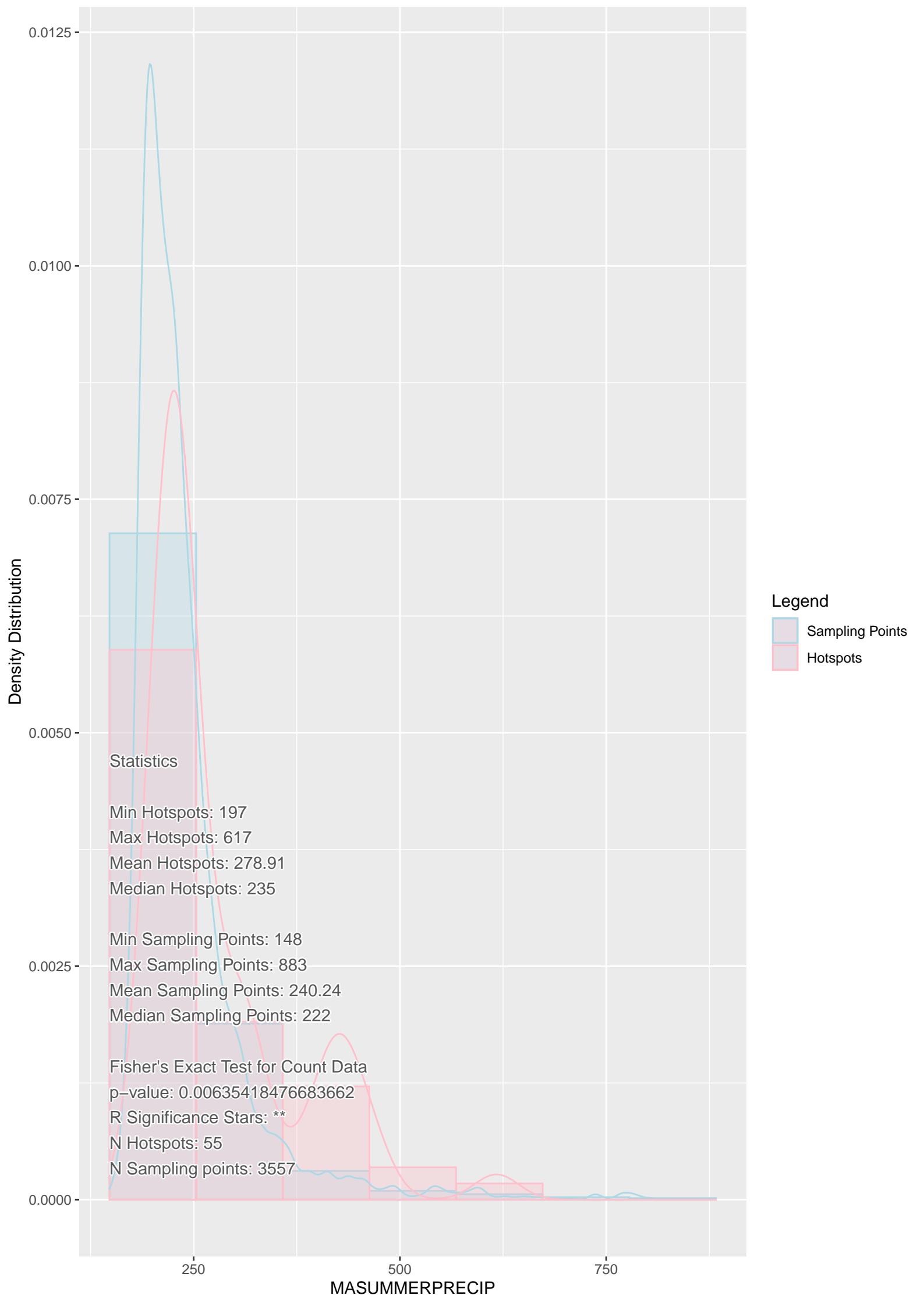

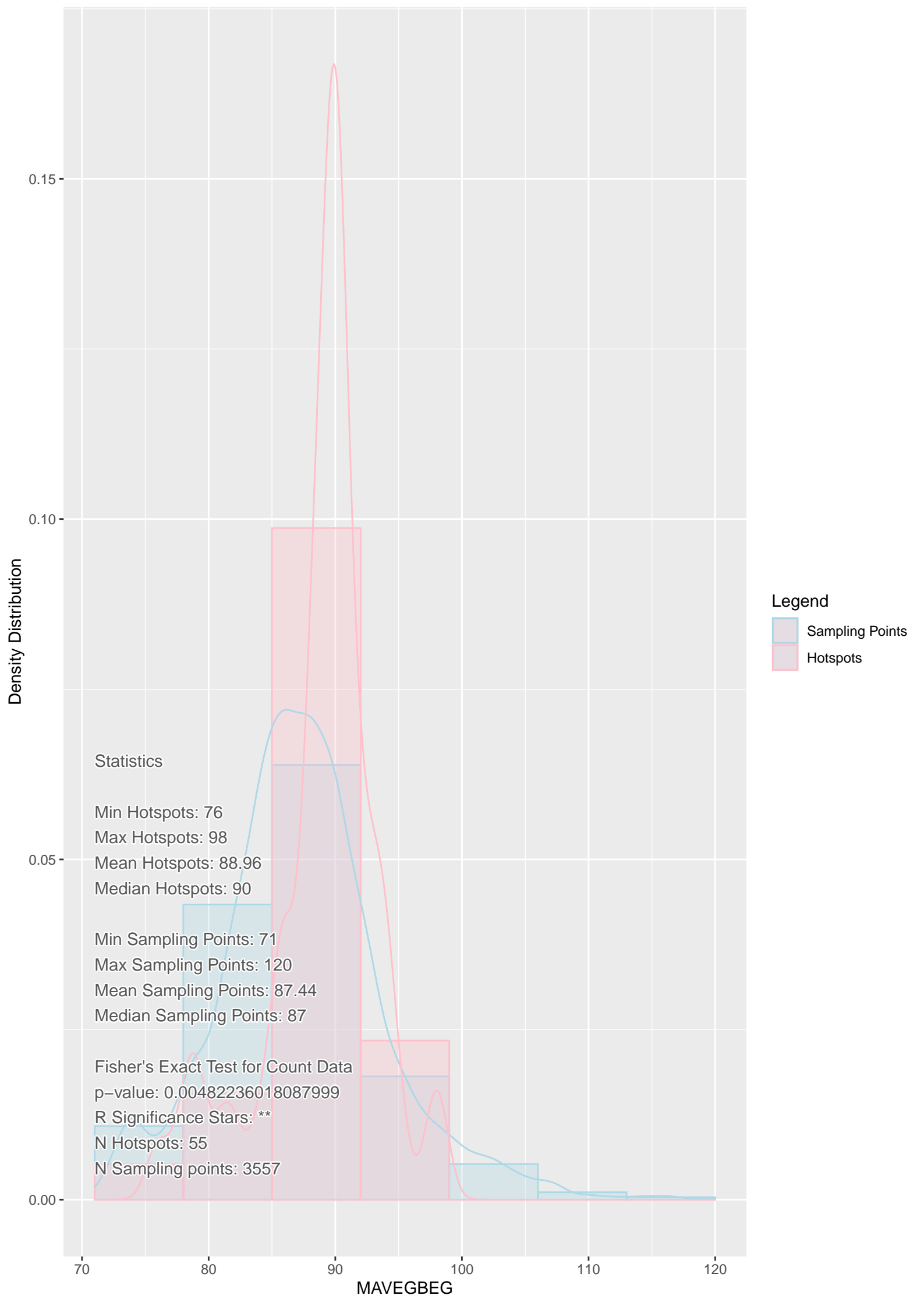

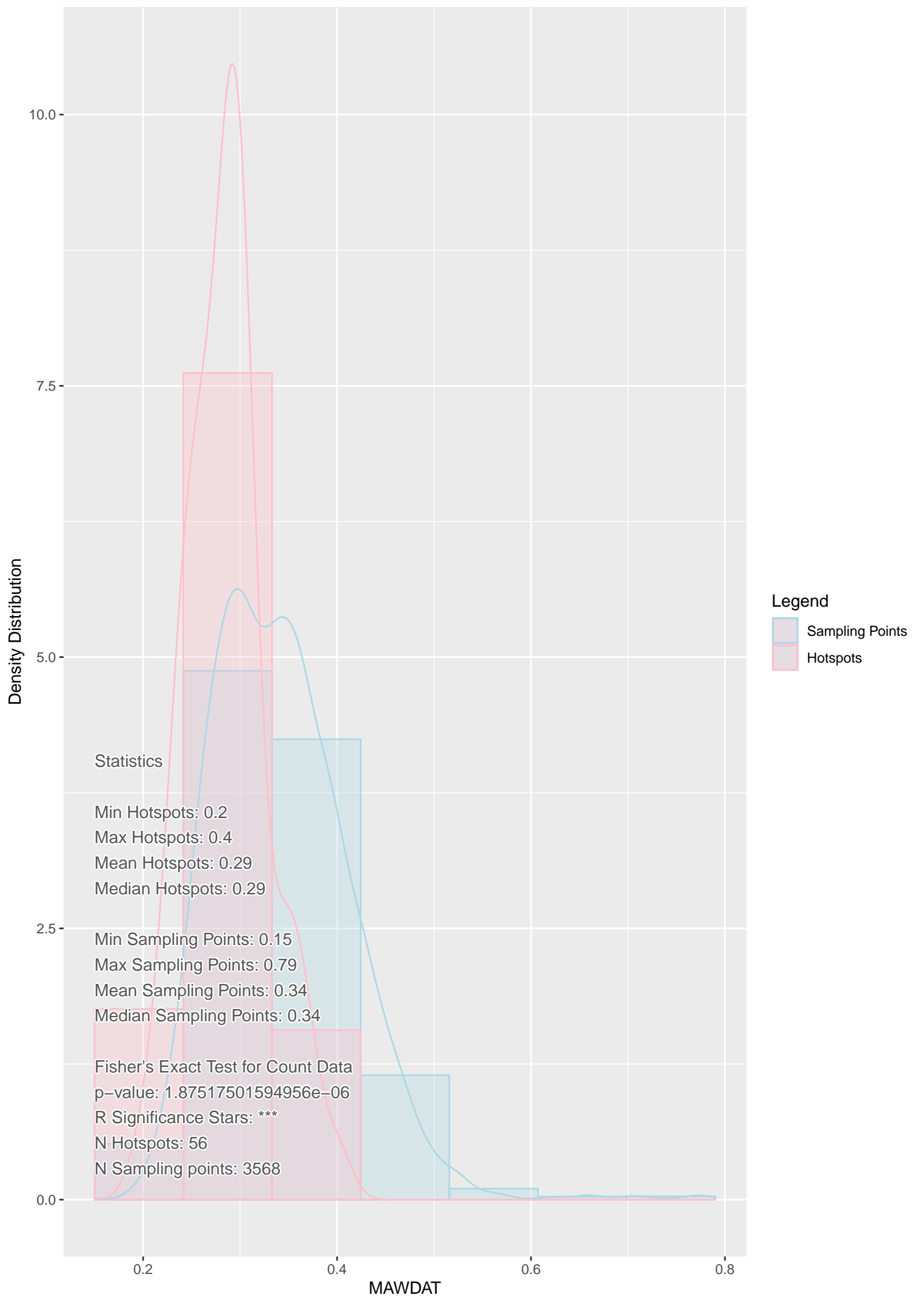

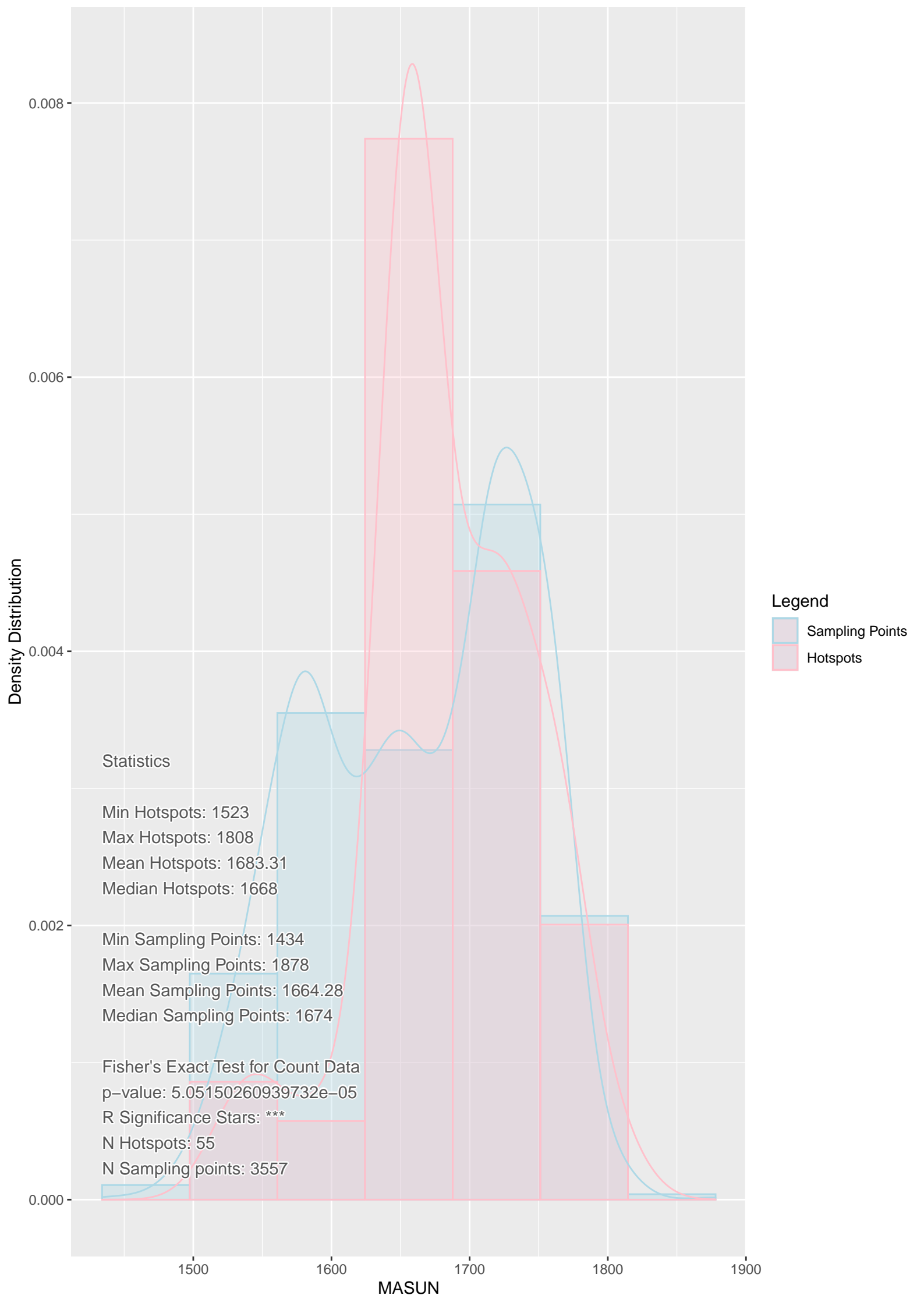

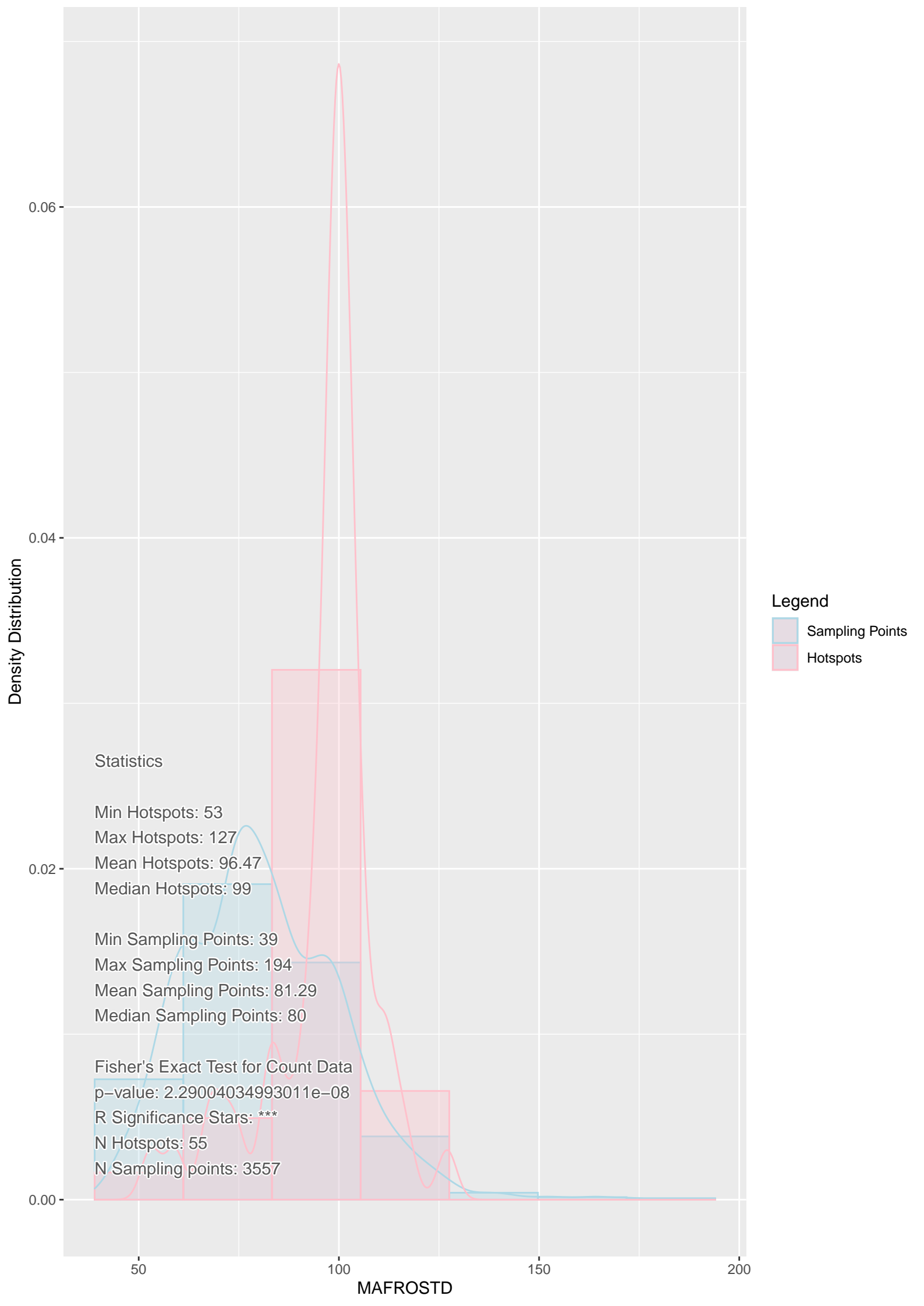

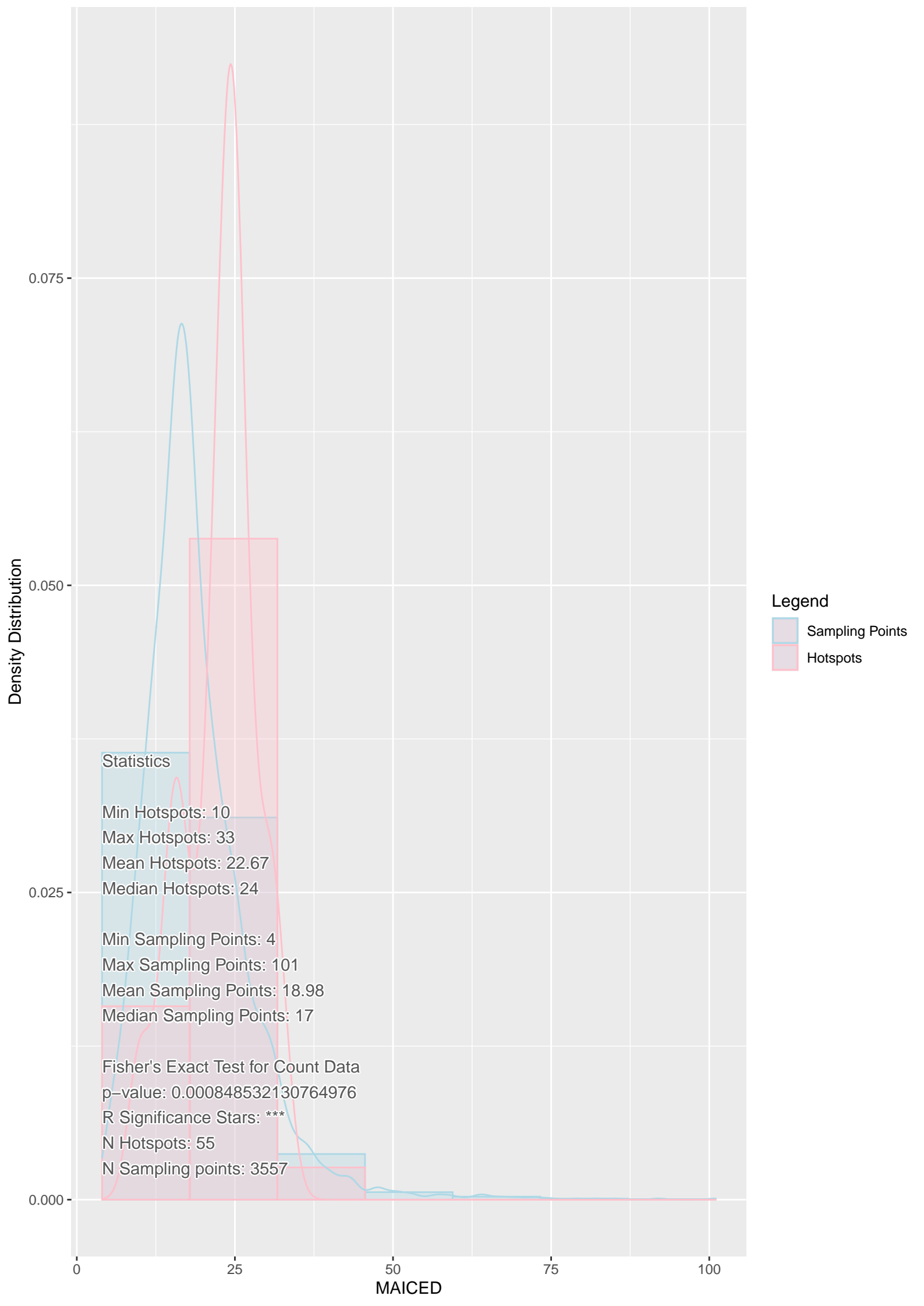

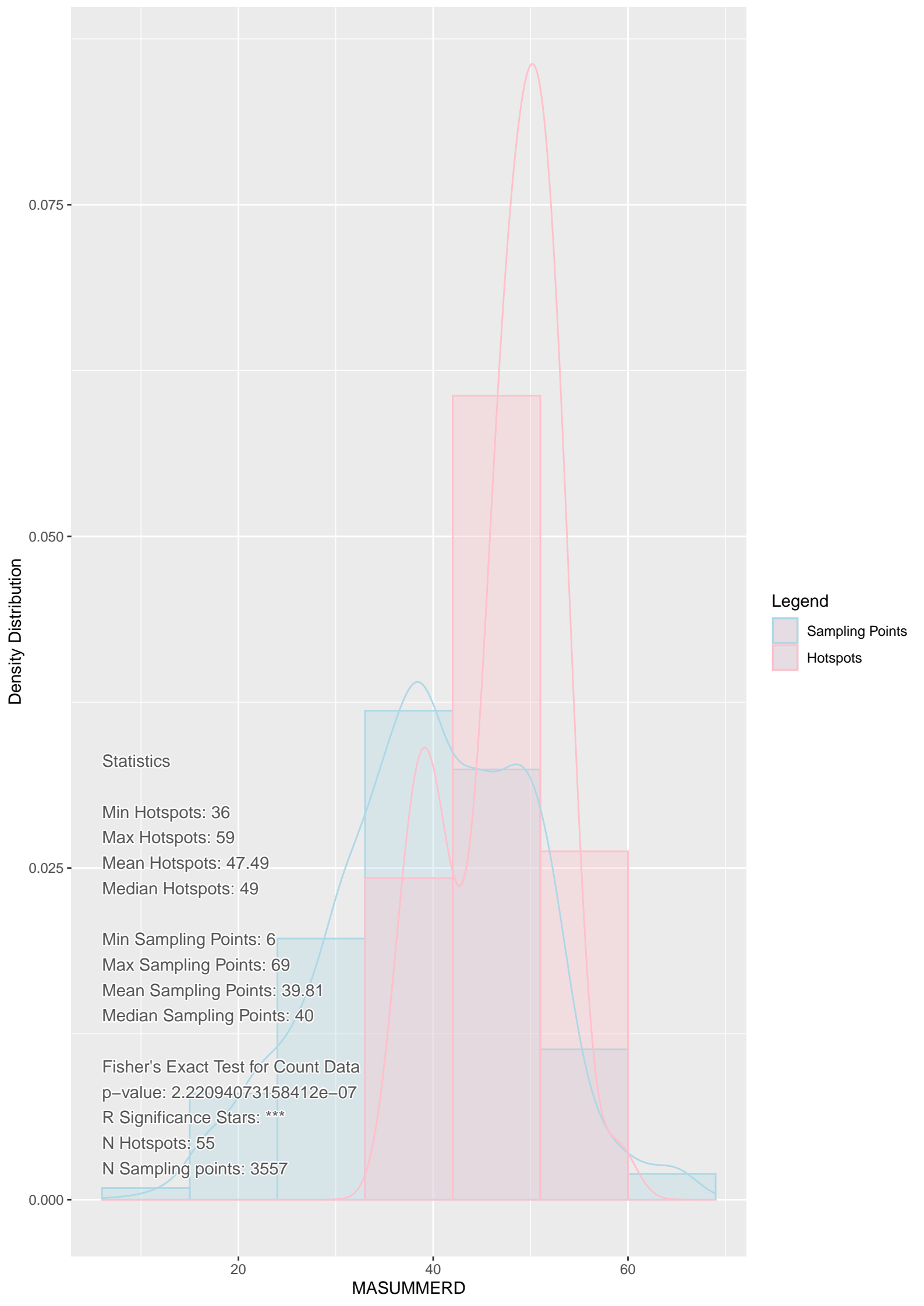

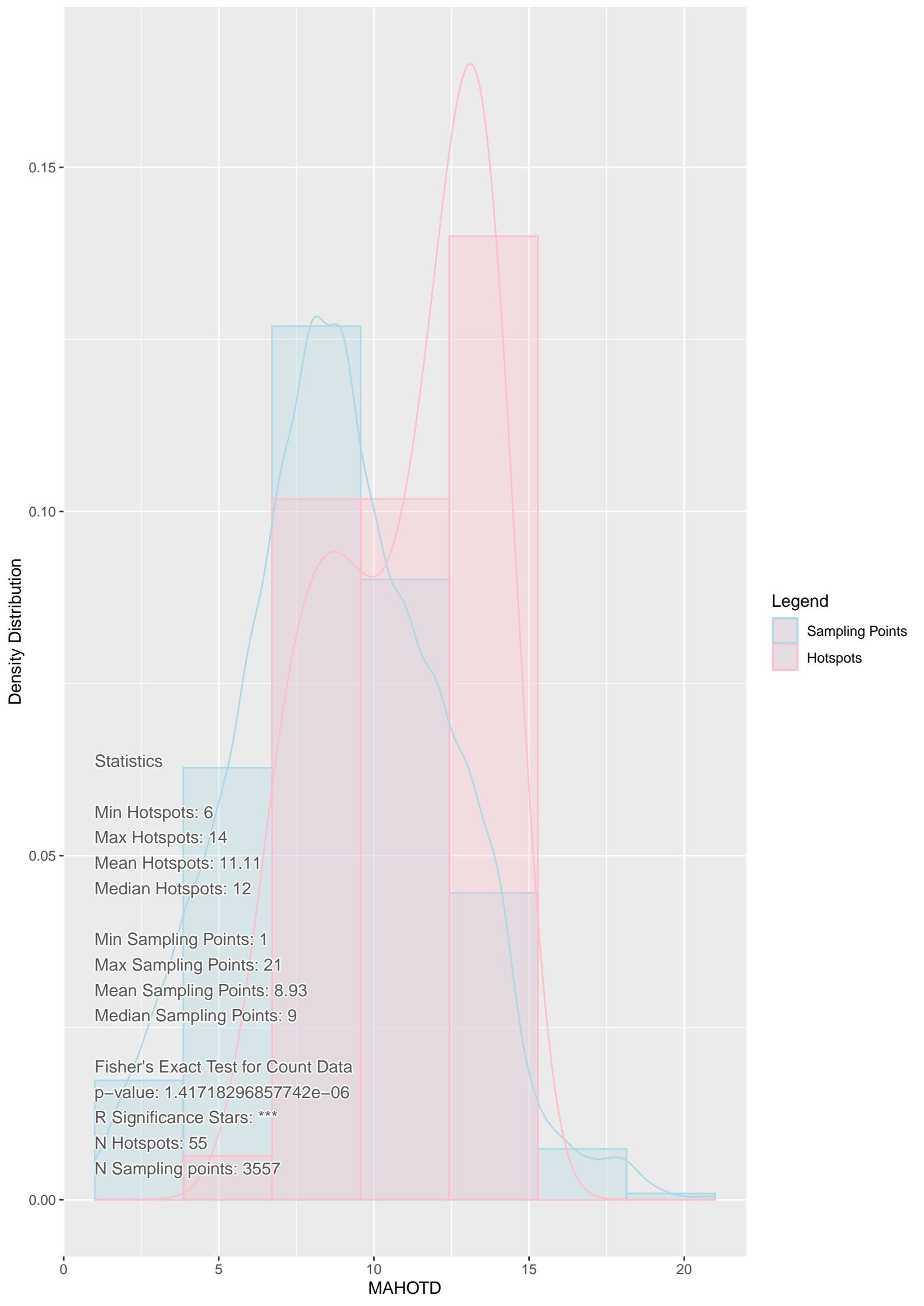

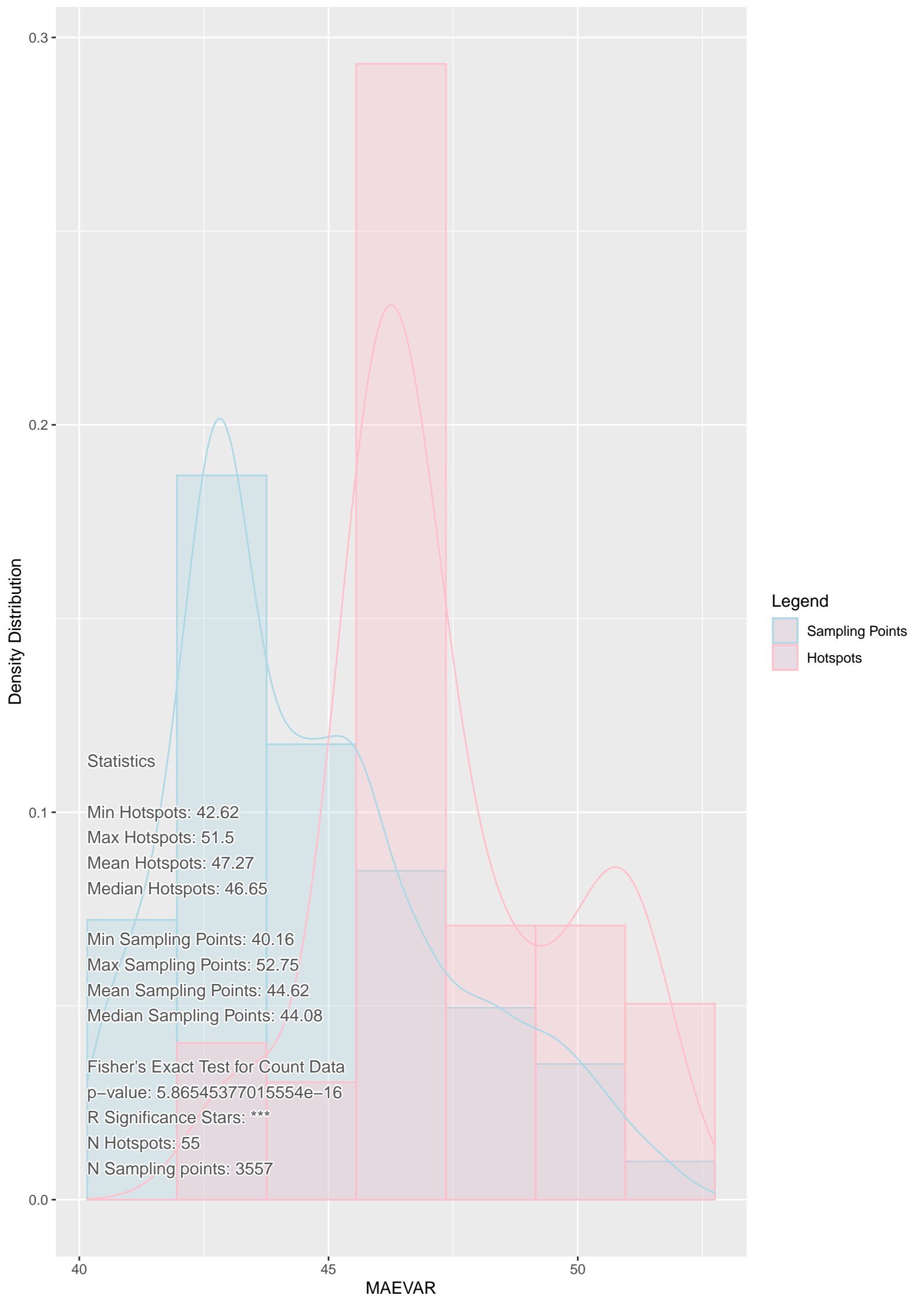

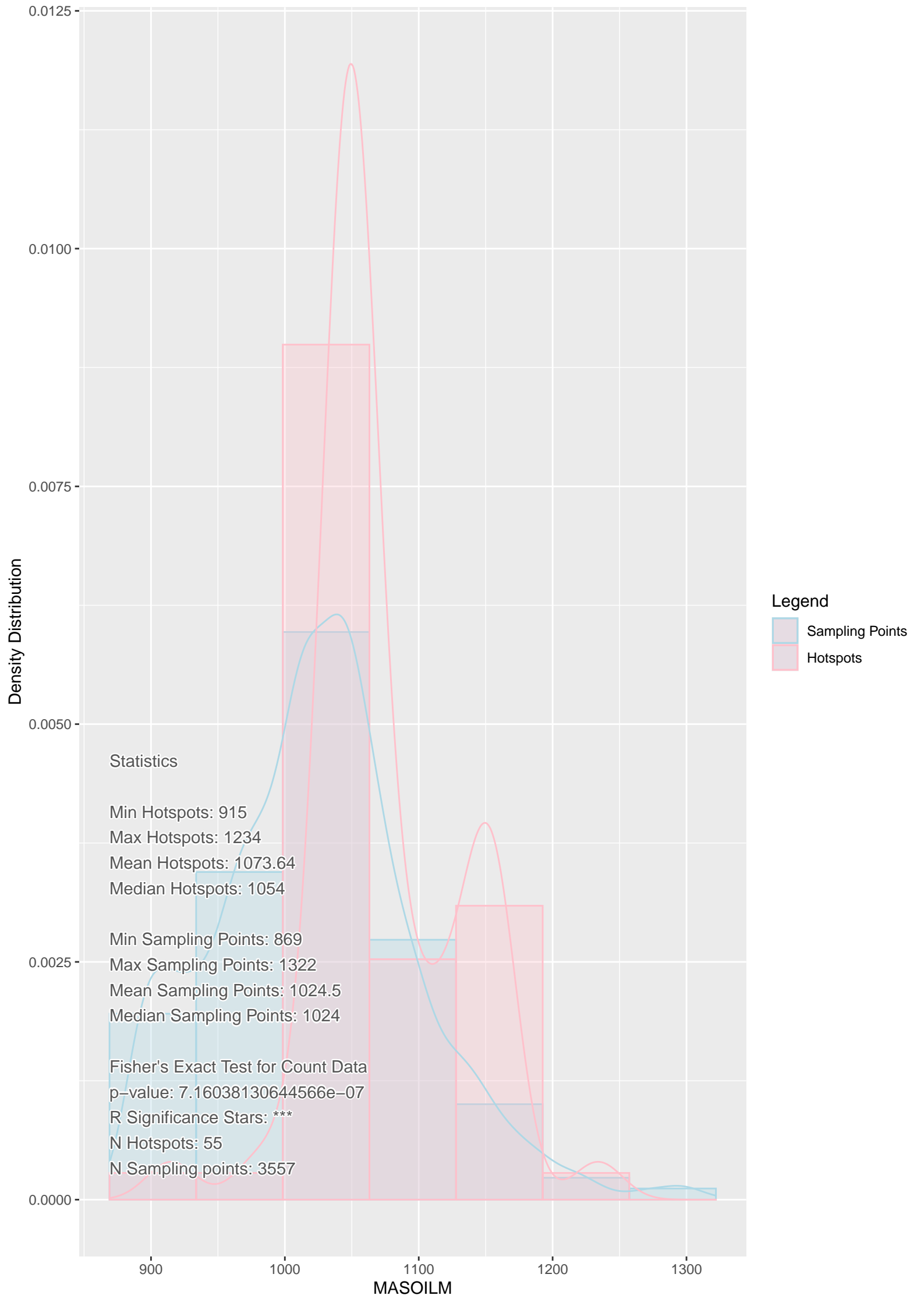

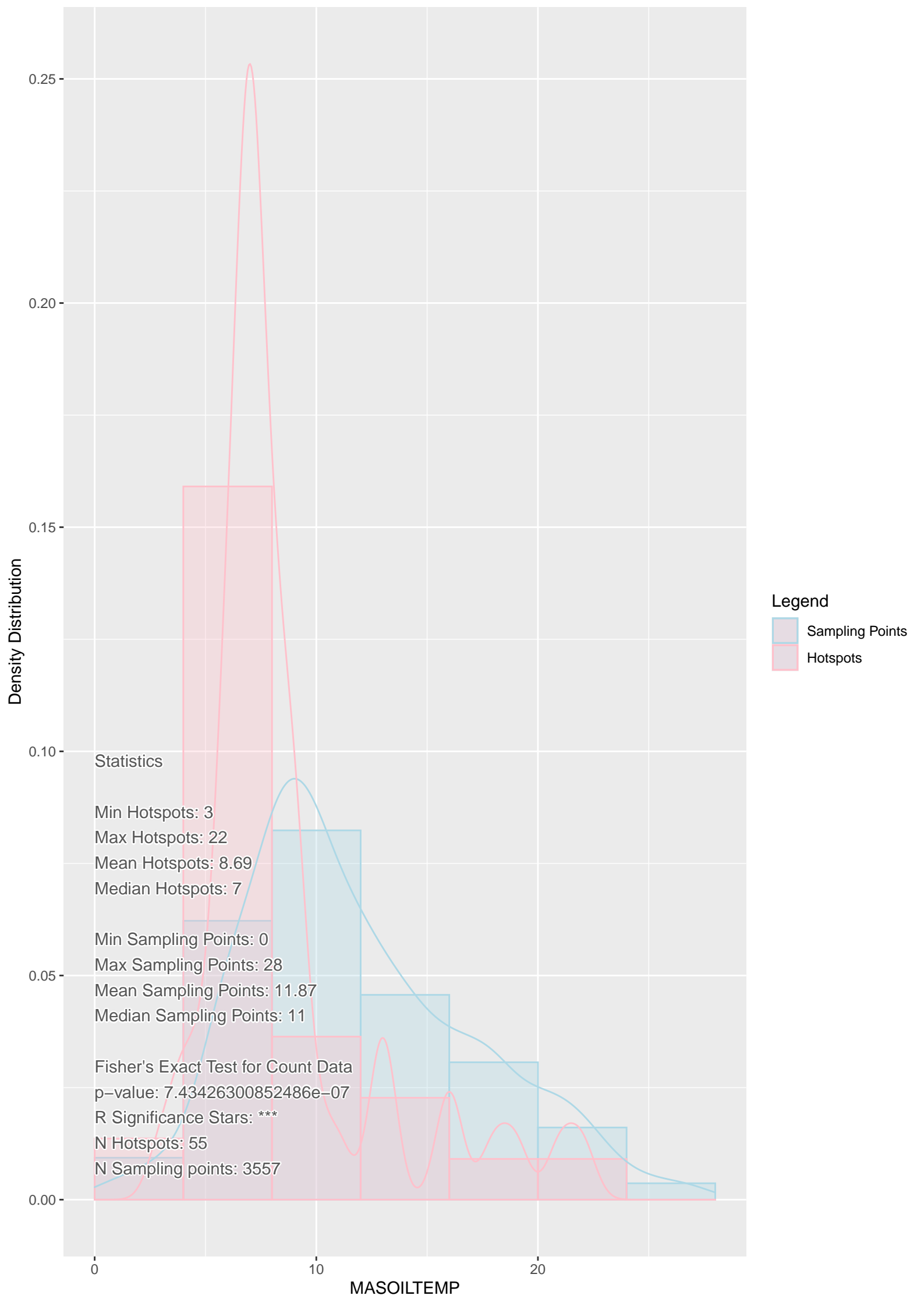

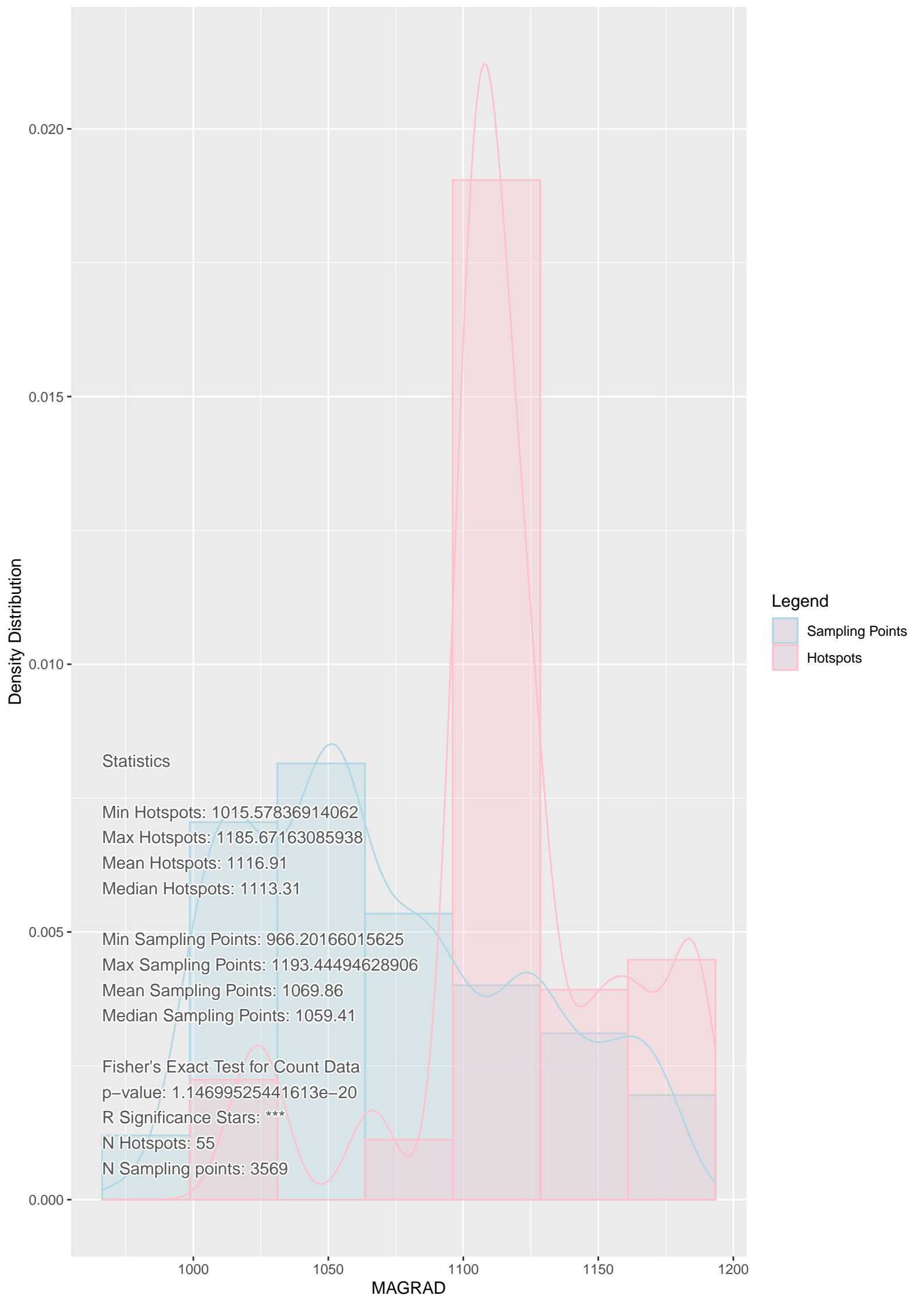

Supplement: Supplementary file 1 [file ijerph-19-11830-s001.zip › suppl. fig 2.pdf]

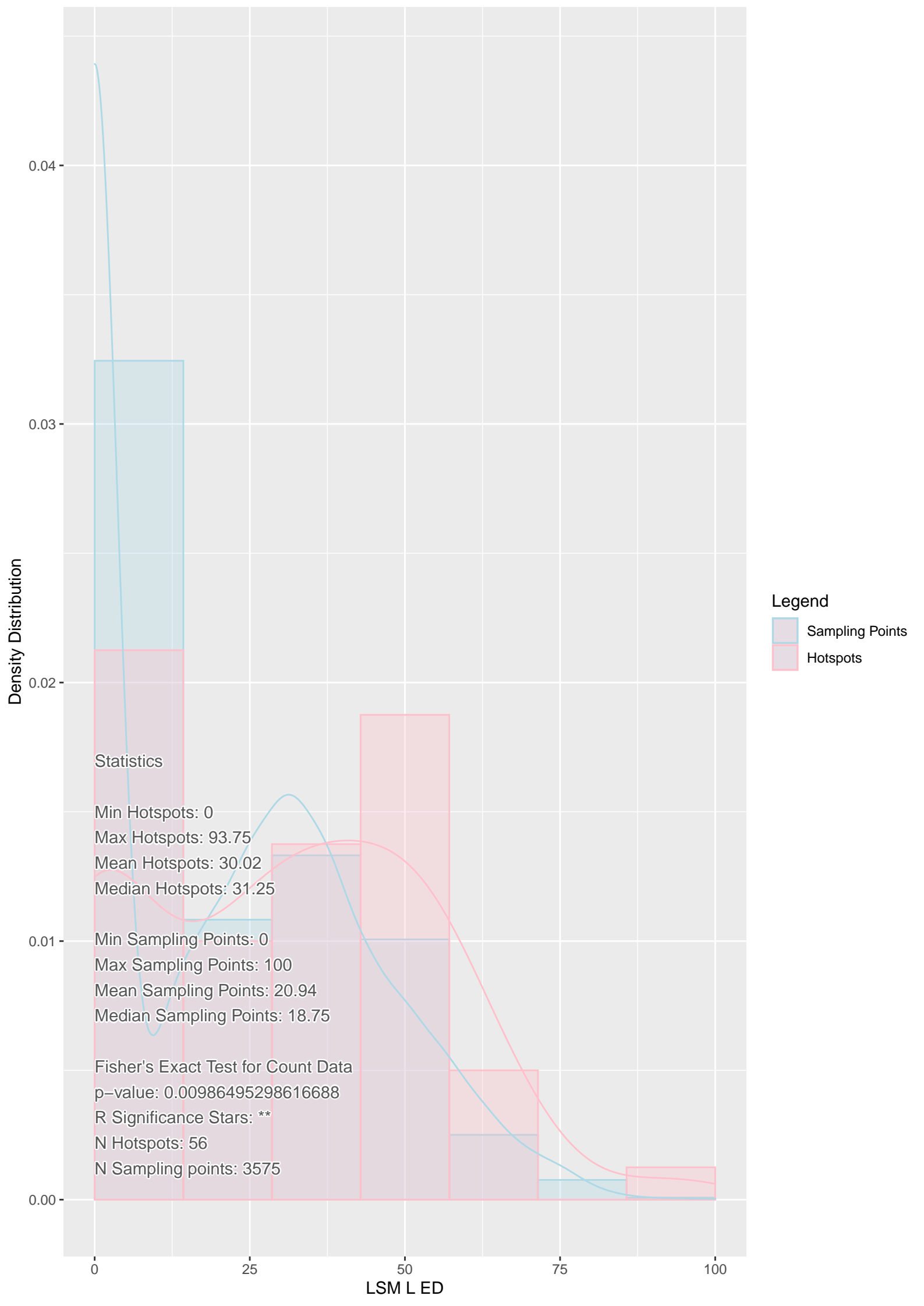

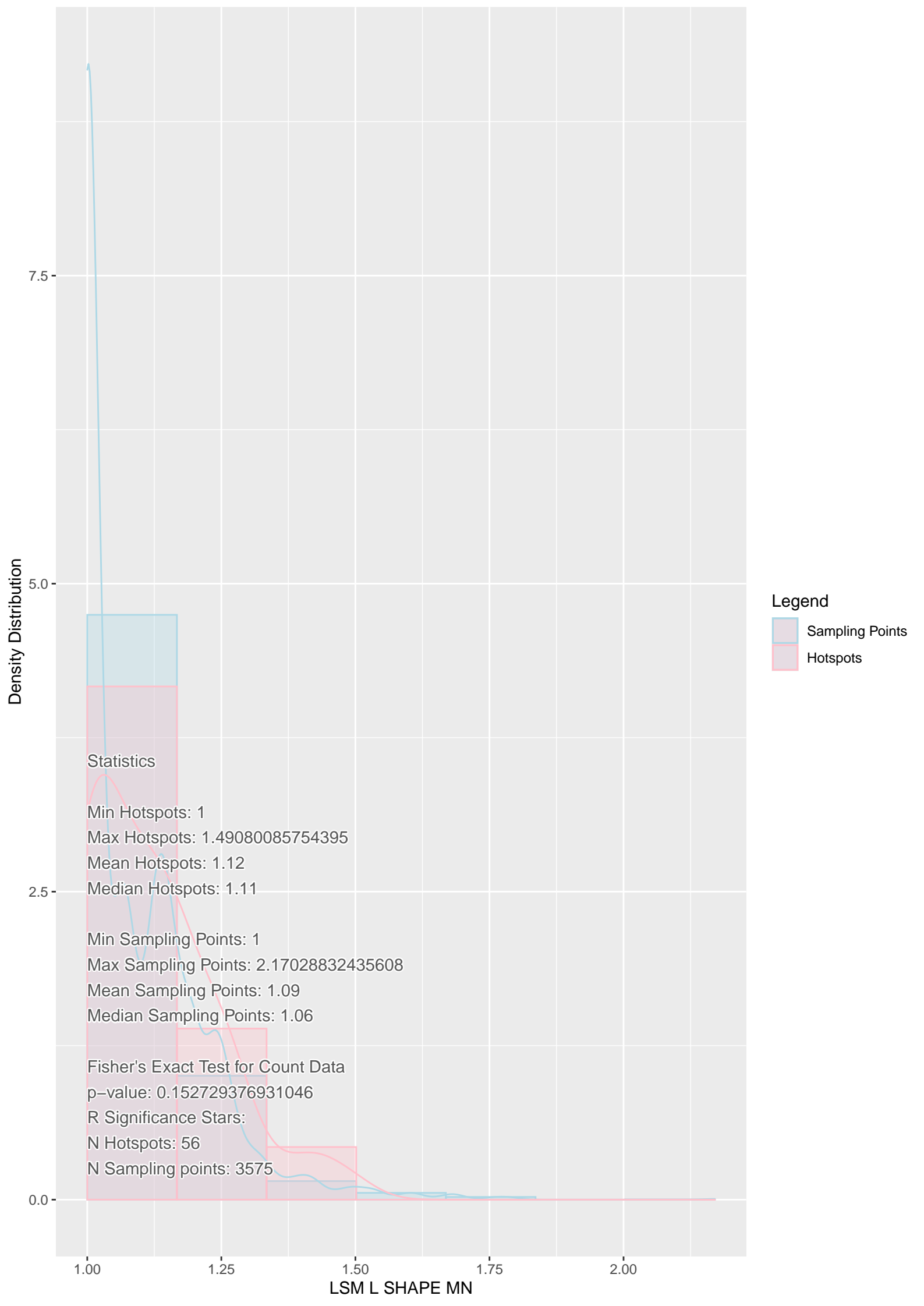

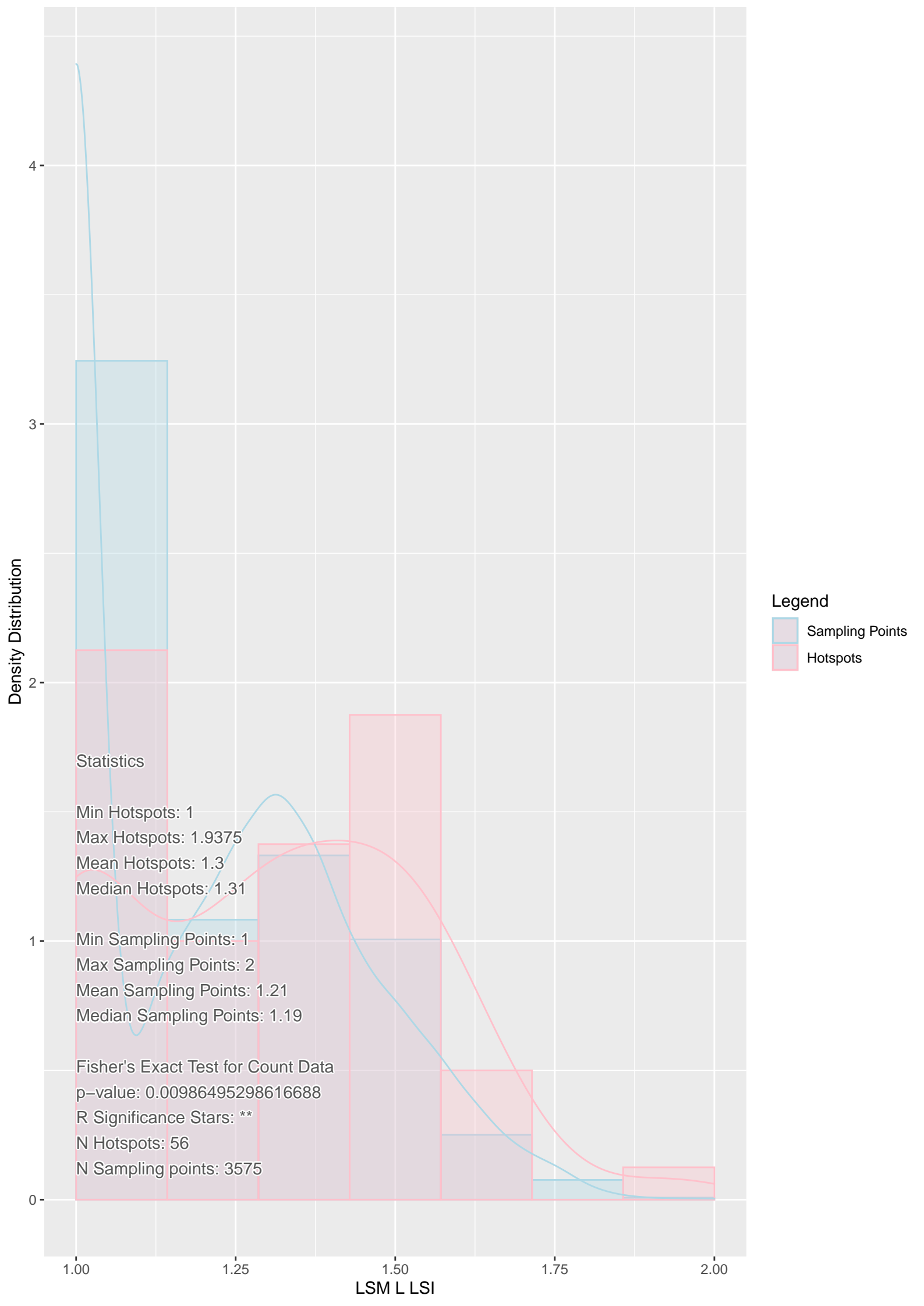

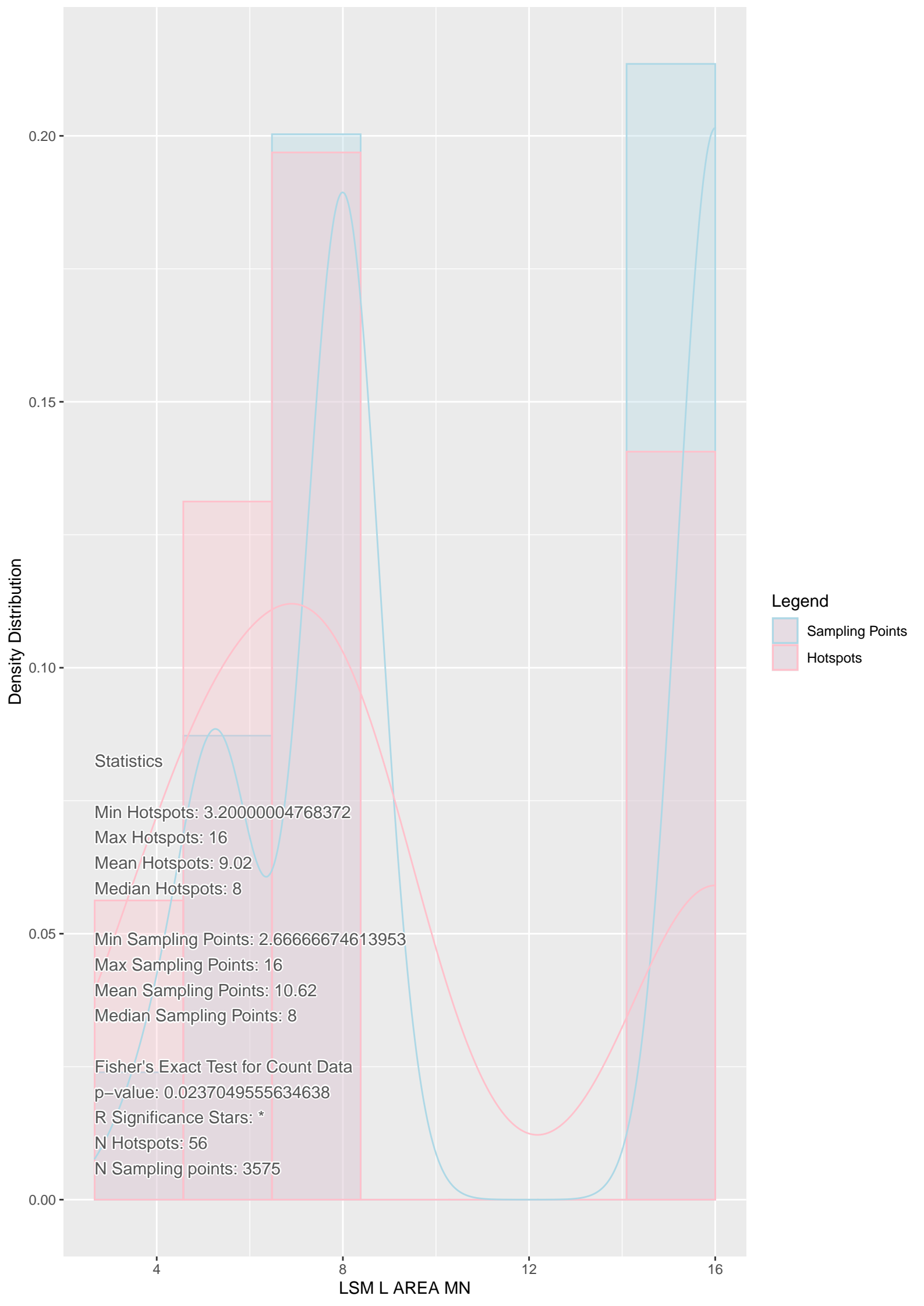

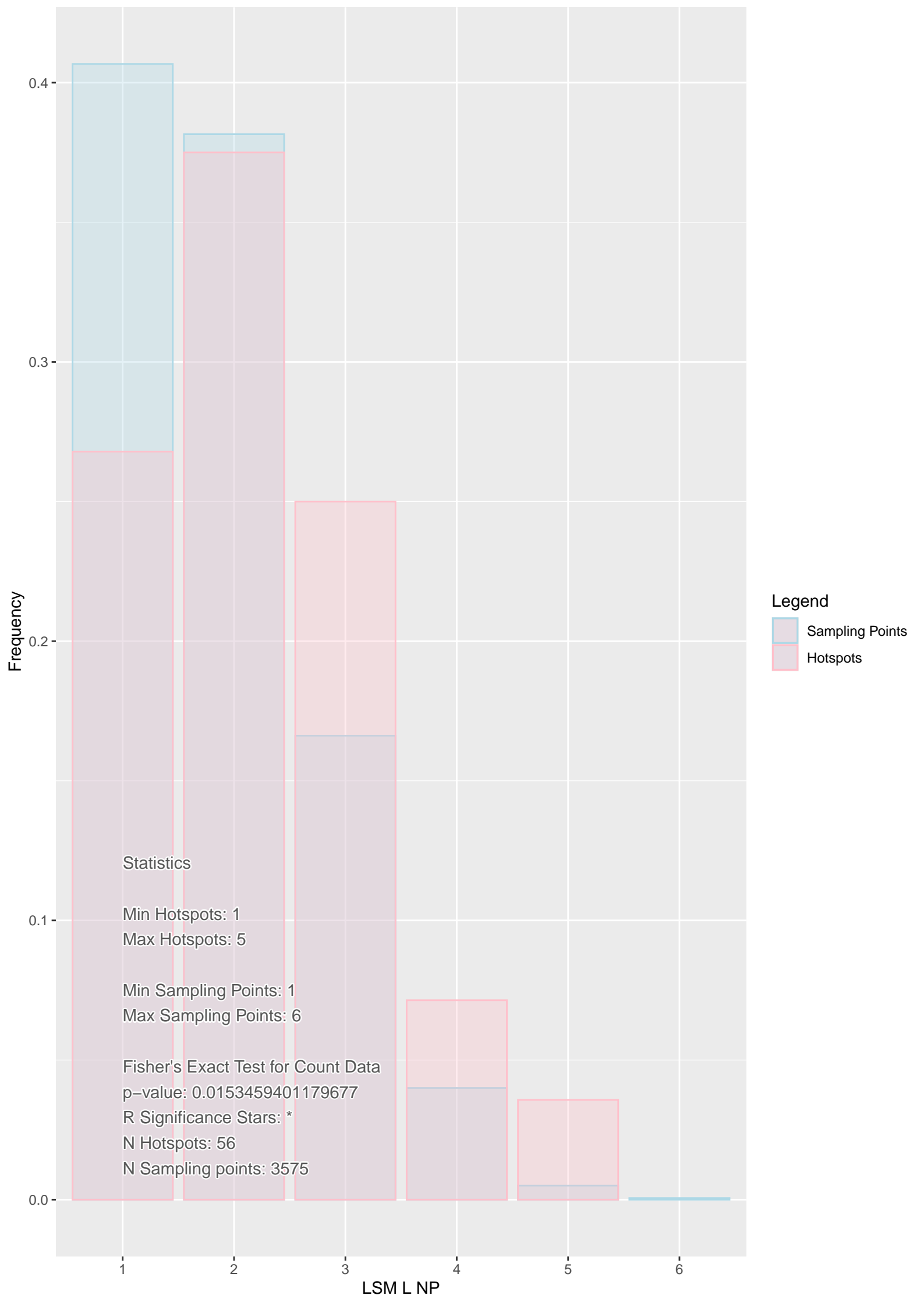

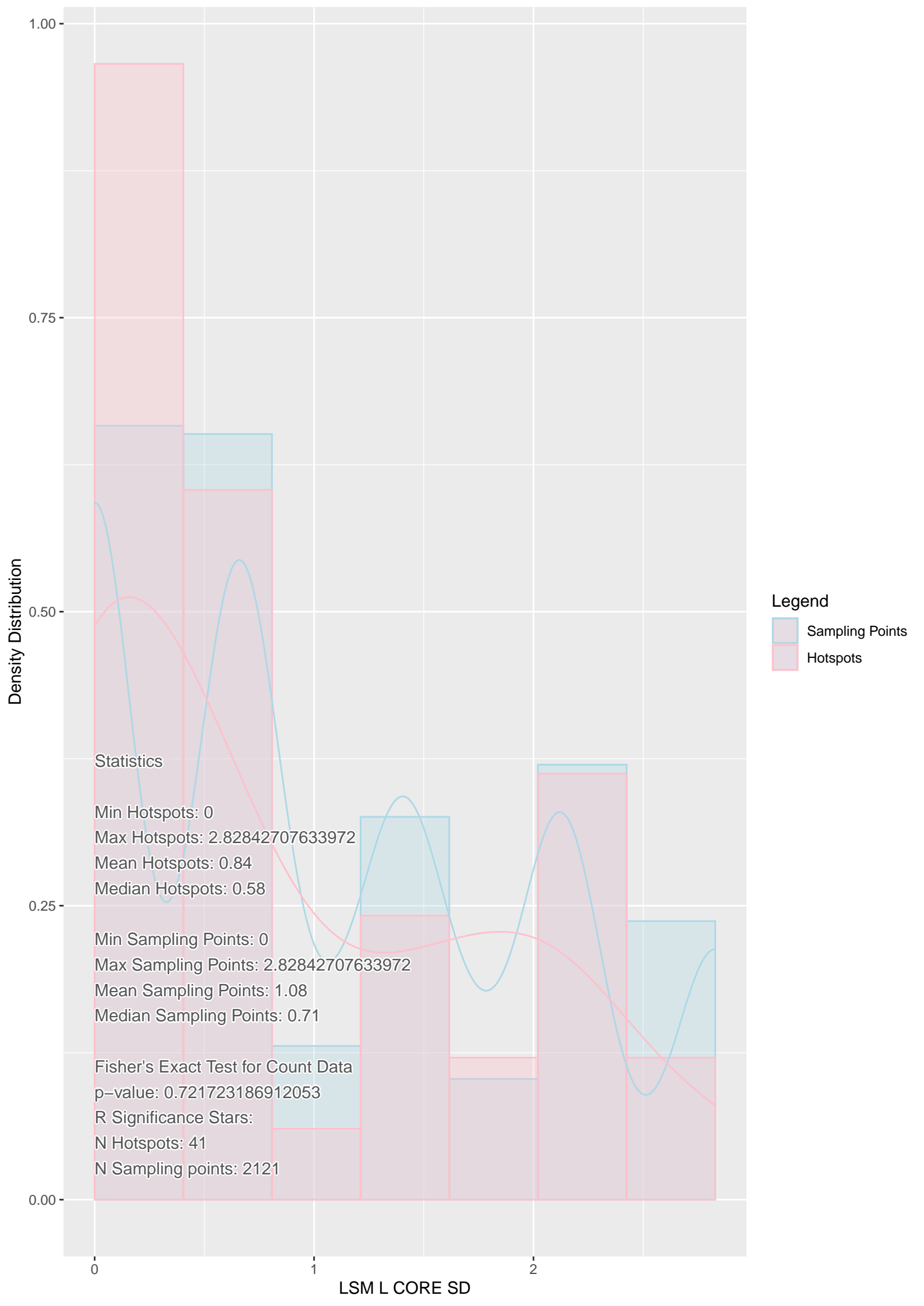

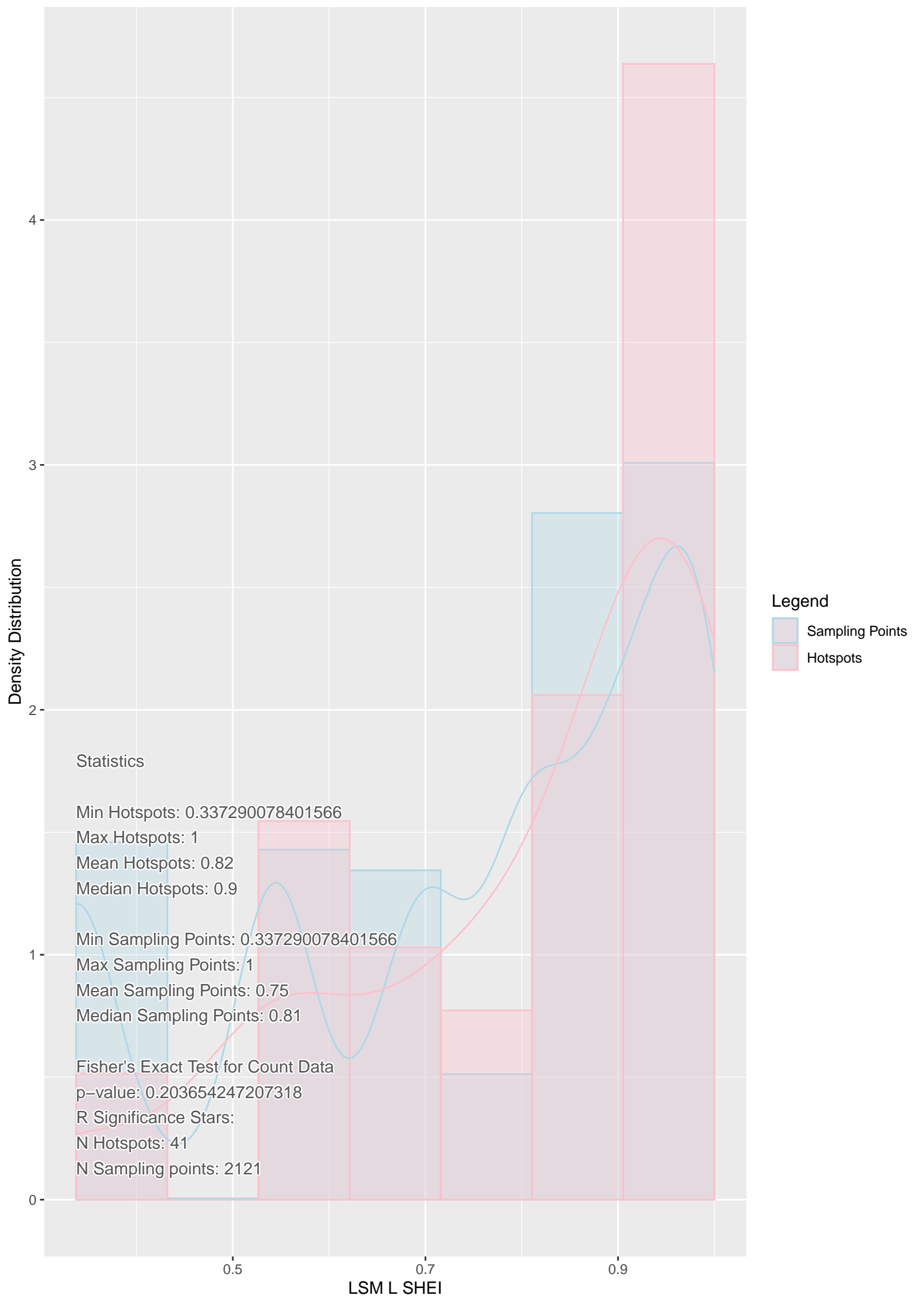

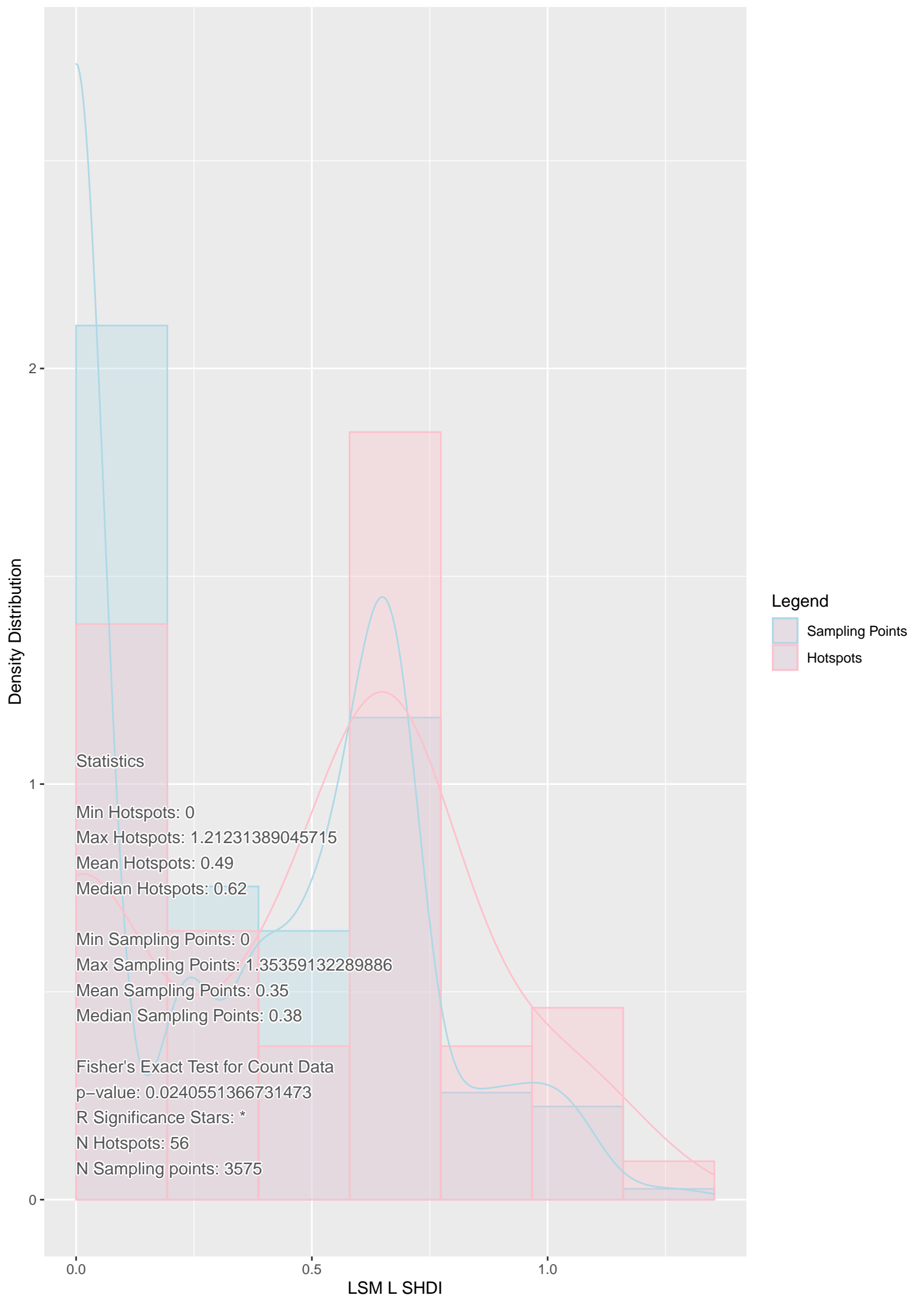

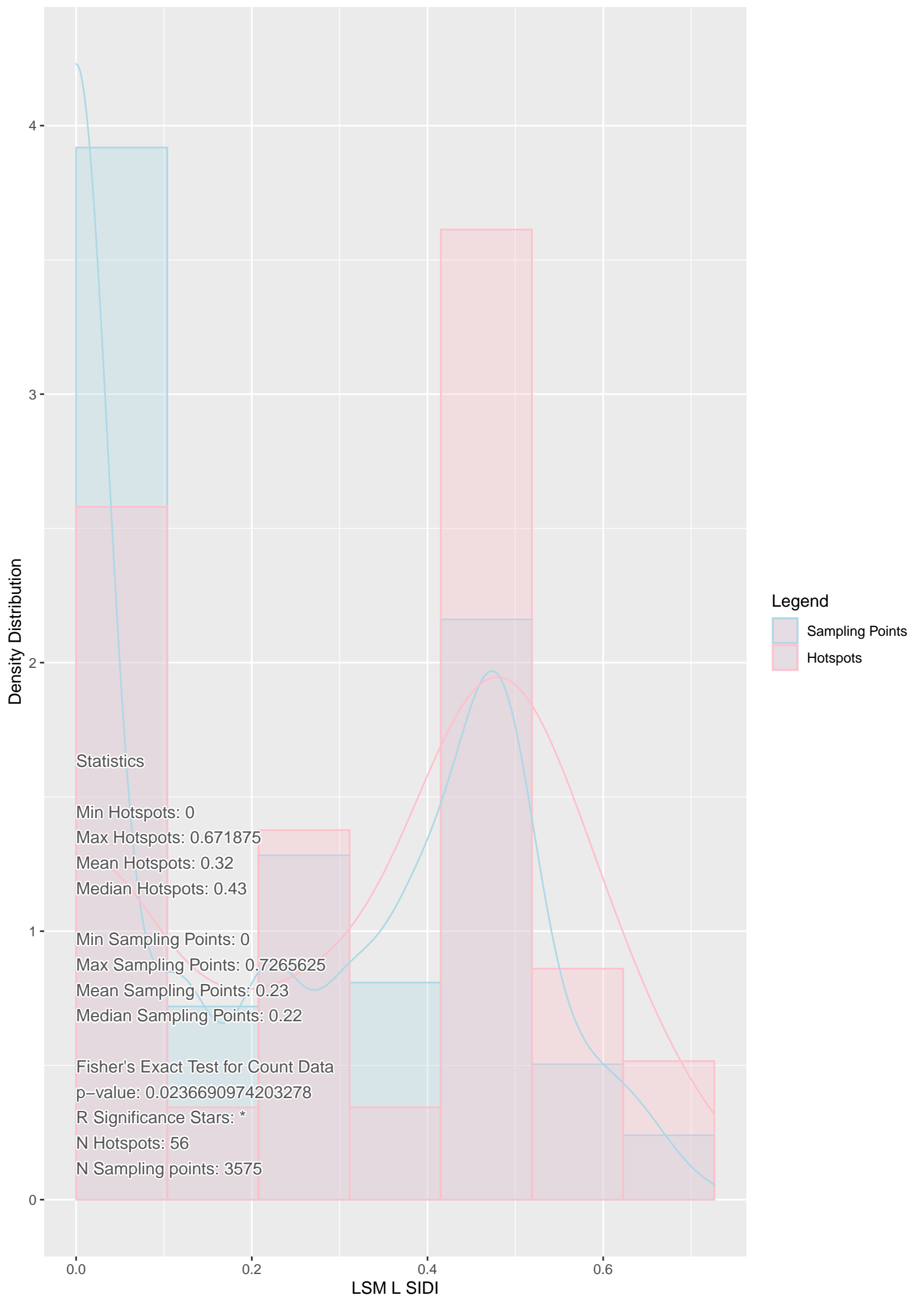

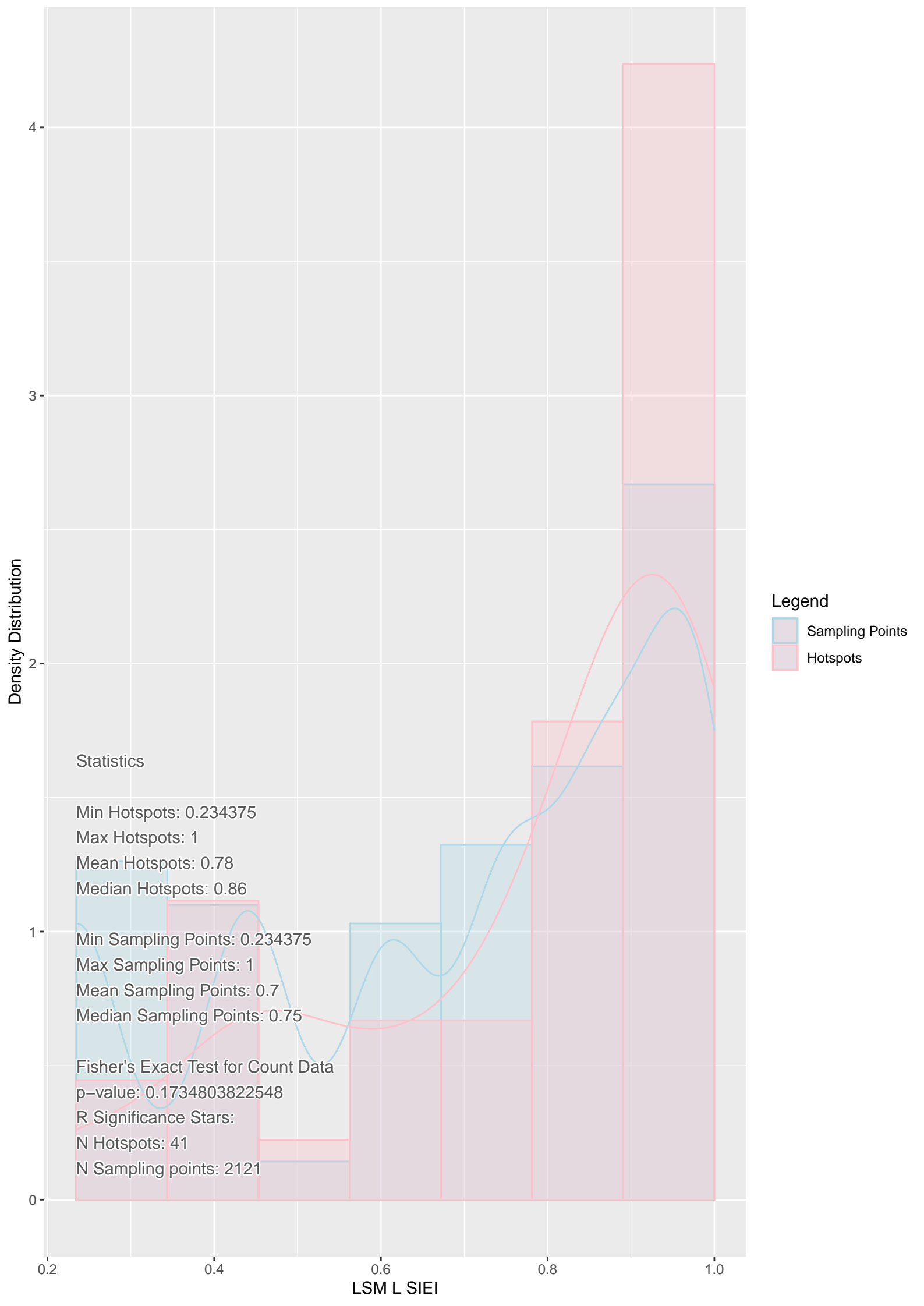

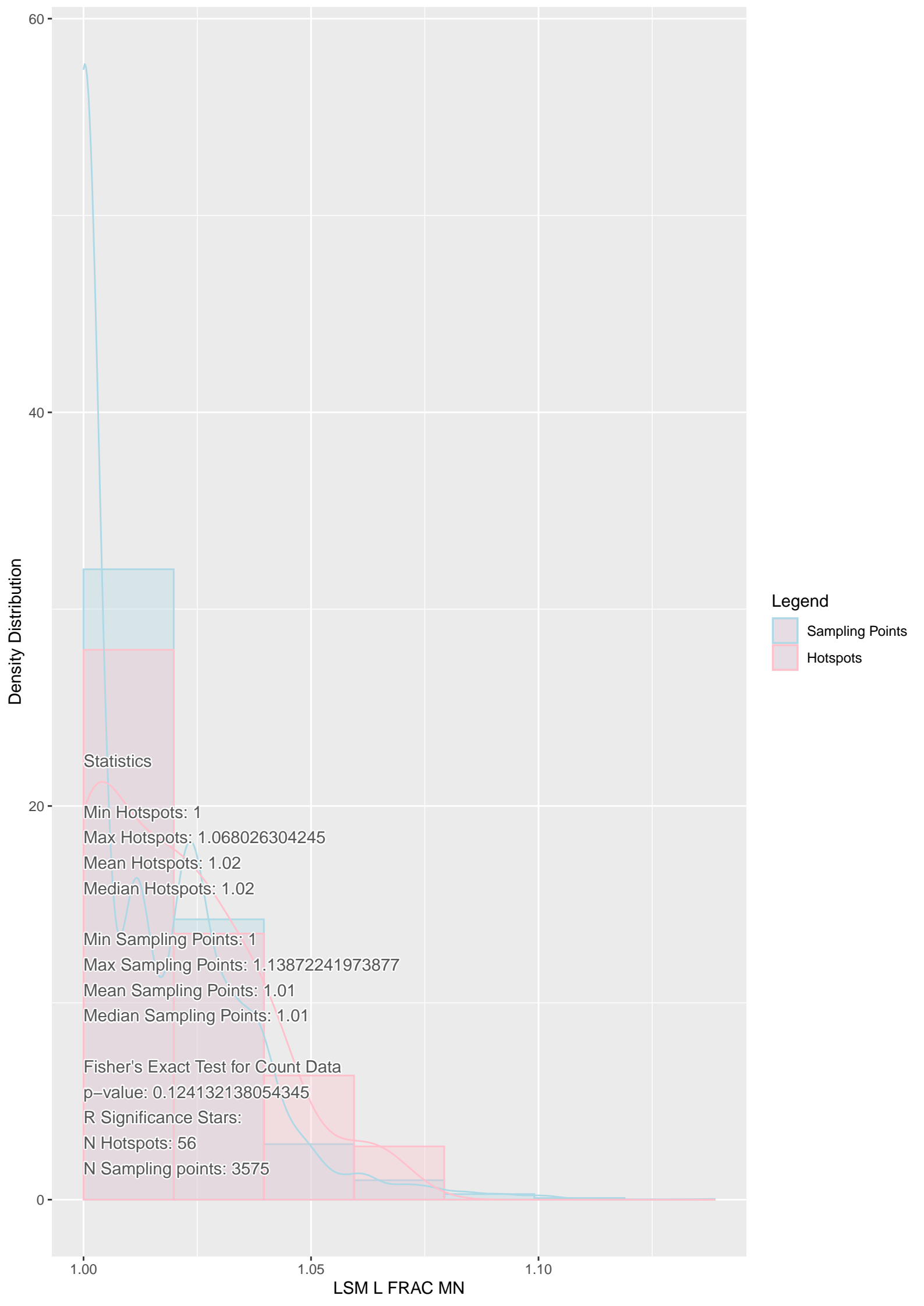

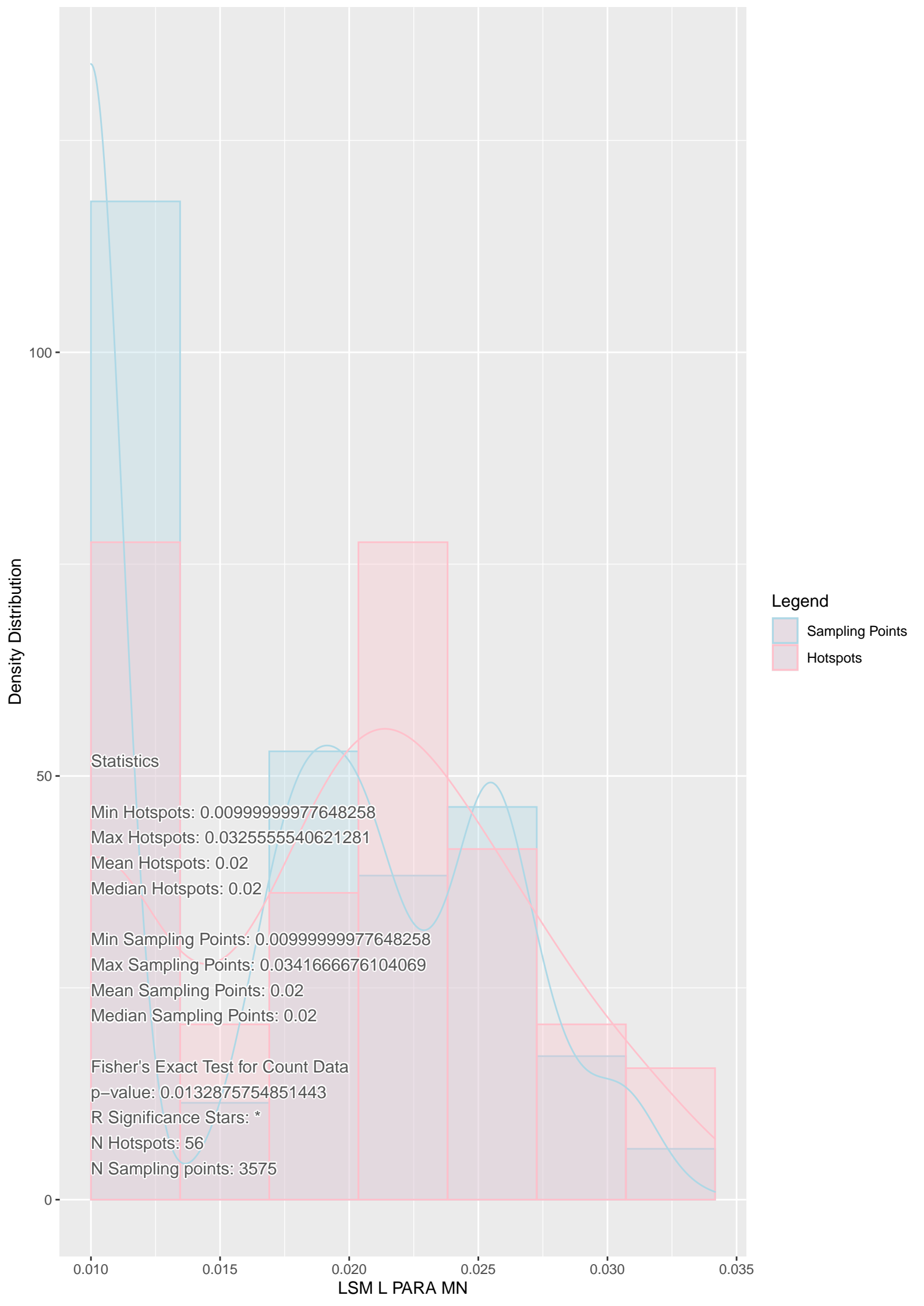

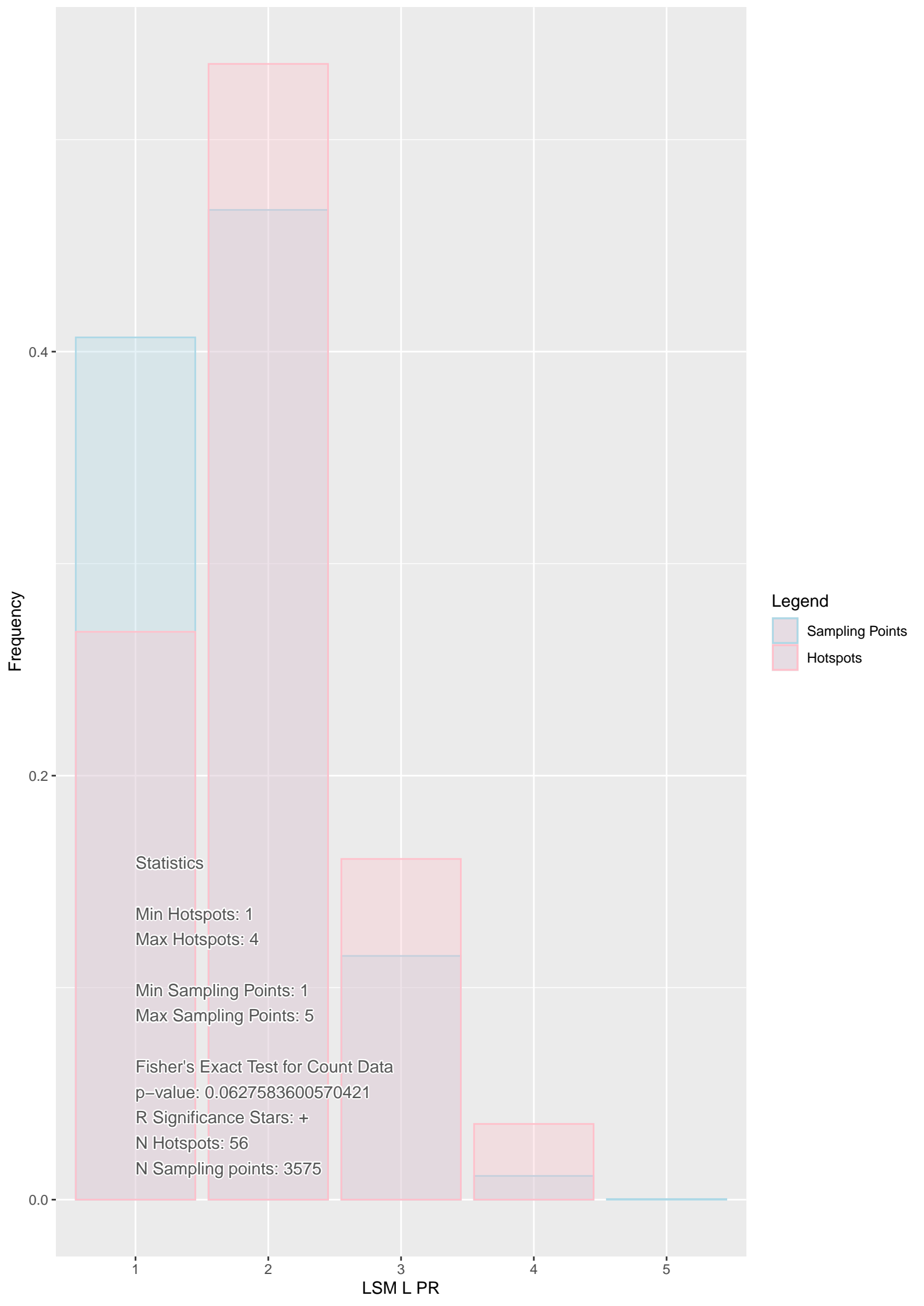

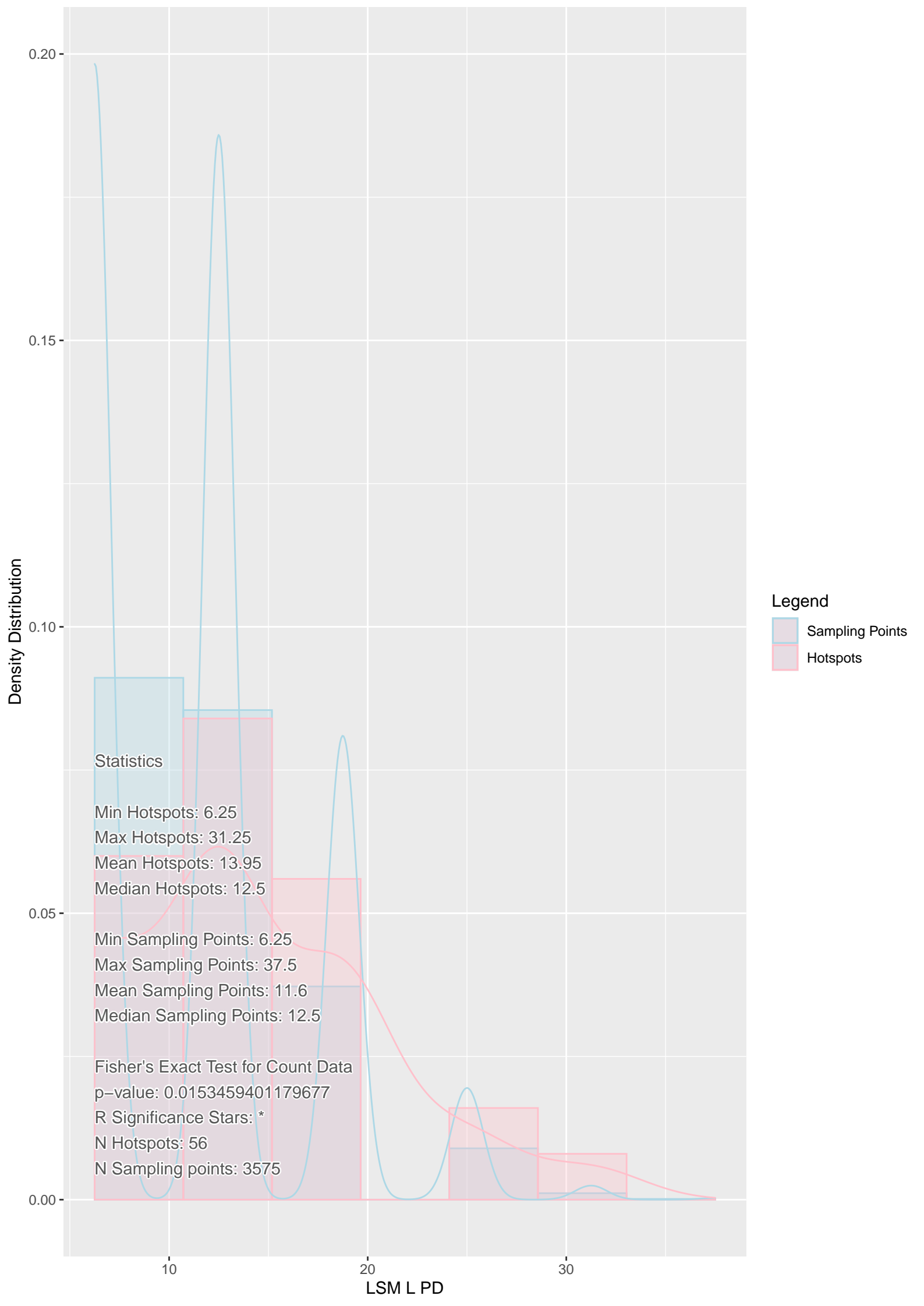

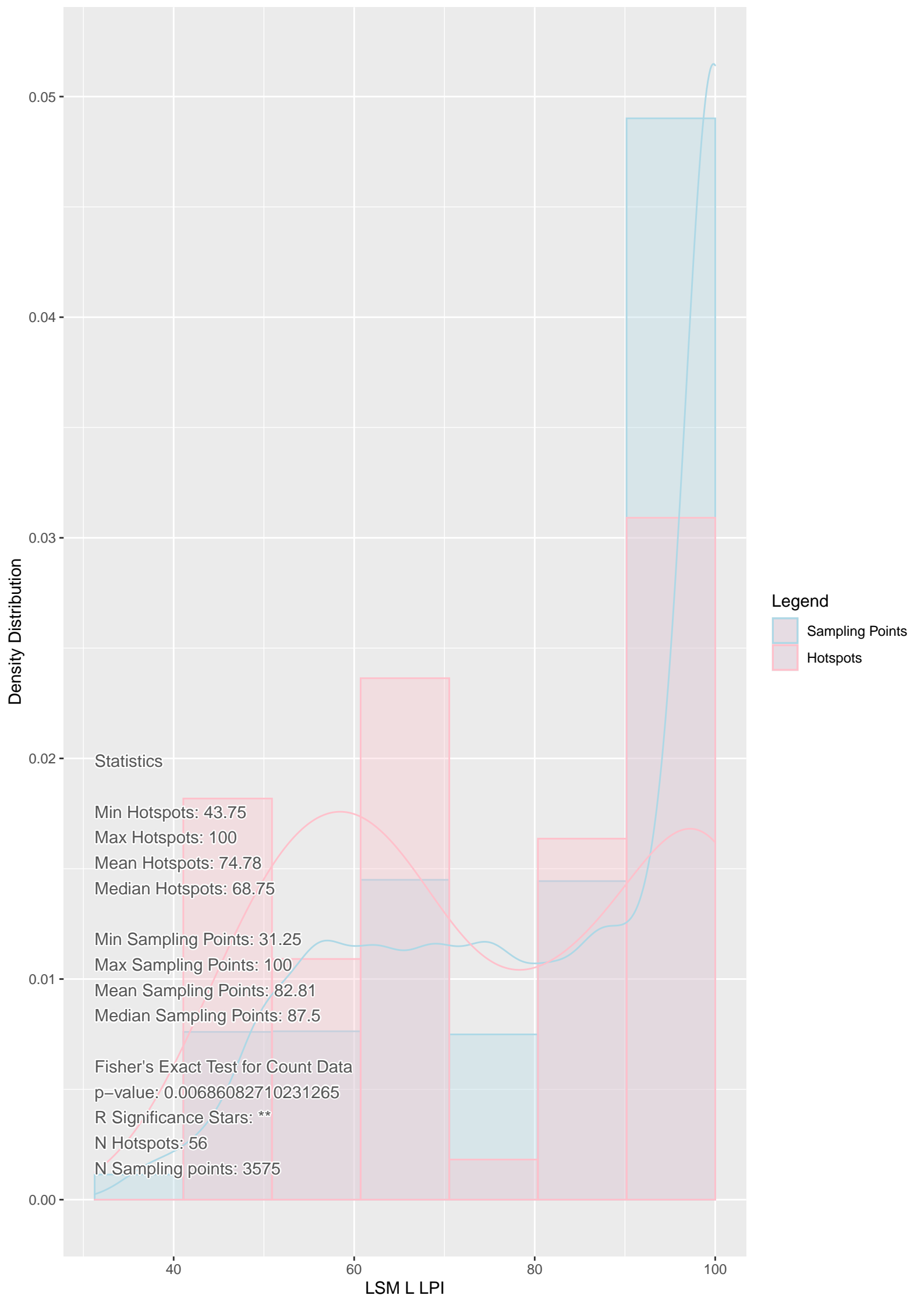

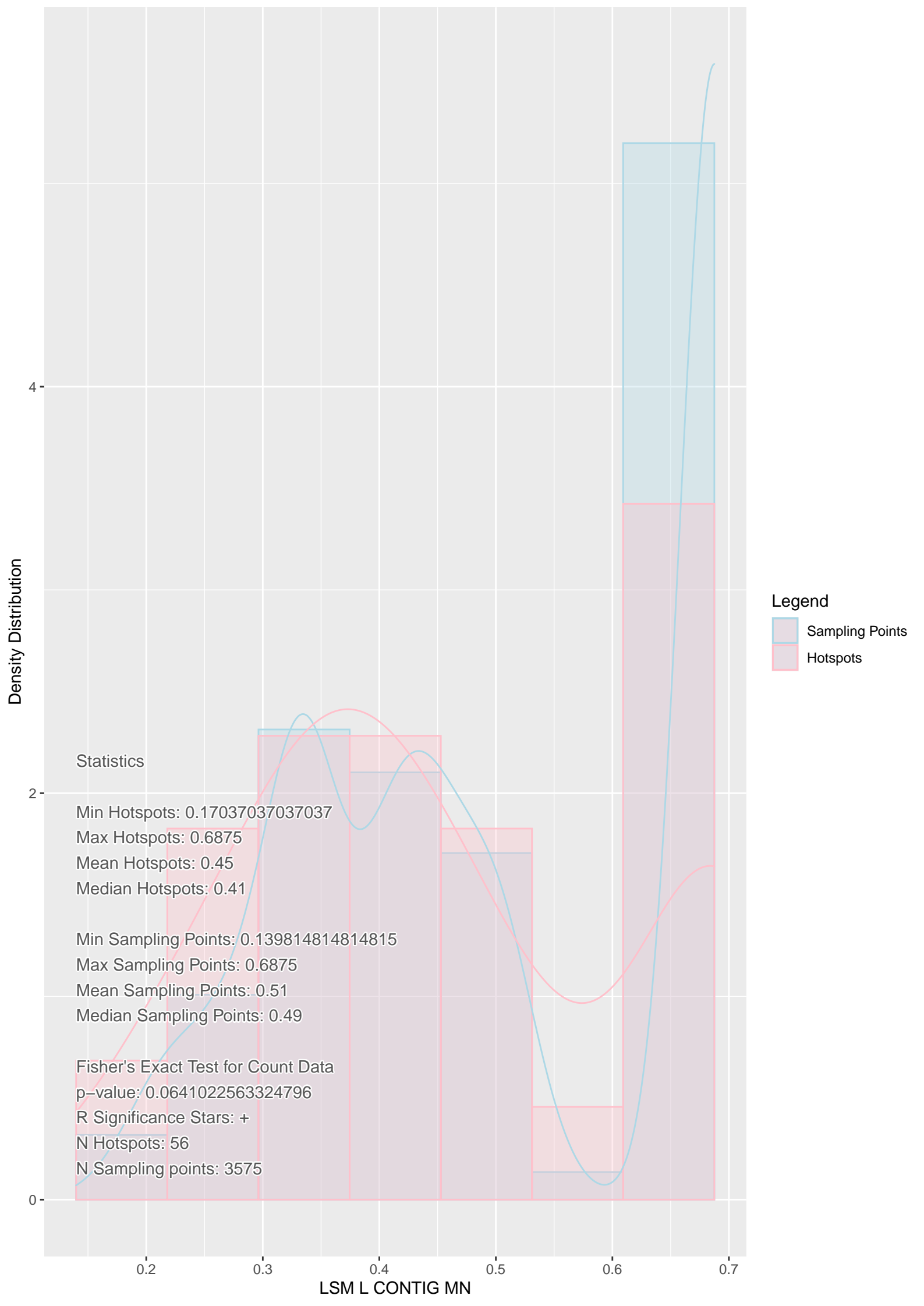

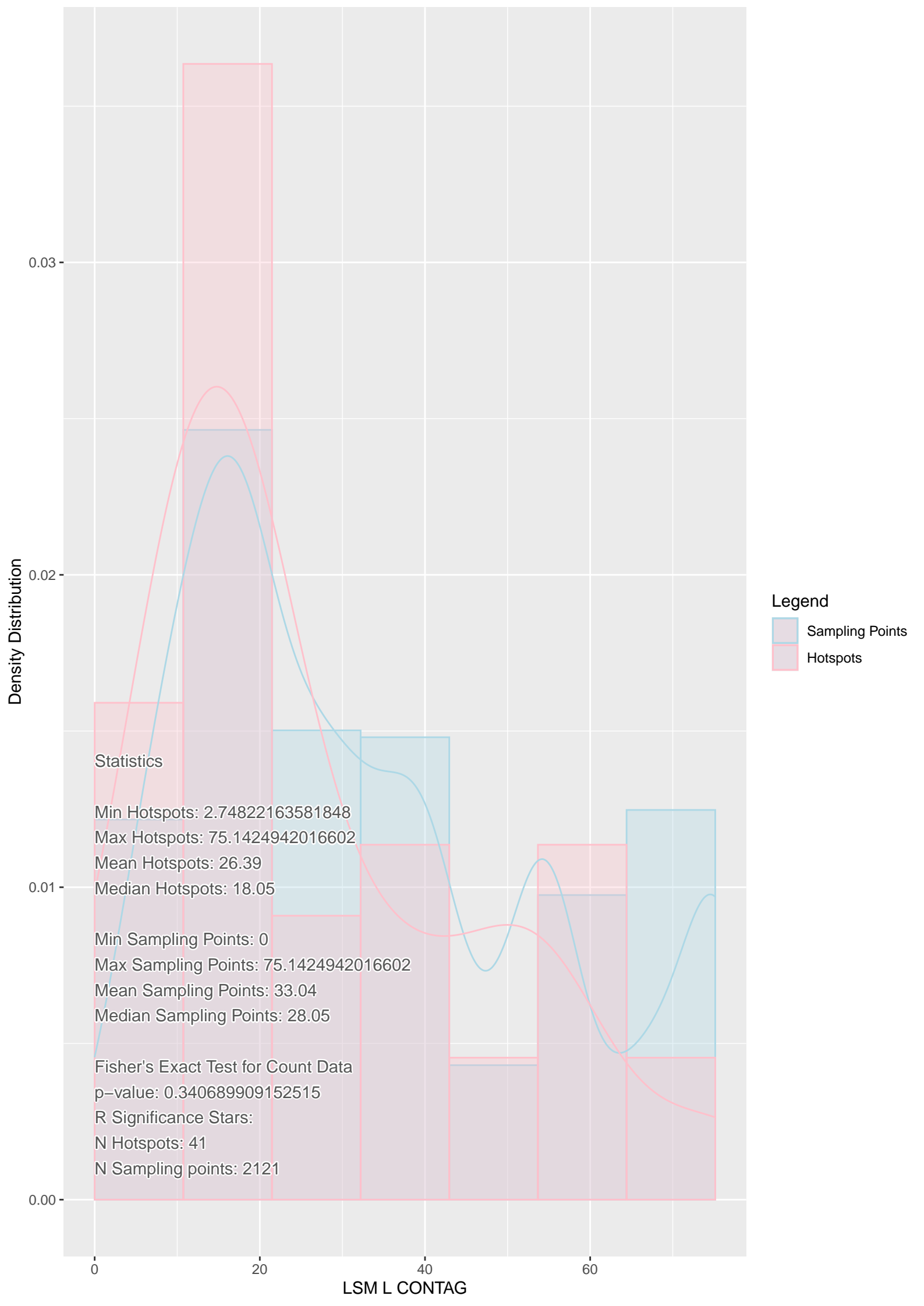

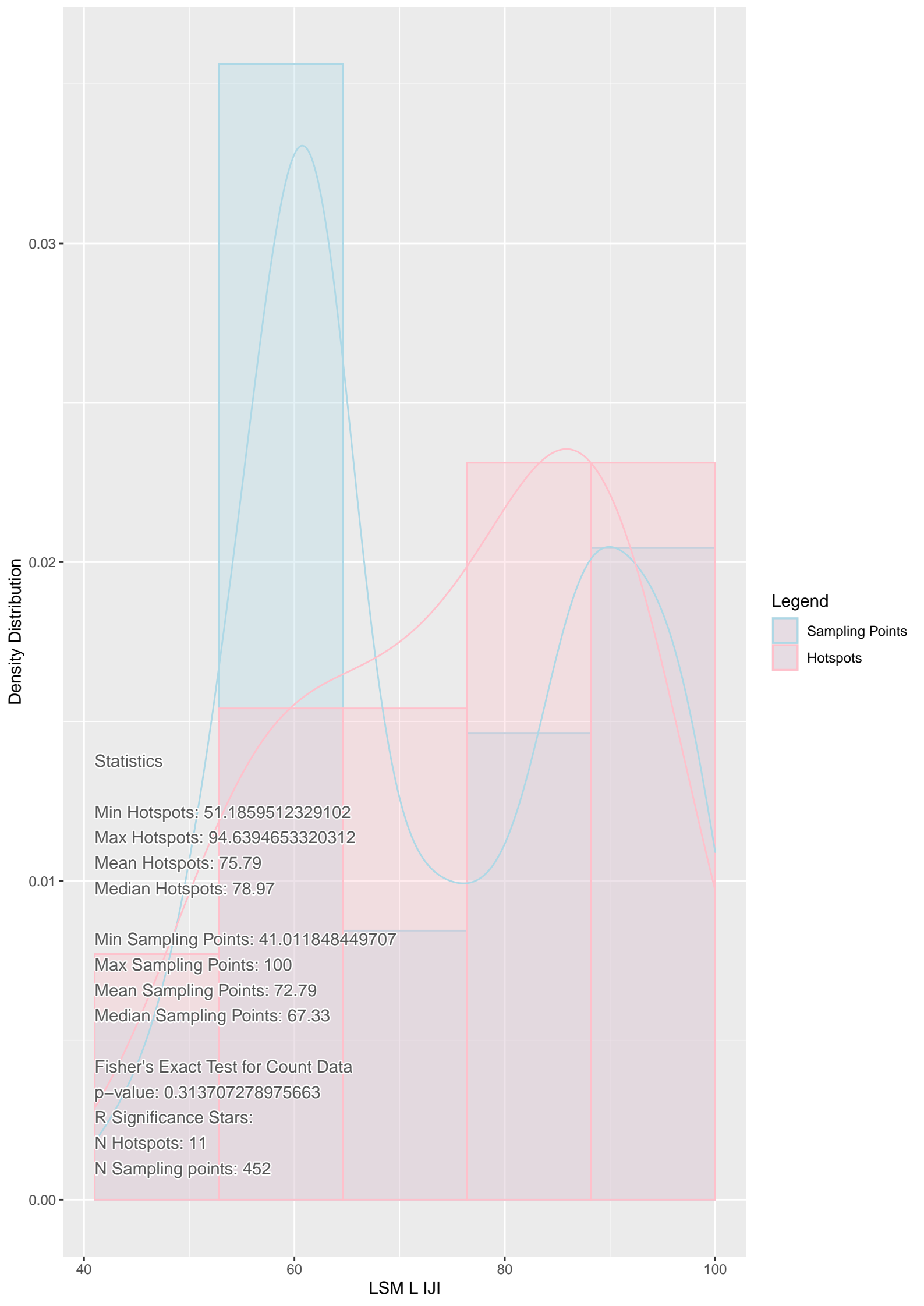

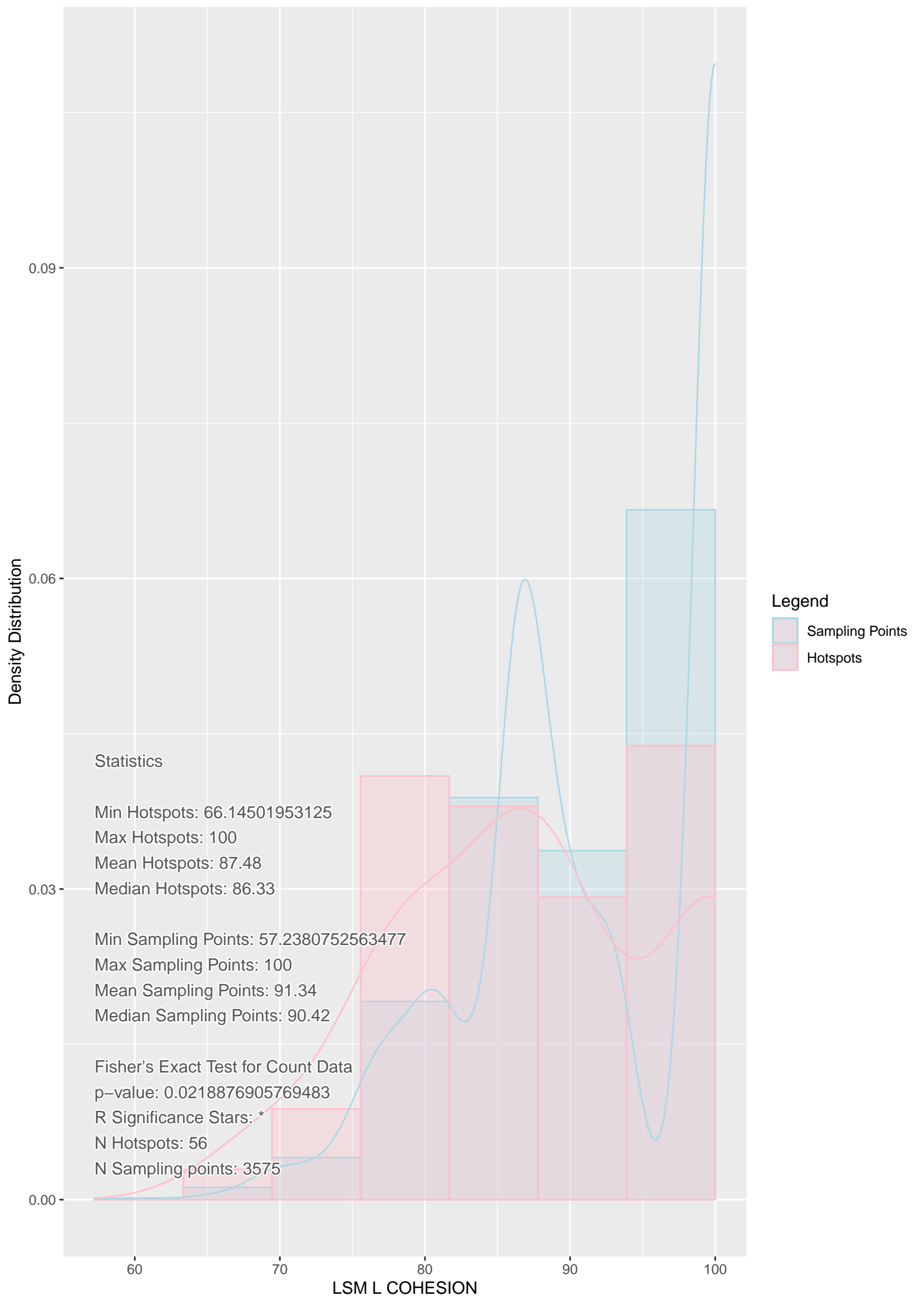

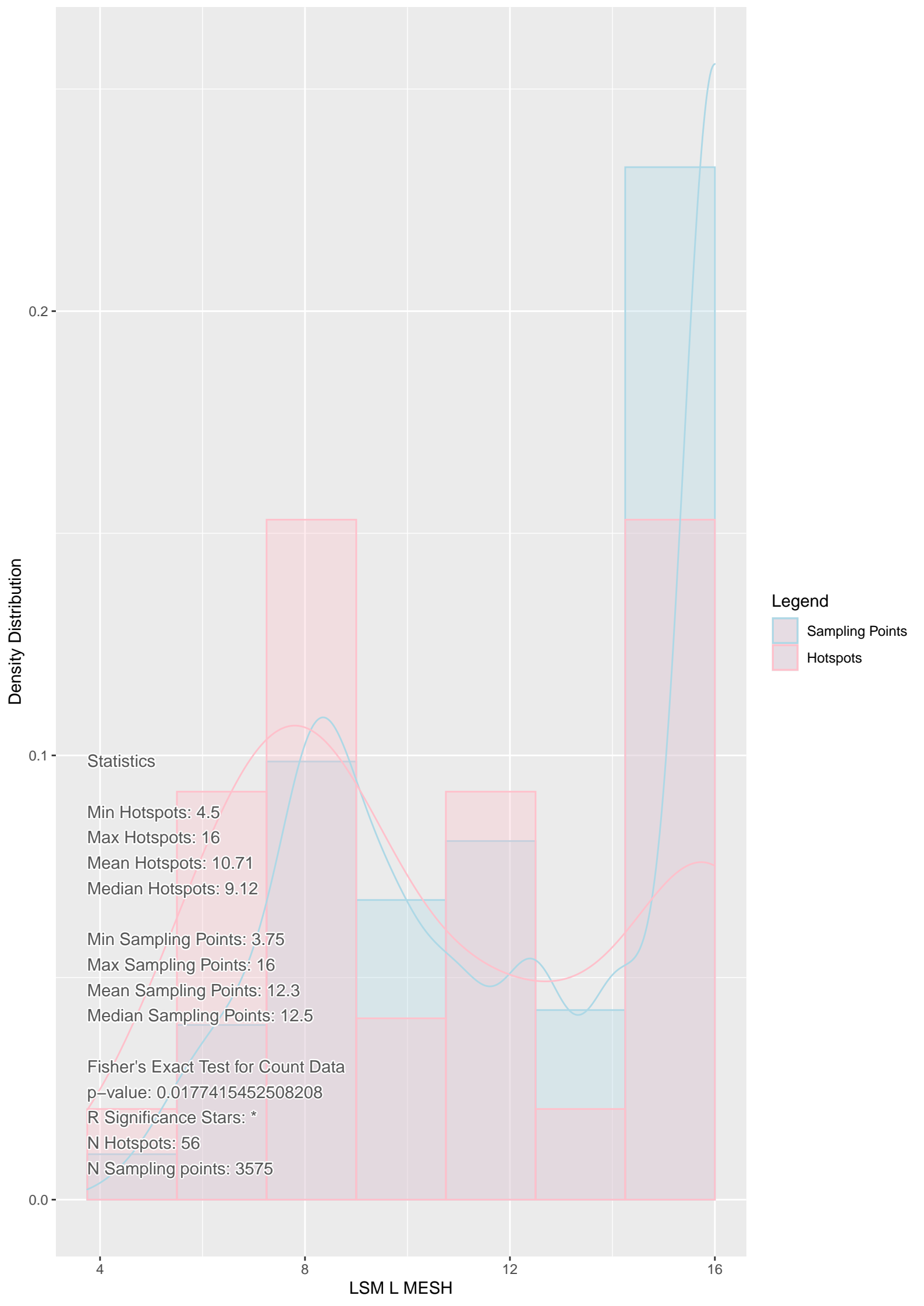

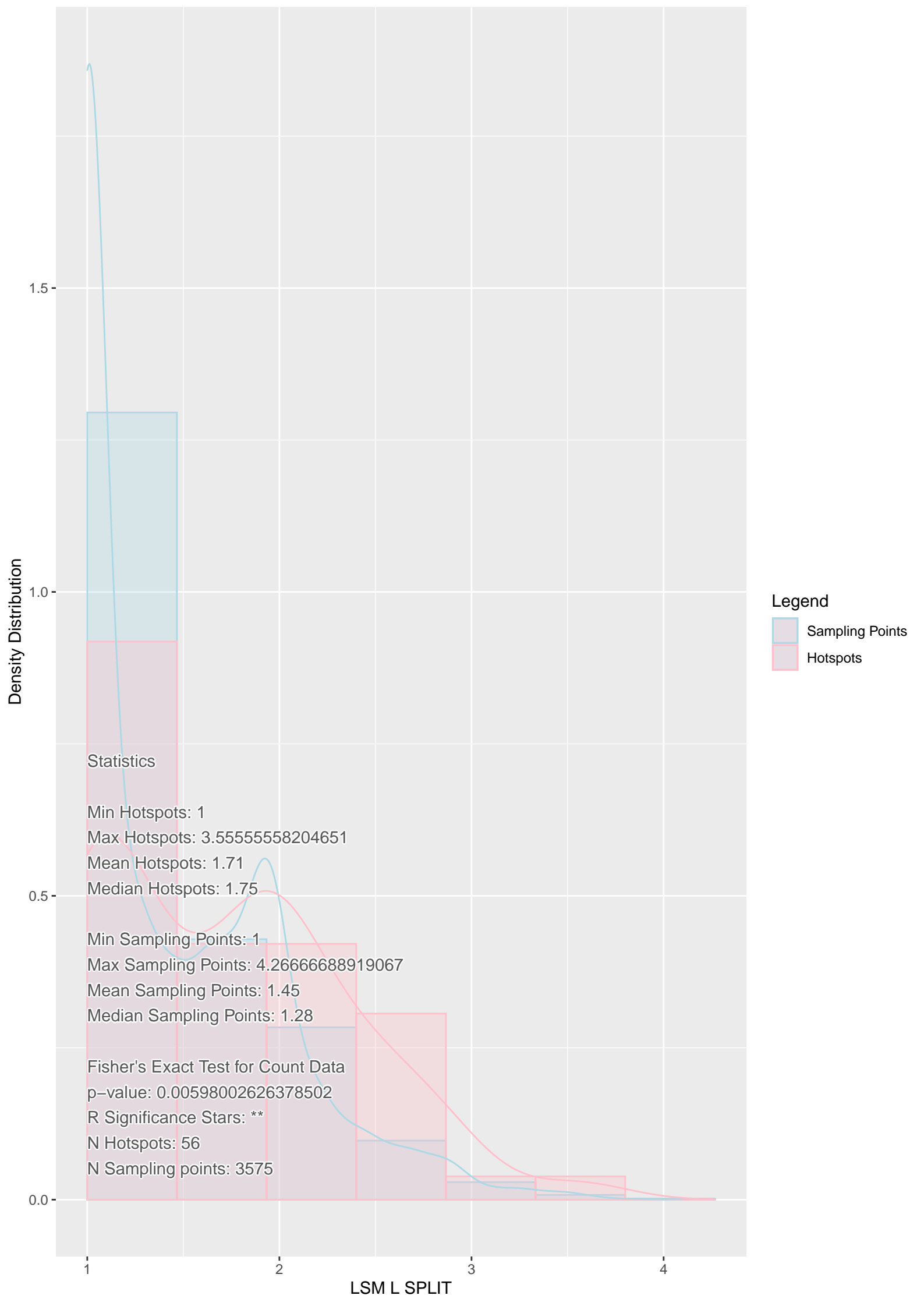

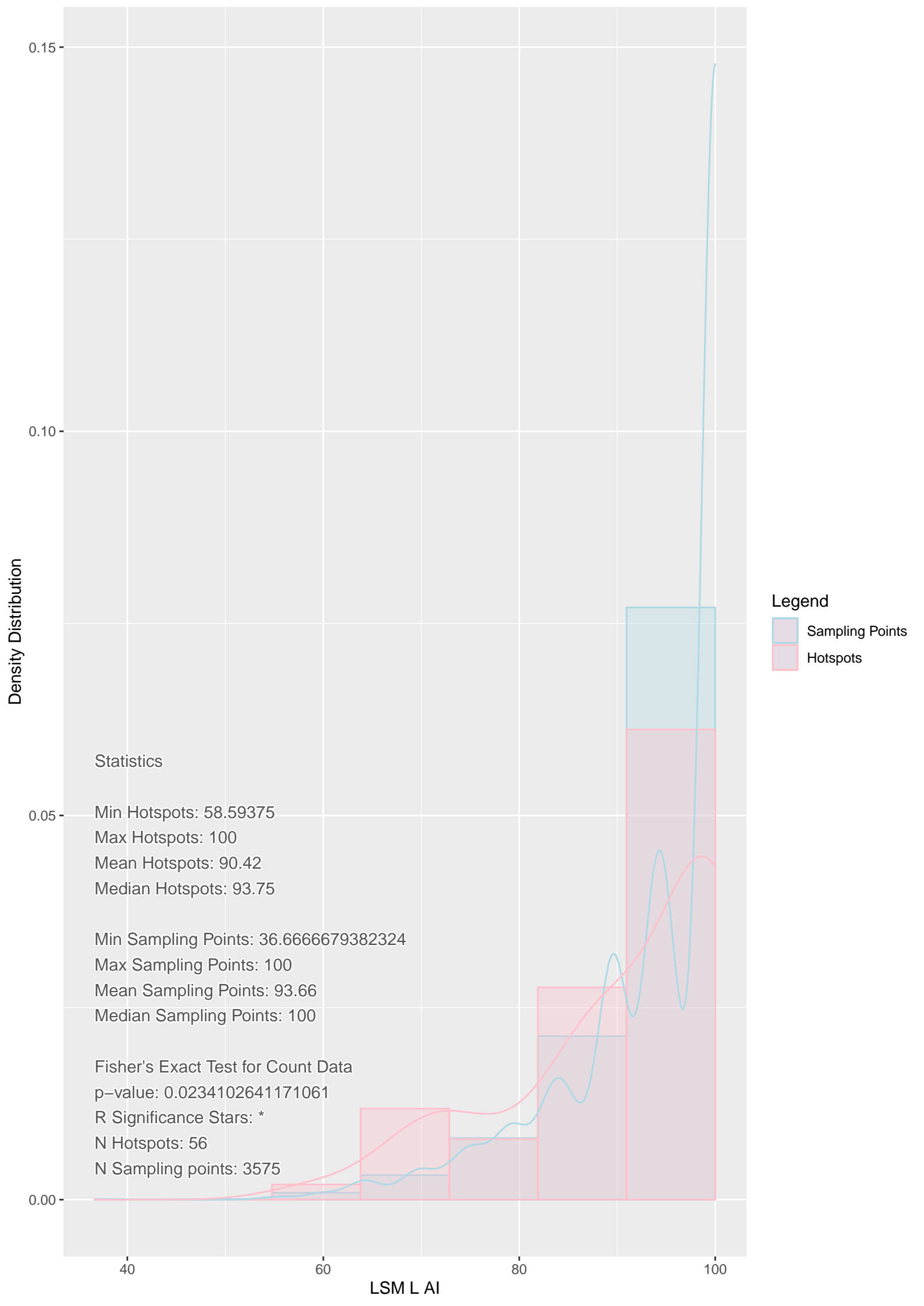

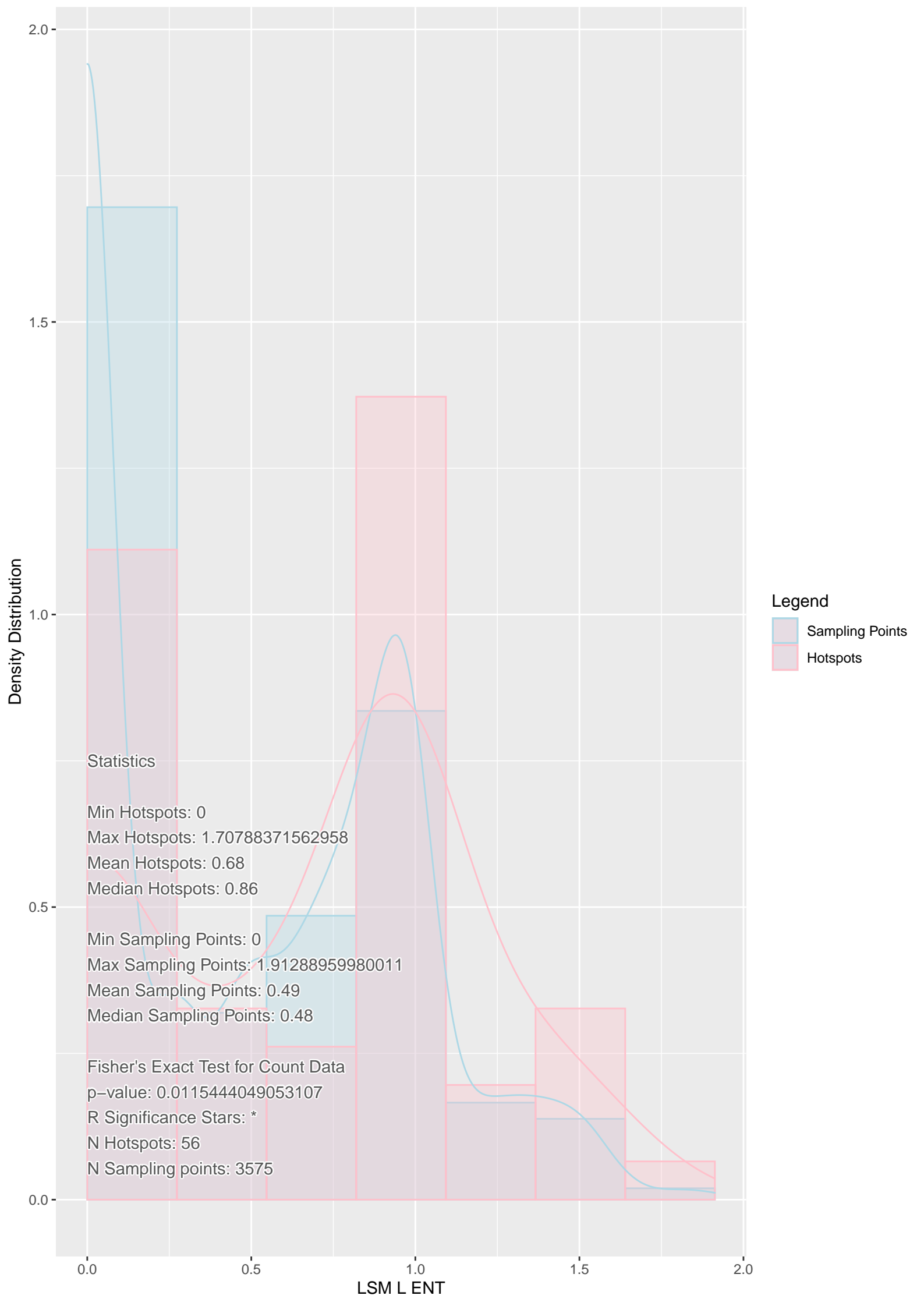

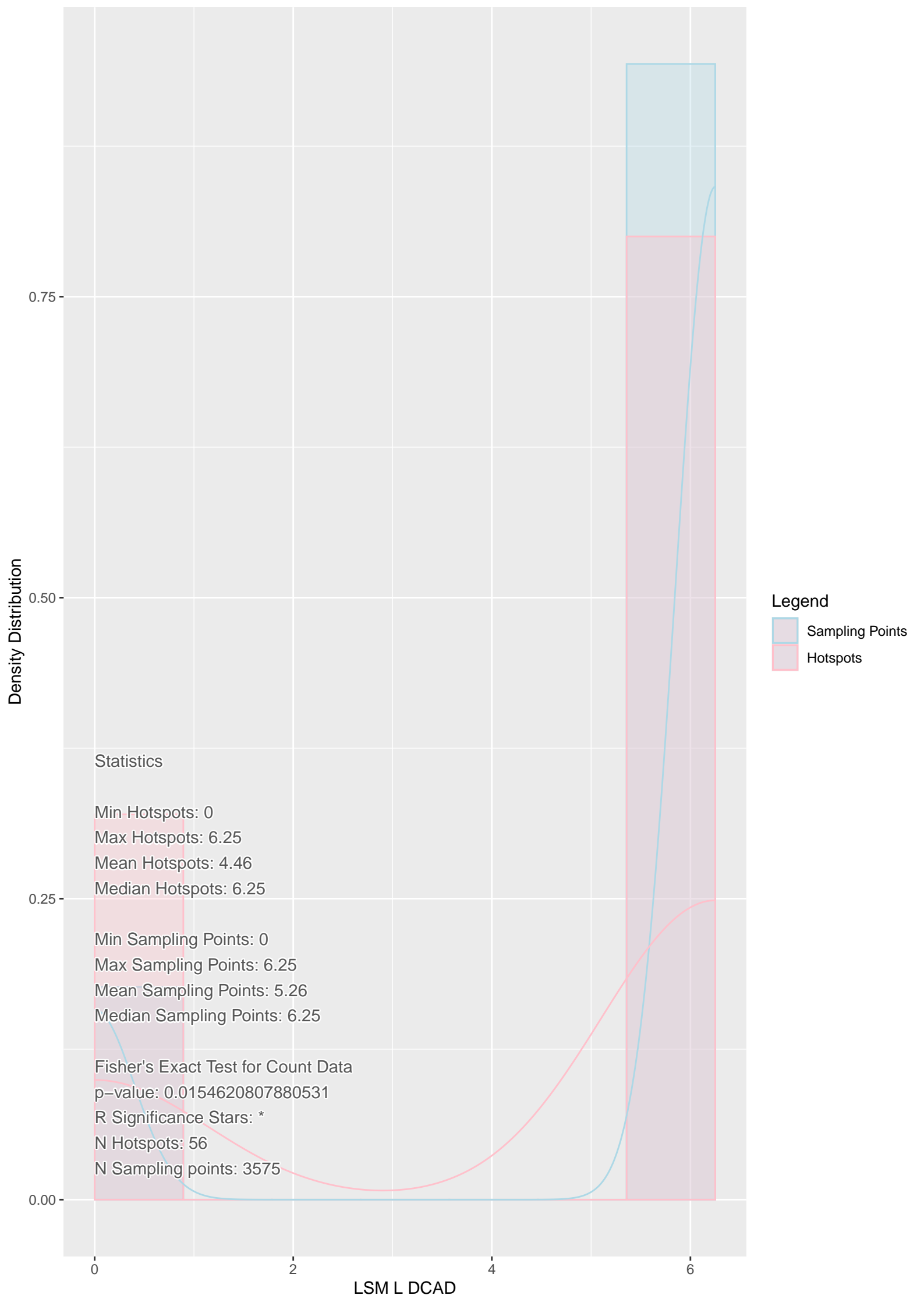

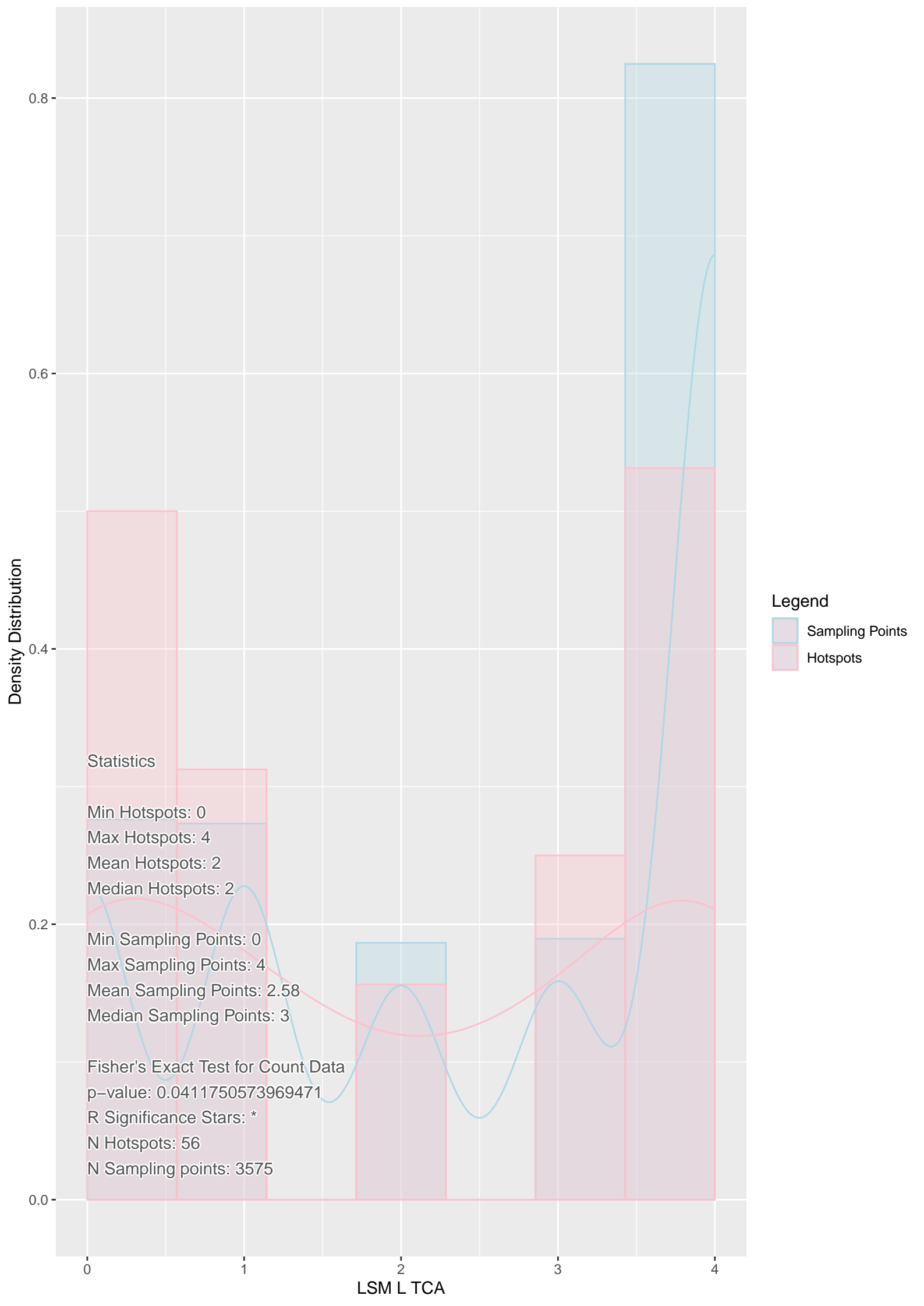

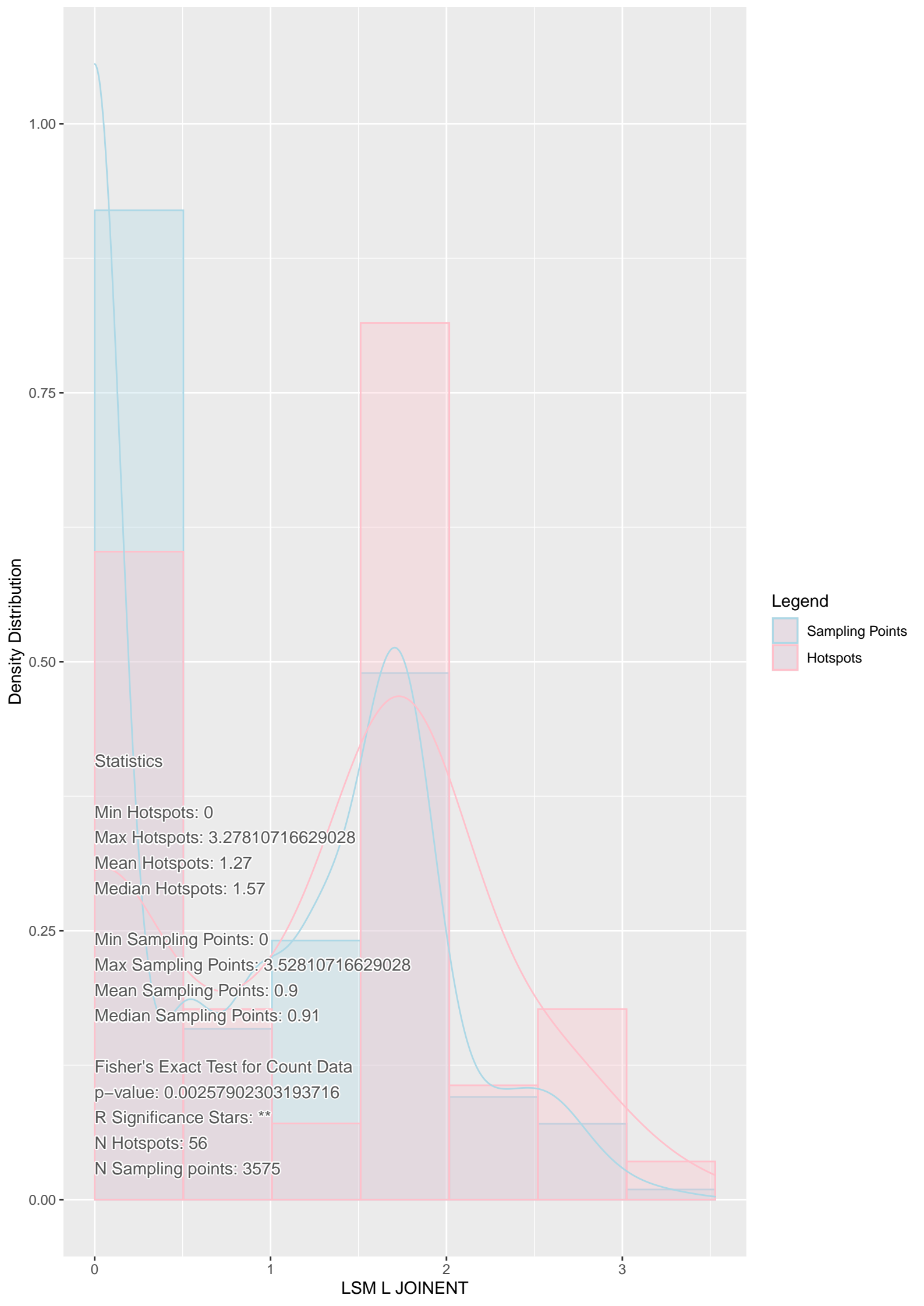

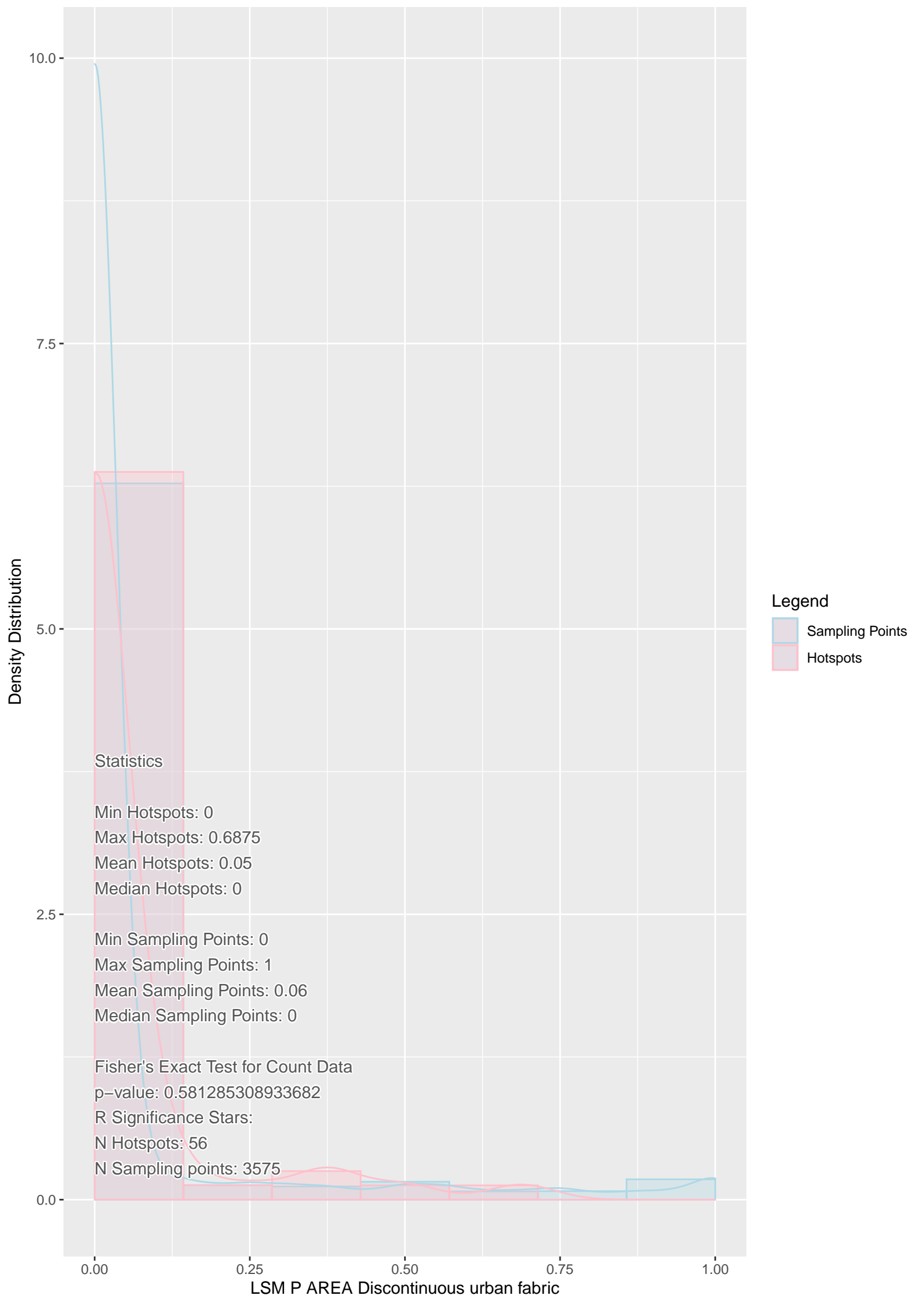

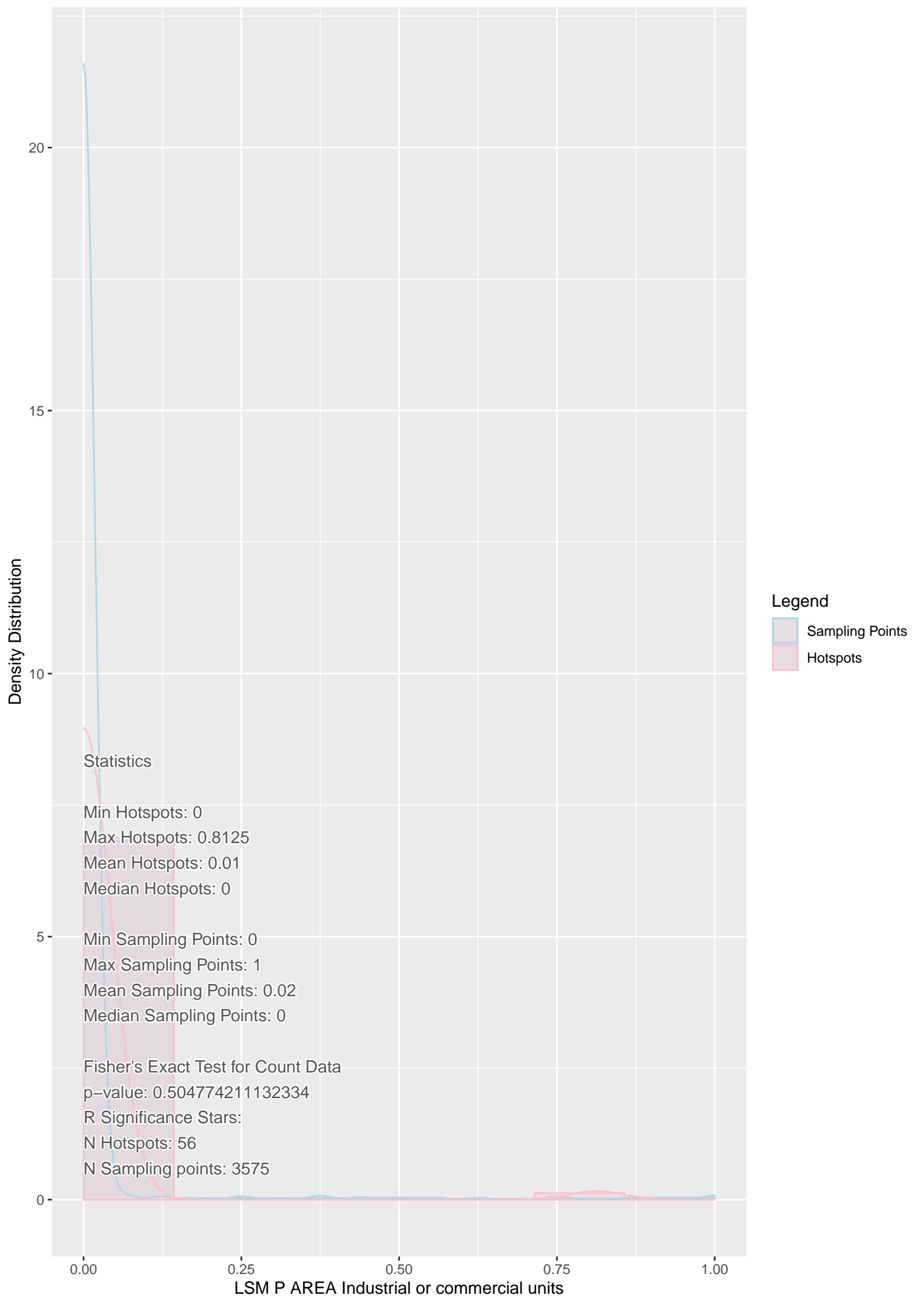

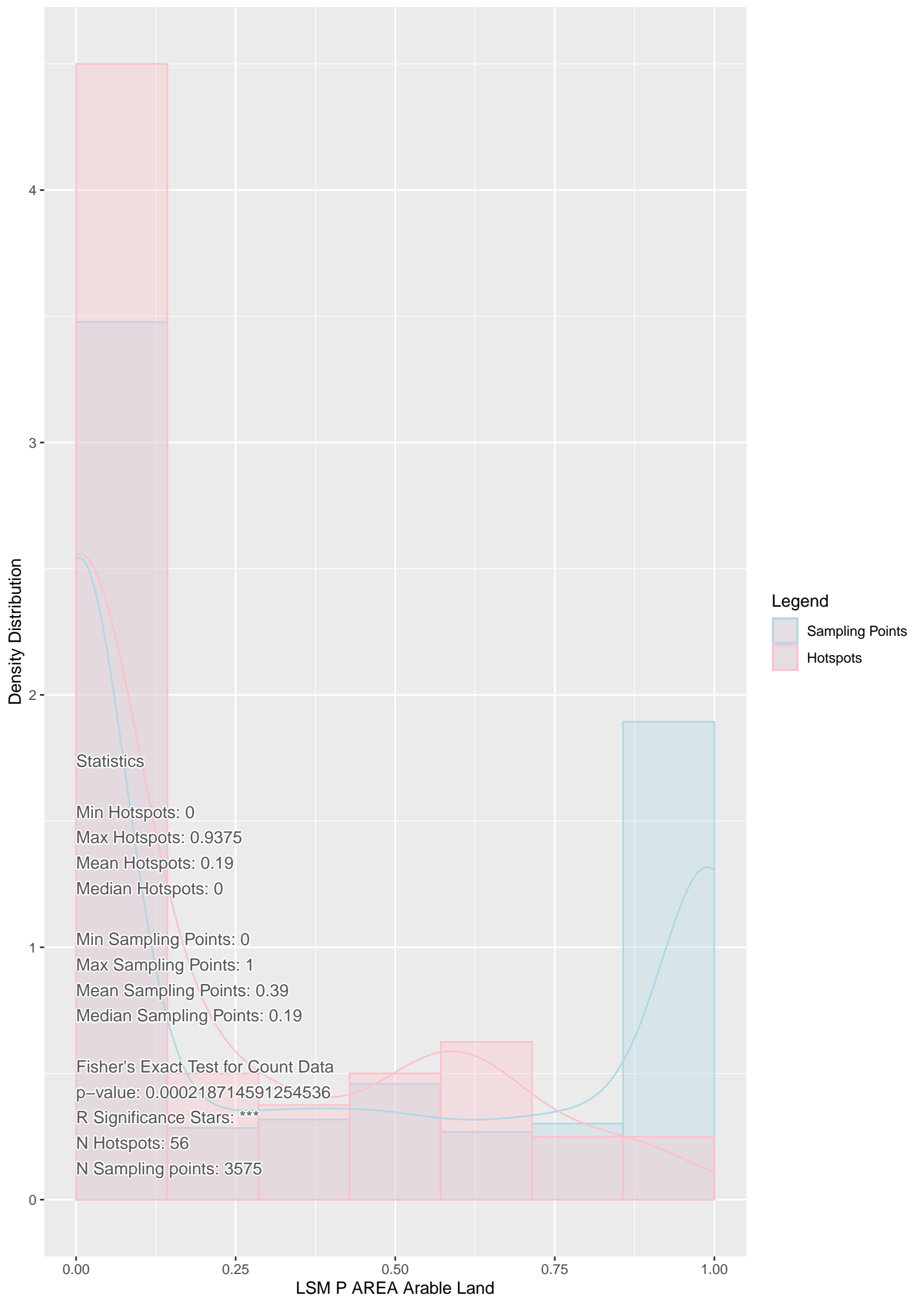

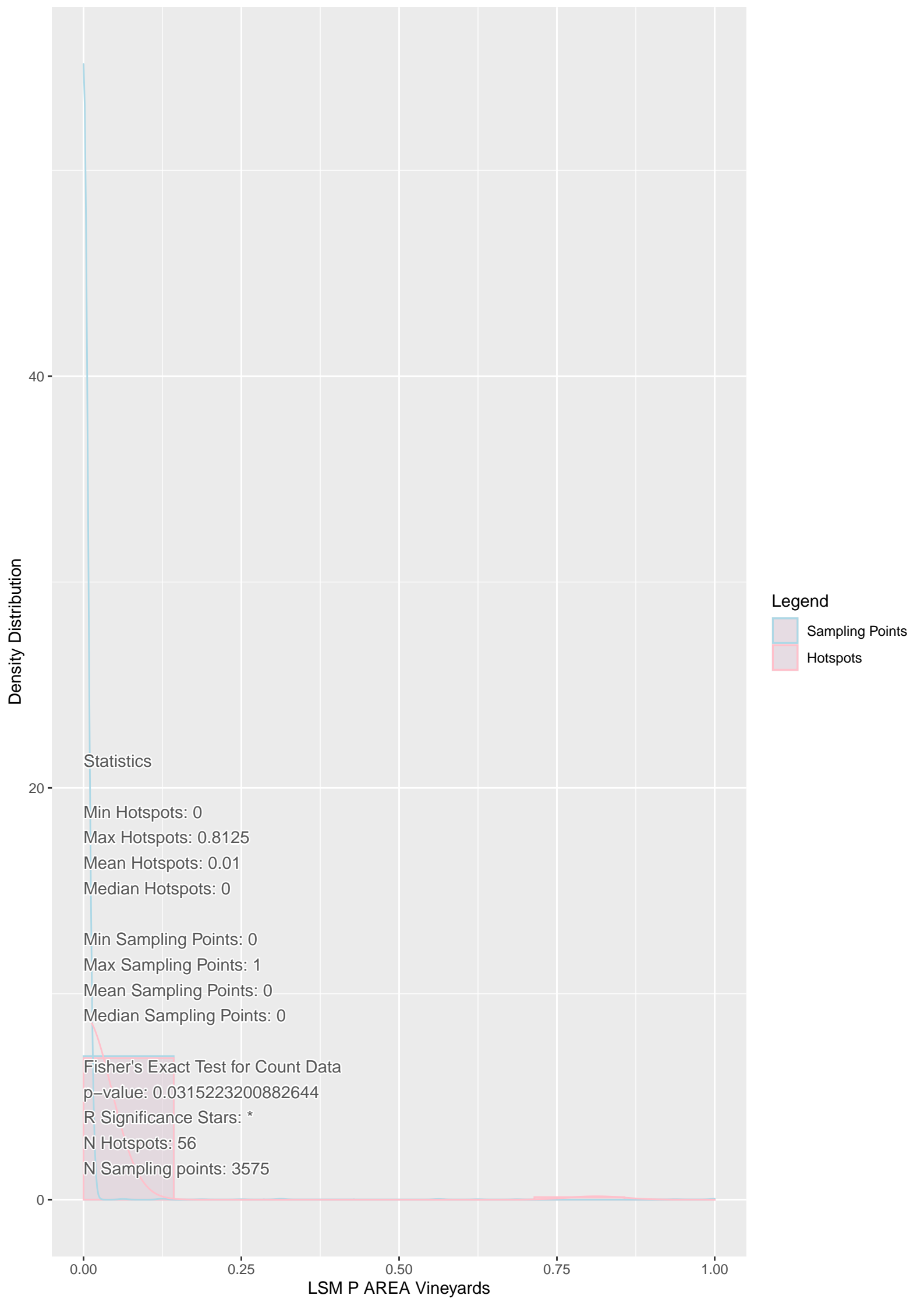

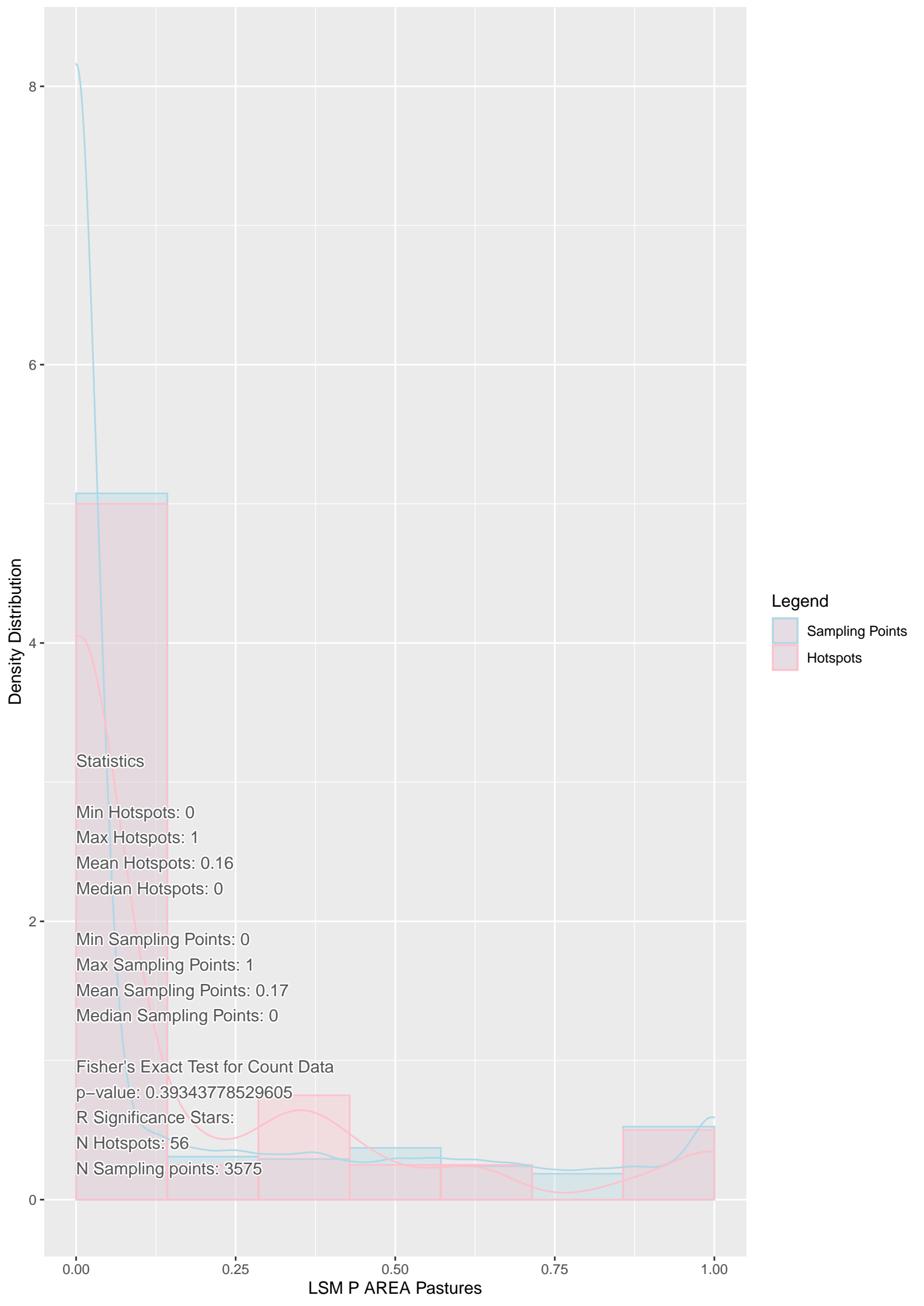

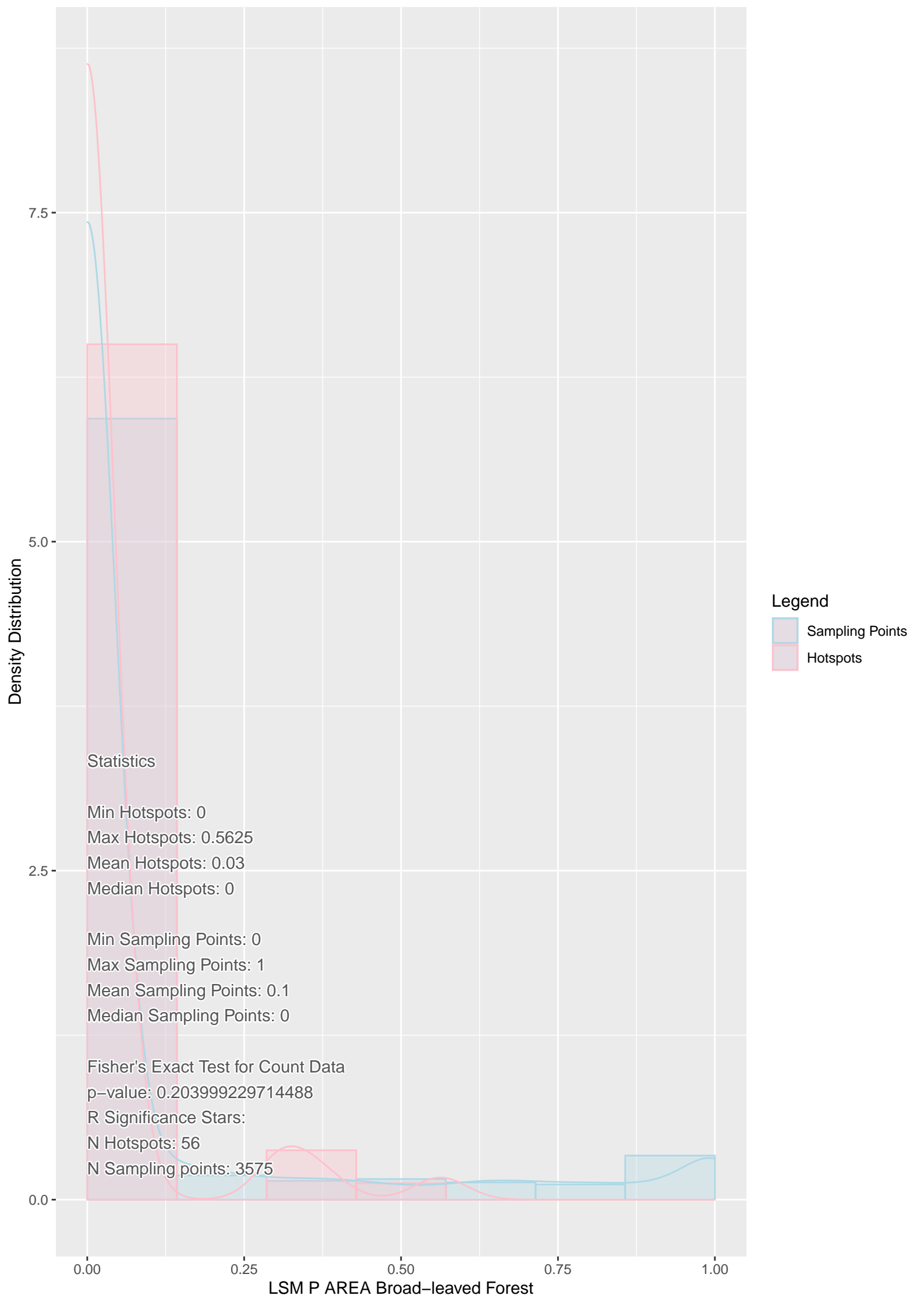

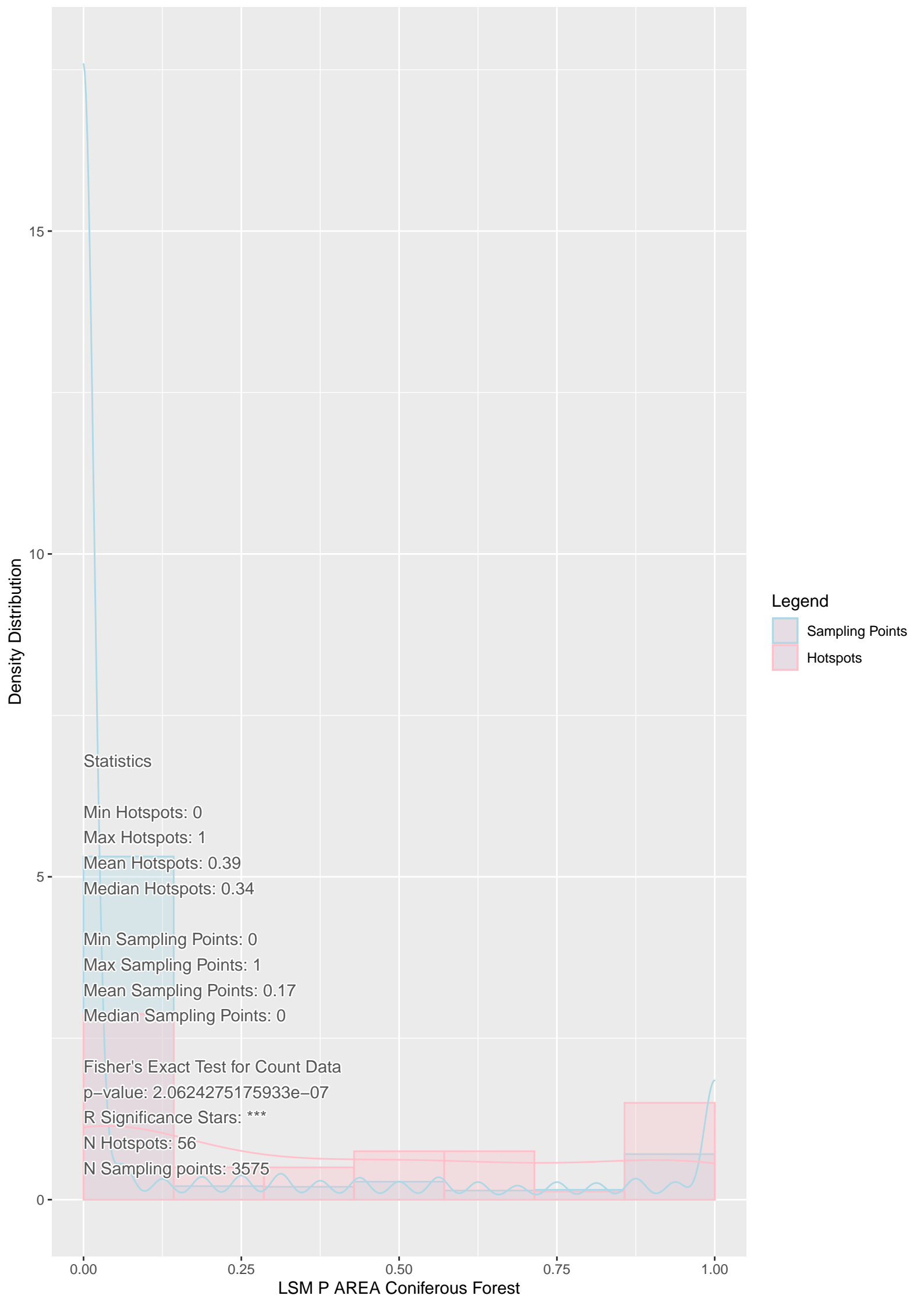

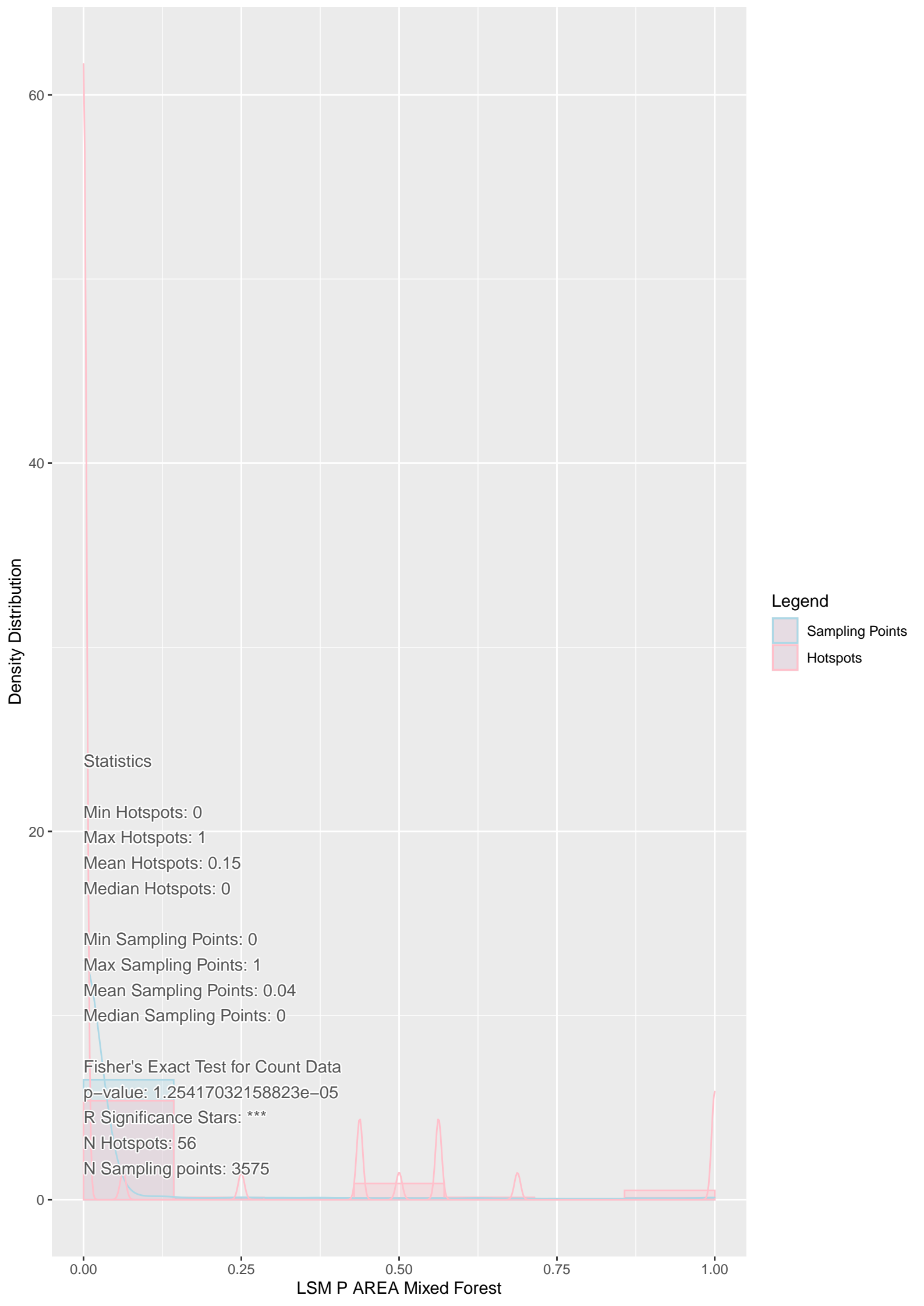

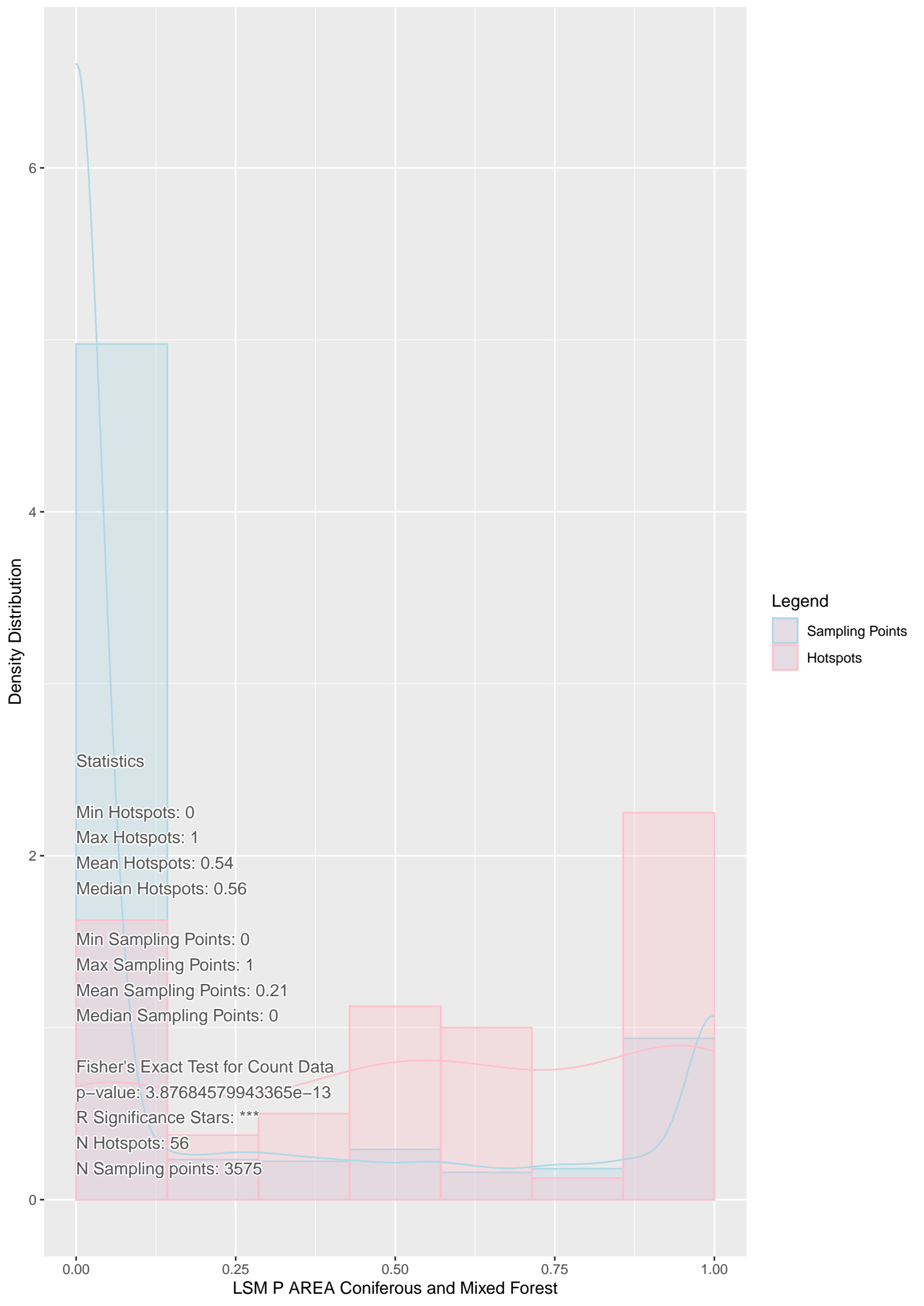

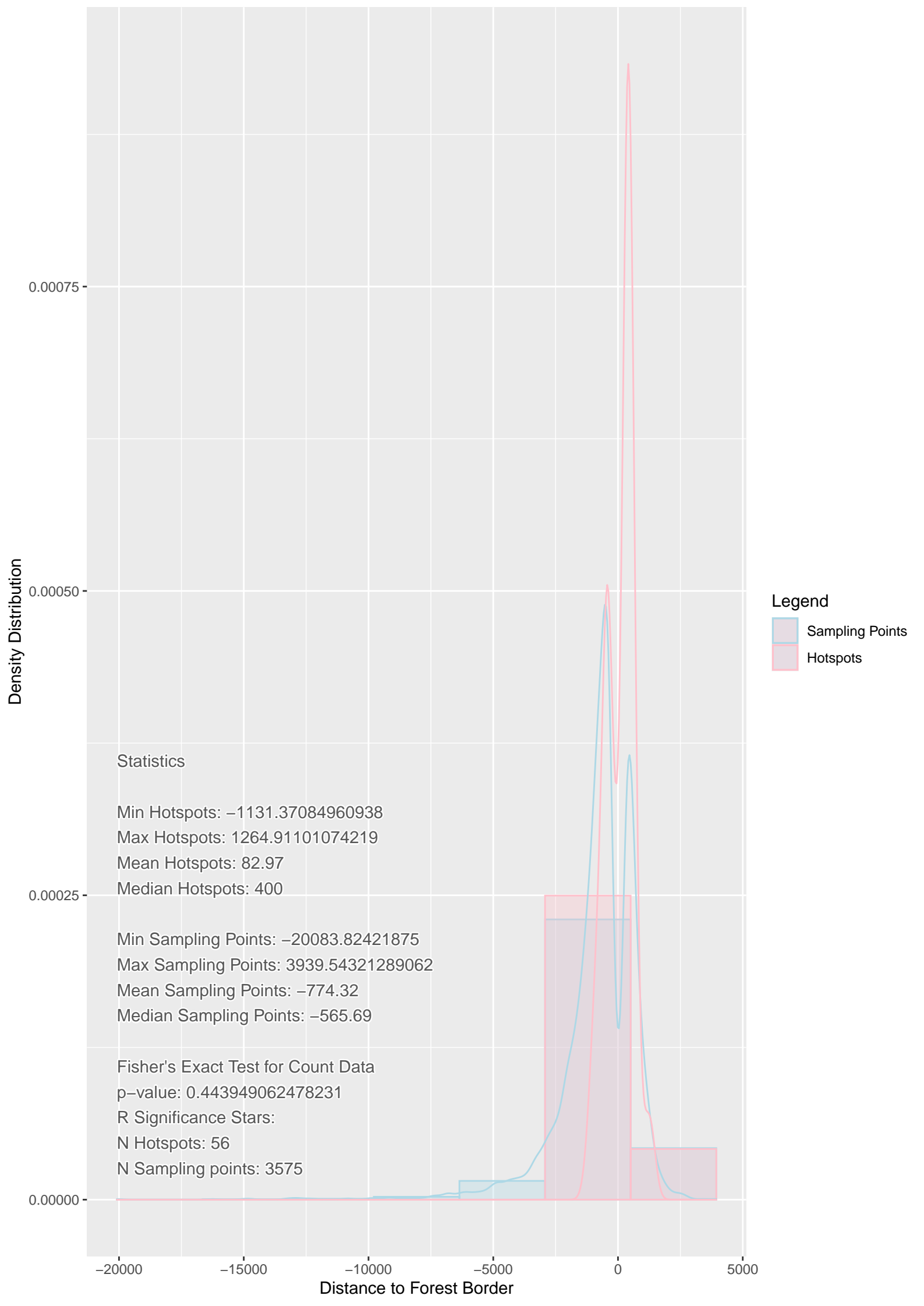

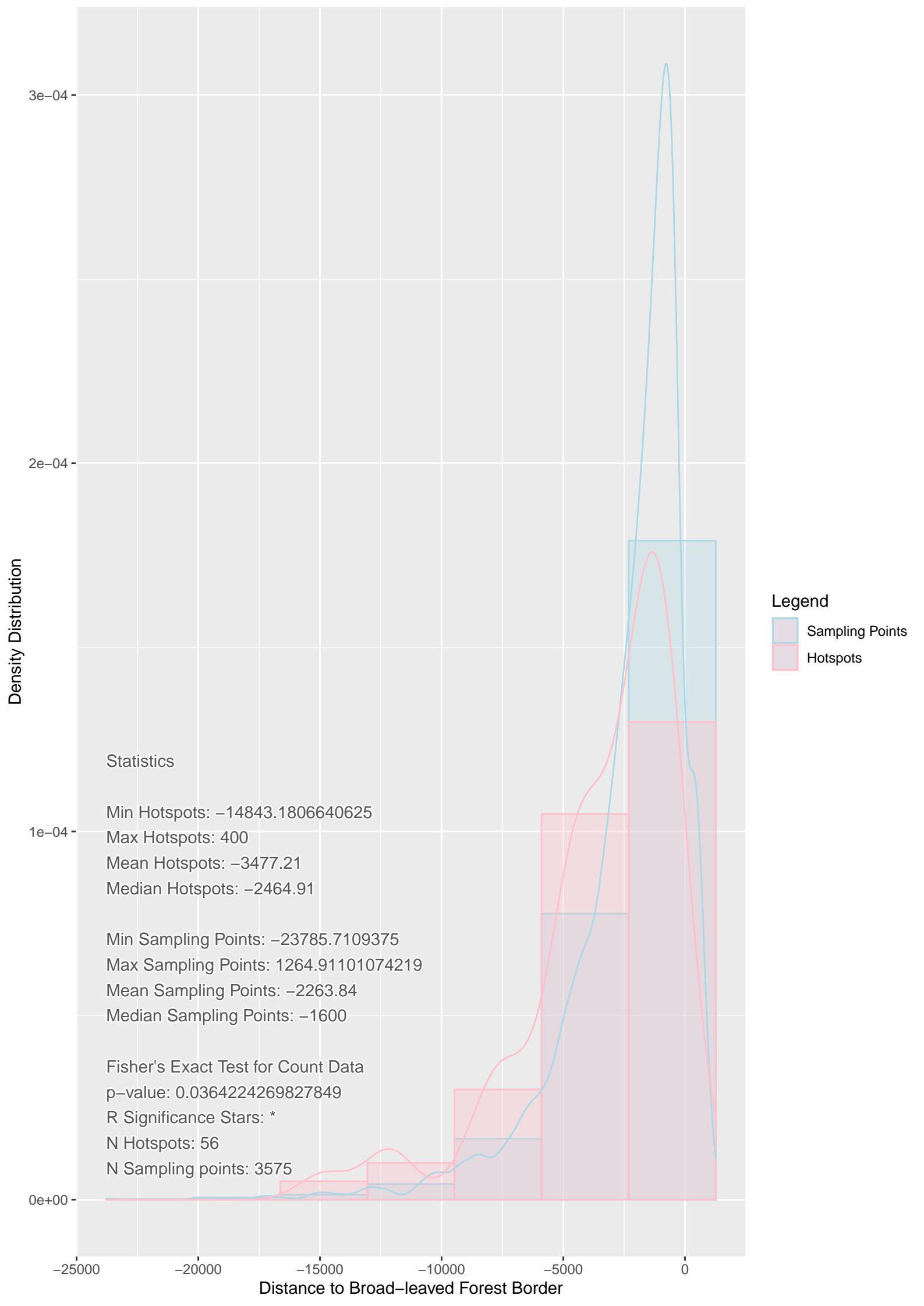

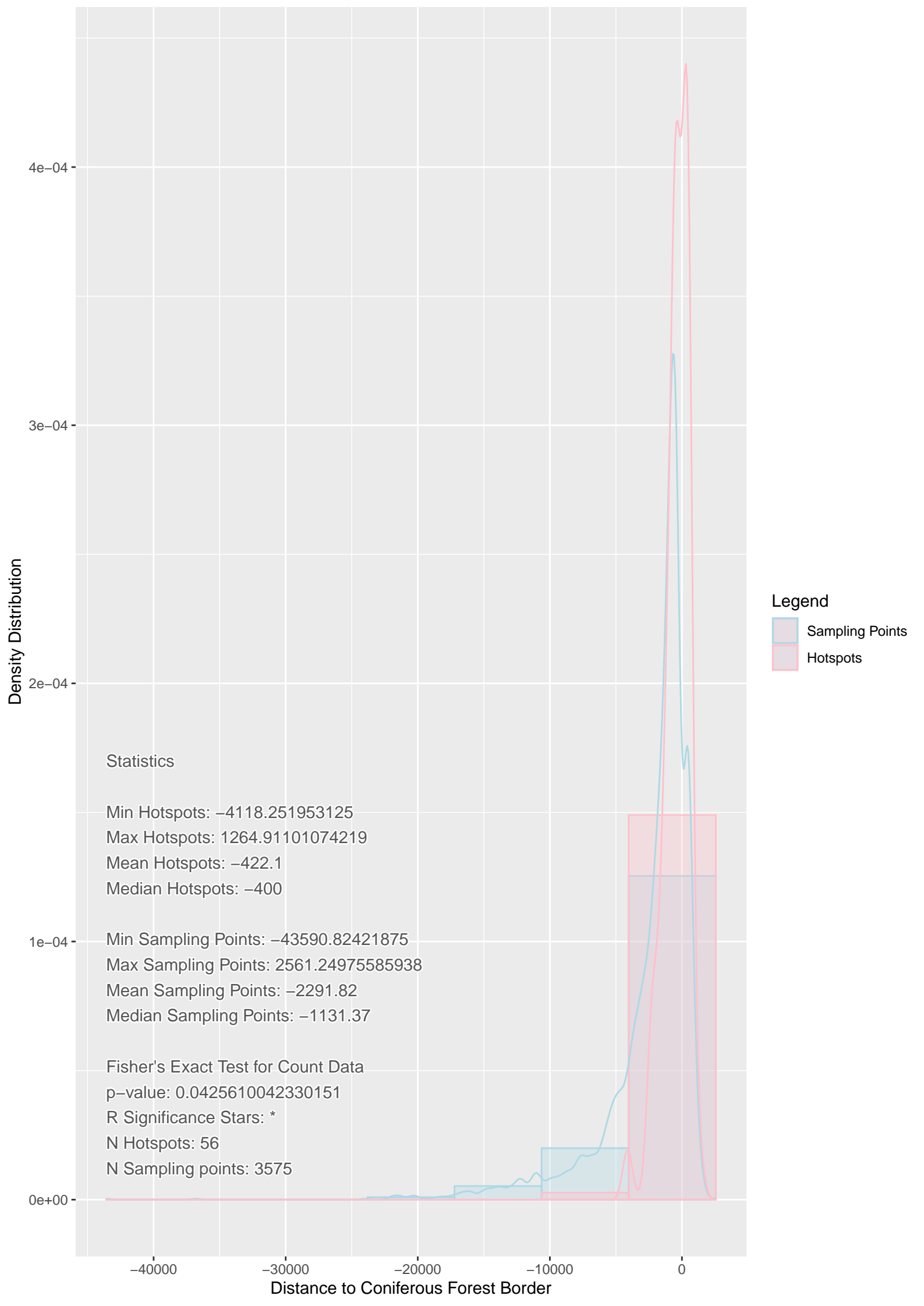

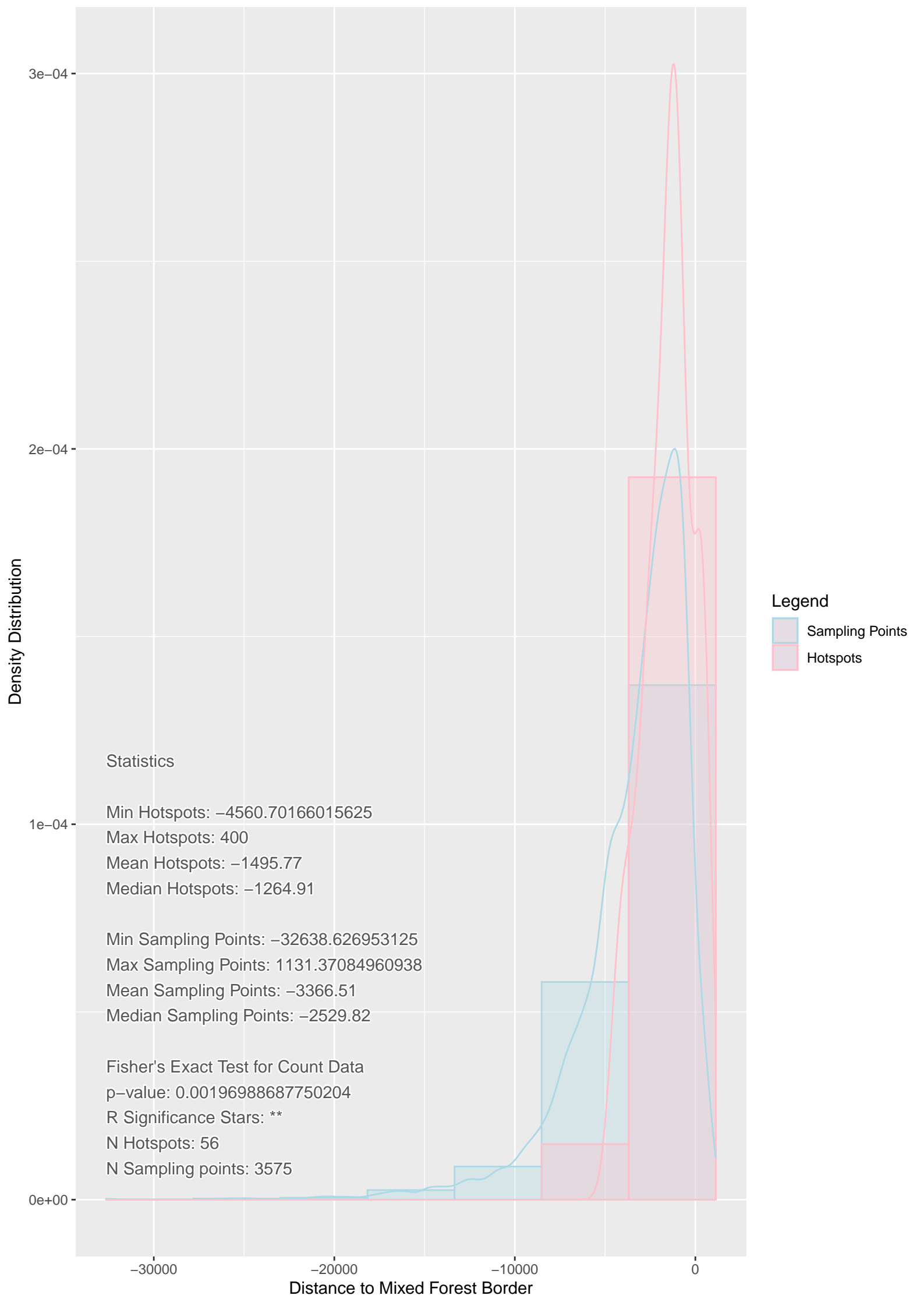

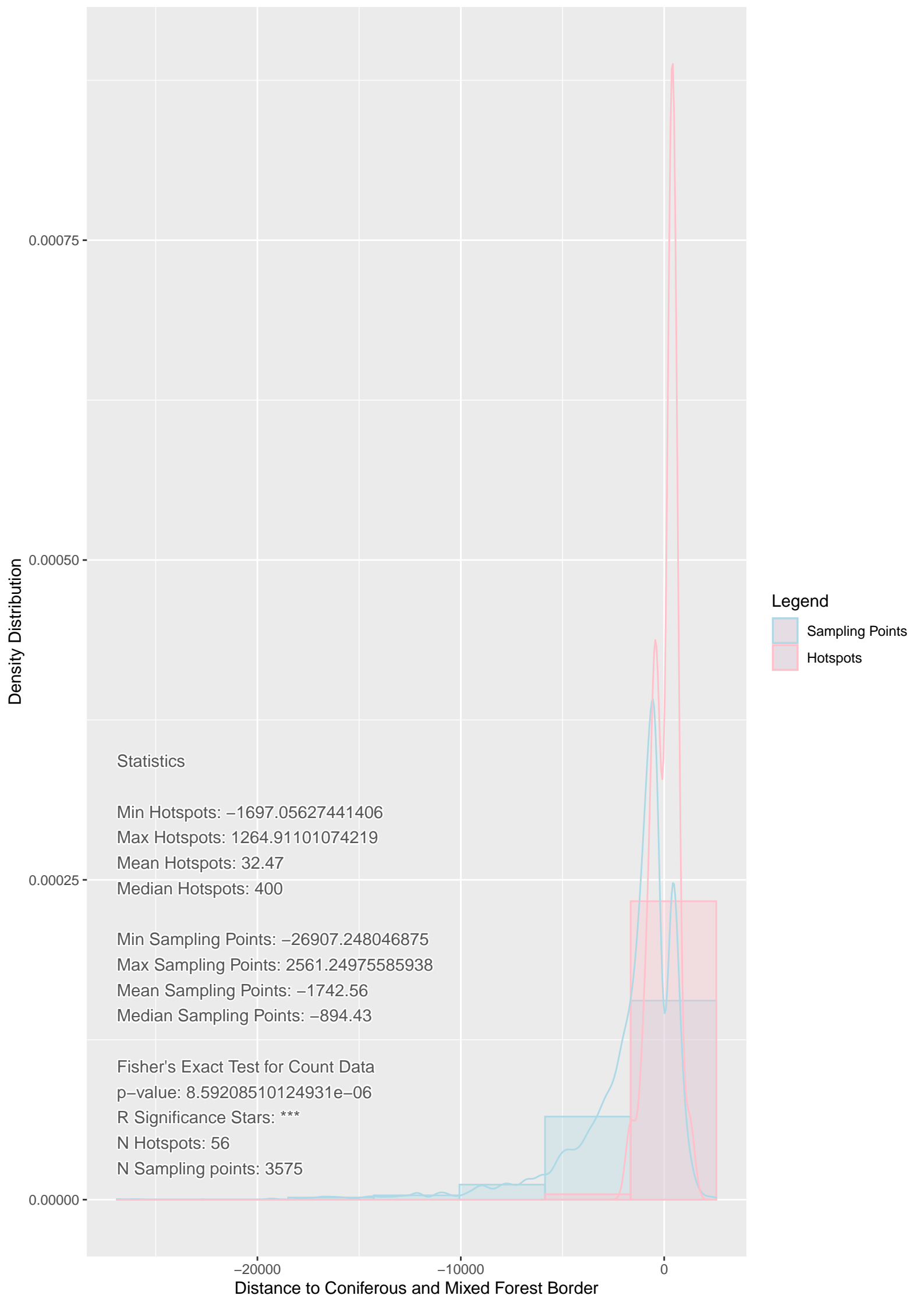

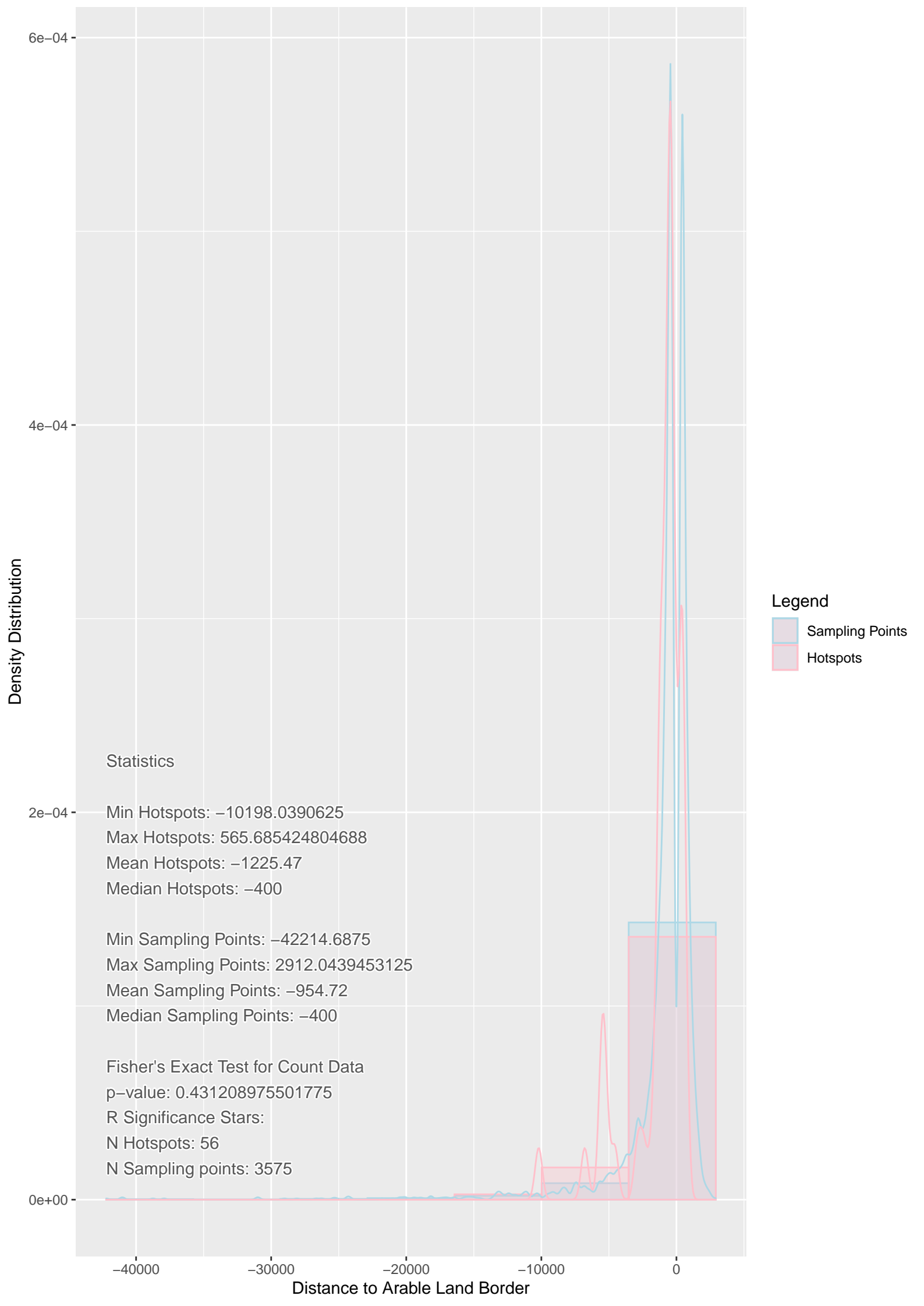

Supplement: Supplementary file 1 [file ijerph-19-11830-s001.zip › suppl. fig 3.pdf]

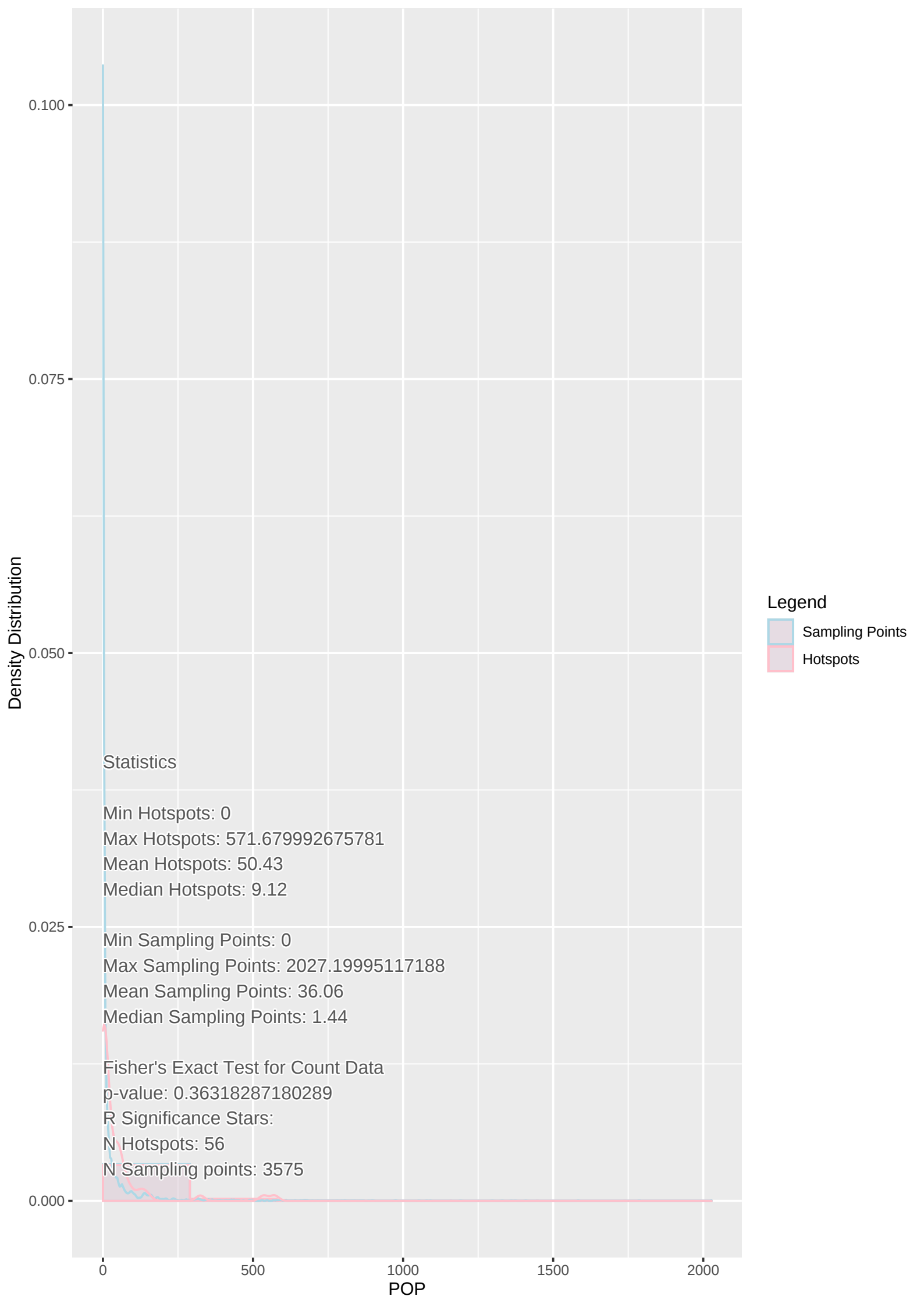

Supplement: Supplementary file 1 [file ijerph-19-11830-s001.zip › suppl. fig 4.pdf]
